# Supplementary material for: Phylogenomics of 10,575 genomes reveals evolutionary proximity between domains Bacteria and Archaea
Source: Nat Commun. 2019 Dec 2;10:5477. doi: 10.1038/s41467-019-13443-4 (PMC6889312; doi:10.1038/s41467-019-13443-4)
Supplement: Supplementary file 1 — Supplementary Information [file 41467_2019_13443_MOESM1_ESM.pdf]

Supplementary Information

Phylogenomics of 10,575 genomes reveals evolutionary proximity between domains Bacteria and Archaea

Zhu et al.

This PDF file includes:

- Supplementary Notes 1-7
- Supplementary Figures 1-27
- Supplementary Tables 1-11
- Supplementary References

Supplementary Notes

Supplementary Note 1. Novel algorithms for prototype selection

Given a distance matrix *D* for *n* objects and a given number *k*, the problem of *prototype selection* is to find a subset of $k \subset n$ objects, with $1 < k < n$, such that an objective function *d* is optimized. This problem is known to be NP-hard [1]. In the example of [2], the objects are geographical locations of *n* clients of a banking corporation. The distance matrix *D* reflects the time to clear a check drawn in client's location *i* and cashed in client's location *j*. The bank's problem is to decide for a given number *k* at what client locations to open a branch in order to maximize their available funds. Thus, the objective function is the minimization over the given distances in *D*. For our use case of choosing a most representative subset of *k* genomes, we maximize over the given distance matrix as defined by MinHash signatures [3] in order to maximize diversity. An exact algorithm must enumerate all *n* over *k* combinations of *k* objects, compute the score for every combination via objective function *d* and select optimal combination(s). Since *n* over *k* grows exponentially, this is impractical for relevant input sizes and we have to resort to heuristics. Fortunately, results of alternative heuristics implementations can be compared by their score, although it remains unclear what an optimal score would be.

We devised a naive algorithm to heuristically solve the prototype selection problem: It starts with the full set of *n* objects. The initial score for all *n* objects is the sum of pairwise distances for all objects in *n*. In each iteration, we greedily choose the one object, which reduces the overall score the least and remove it from the shrinking set. We continue until *k* is reached. We call this algorithm due its shrinking nature of maximizing overall distance score: "**destructive_maxdist**". We furthermore implemented alternative algorithms to solve the prototype selection problem. The implementation "**constructive_maxdist**" is a close relative: We start with the two objects that are most distant from each other in *D*. The set of prototypes is then constructively grown by adding the object showing largest sum of distances to all remaining objects in *D*. The method "**constructive_protoclass**" implements the algorithm of [4] but for only one "class". Intuitively, a sphere is drawn around every object in *D* with radius ε. The element whose ball covers most other objects is selected as prototype. All such covered elements and the new prototype are removed for the next round. This is repeated until no balls cover more than its center element. Our fourth and last method "**constructive_pMedian**" implements the *p-median* algorithm of [5] which is closely related to *k*-means clustering for given *k*.

Our comparison of those four implementations of heuristic algorithms to solve the prototype selection problem shows that "destructive_maxdist" requires least run time, returns highest scores for many instances and can handle instances of n = 90,000 within seconds (Supplementary Fig. 1b-d).

For our application, we needed to extend the original problem definition by allowing the pre-definition of r objects as prototypes, a.k.a., "*seeds*". Thus, k - r prototypes need to be selected from n objects such that all objects of r are guaranteed to become prototypes. This alternation will preserve objects of biological interest while minimizing the reduction of score. For example, we wanted to make sure that several well-studied *E. coli* strains are chosen over other thousands of less popular ones. The algorithms work as described above, but in the initiation phase, the set of selected prototypes is not empty but filled with r objects and corresponding rows and columns in D are masked. The increase in runtime is marginal with this function enabled, while the resulting score is notably higher than that by not using this function (Supplementary Fig. 1e).

Python implementations are provided at https://github.com/biocore/wol/, under directory code/prototypeSelection, which also contains a Jupyter notebook we used for benchmarking.

Supplementary Note 2. Comparative analysis of trees by different methods and input data

We conducted a systematic exploration of the optimal strategy for building the microbial tree of life. Multiple species trees were reconstructed, using differential taxon, gene and site sampling strategies, as well as different tree-building methods, implementations and evolution models. The comparative analysis results are detailed in this section. Two metrics were mainly used for comparing trees: 1) The Robinson-Foulds (RF) distance [6] normalized by tree sizes, which measures the topological discrepancy between each pair of trees; 2) "tip distance" (TT), which measures the correlation between tip-to-tip distance matrices of two trees (see Methods). In addition, the distributions of branch support values, if comparable and relevant, were addressed. To maximize objectiveness, these analyses are purely based on the mathematical properties of trees and are free from any biological knowledge.

Comparison between "full-scale" ASTRAL and CONCAT trees. The two tree-building methods produced similar species tree topologies (Fig. 3). The distance between the two CONCAT trees is shorter (RF = 0.179) than between either of them and the ASTRAL tree (see below), which is expected considering the differential mechanisms behind each method. The CONCAT tree based on randomly sampled sites ("concat.rand") resembles the ASTRAL tree (RF = 0.260) more than does the CONCAT tree based on most conserved sites ("concat.cons") (RF = 0.312), likely a consequence of random site sampling, which better represents the full-length sequence alignments that were used for building individual gene trees for ASTRAL. Additionally, the species tree built on all sites but using FastTree ("fasttree") shows higher similarity with the random CONCAT tree (RF = 0.156) than with the ASTRAL tree (RF = 0.257), implicating higher impact by tree-building method than by robustness of the same method (further discussed below, see Supplementary Fig. 12). Interestingly, the tree based on ribosomal proteins ("concat.rpls") is more similar with the ASTRAL tree (RF = 0.253) than with the CONCAT trees (RF = 0.340 (conserved) / 0.304 (random)).

The two methods for building large phylogenies have different computational requirements (Supplementary Table 2). As mentioned before, computer memory and run-time constraints limit the size of the datasets and the complexity of the models that can be analyzed with CONCAT. On the other hand, the gene tree summary method implemented in ASTRAL is less constrained, even though its overall cost is greater, because most of the time is spent in building individual gene trees, a step that can be fully parallelized across compute nodes. This scalability of ASTRAL means that it can be extended in a straightforward manner to even larger scale phylogenomic analyses than considered here.

We estimated branch lengths for the ASTRAL tree using either most conserved or randomly selected sites (see Methods). Even though random site sampling gave a larger tree dimension overall than conserved site sampling, the individual branch lengths had strong linear correlation between the two methods (slope = 1.776, $R^2 = 0.974$, $p = 0.0$).

Evaluation of trees inferred using implicit vs. explicit methods. We tested three alternative approaches for assessing the relationships among organisms: namely, either explicit (gene tree summary or gene alignment concatenation) or implicit (by marker gene distribution, MinHash signature, andor NCBI taxonomy. Albeit simple and applicable approaches, they do not explicitly model the evolutionary process of molecular sequences. The topological distances among these trees and the species trees reconstructed using dedicated phylogenetic approaches are shown in Supplementary Fig. 7. It reveals high discrepancy among the three implicit trees and from the explicit category (RF > 0.62). In particular, the taxonomy has the highest discrepancy (RF > 0.83), due to its over-simplified hierarchies. Meanwhile, the four phylogenetic trees, despite using different gene selection, site sampling and tree-building methods, notably converge better (RF < 0.35). Topologies were compared using the Robinson–Foulds (RF) metric [6]. The topologies of the trees built using explicit methods, either summary or concatenation, are better converged than those obtained from the alternate, cheaper methods, which do not directly operate on sequence data (Supplementary Fig. 7a). This underscores the necessity of using sequence data and dedicated phylogenetic approaches to accurately define evolutionary relationships in high-quality phylogenomic studies.

Impact of gene tree quality and quantity on ASTRAL trees. We evaluated whether a large number of loci, i.e., the practice of "phylogenomics" is essential in resolving species evolution using ASTRAL, which is based on the summary of multiple gene tree topologies. The 381 marker genes were randomly downsampled to smaller sets, on each of which an ASTRAL tree was built. We observed a slightly increased level of deviation from the original, full-scale ASTRAL tree (Supplementary Fig. 10a). With 200 gene trees (around half of the original 381), the topology differed by RF = 0.081. Meanwhile, the branch supports (local posterior probabilities) continued to increase with the number of gene trees (Supplementary Fig. 10c) and did not plateau even with 381 gene trees, suggesting the benefit of including more loci in resolving species phylogeny.

We also assessed the influence of gene tree quality on the ASTRAL tree. Four trees were generated for each marker gene: one by FastTree, and the other three by RAxML, either based on the FastTree starting tree or two random seeds (see Methods). The reference ASTRAL tree was built using the best scoring RAxML gene tree of the three. As alternatives, we built two more ASTRAL trees, either based on the

FastTree-started RAxML trees, or the initial FastTree trees. We observed low levels of topological discrepancy from the reference ASTRAL tree (RF = 0.048 and 0.090, respectively) (Supplementary Fig. 10b) and very close branch support distributions (Supplementary Fig. 10d).

Impact of taxon sampling on species phylogeny. A long-standing dilemma for phylogeneticists is to balance among the number of taxa, the number of sites, and the robustness of algorithm, subject to realistic computational limitations. Fewer taxa allow the use of more expensive methods (further discussed below and in the main text, also see Supplementary Fig. 23), at the cost of losing signals that would otherwise be helpful in better defining the evolutionary relationships among clades. To test the impact of reduced taxon sampling on the species tree, we downsampled from the original 10,575 genomes to a series of fewer taxa, in each case maximizing the representativeness of the deep phylogeny of bacterial and archaeal evolution (see Methods). The three robust phylogenetic methods—ASTRAL, CONCAT conserved, and CONCAT random (which produced Supplementary Figs. 4-6, respectively)—were applied to each taxon set.

As the taxon number decreased, the reconstructed topology gradually deviated from that of the full tree (Supplementary Fig. 13a, first row of each panel). This trend was more obvious in the CONCAT trees (conserved: RF = 0.138 to 0.551, random: RF = 0.110 to 0.384) than in the ASTRAL trees (RF = 0.056 to 0.296) (Supplementary Fig. 13a, comparing among panels), suggesting that ASTRAL produced more stable topologies with taxon downsampling. Meanwhile, the deviation among trees by the three methods increased as the taxon number decreased (sum of RF = 0.752 to 1.653) (Supplementary Fig. 13b). These results suggest that taxon sampling does have an impact on the tree topology. Although ASTRAL appears to be more resistant to this effect than CONCAT, it still suffered with an RF = 0.103 (which translates into 10.3% incongruent clades) when the taxon number went from 10,575 down to 1,000. Therefore, the quantity of taxa is important in assessing the deep phylogeny.

Impact of site sampling and alternative models on CONCAT trees. Because of the computational expense of CONCAT with RAxML, we had to truncate the concatenated sequence alignment to at most 100 sites per marker gene (see Methods), leaving approximately 38k sites in total. Although this was more than eight times as many as the PhyloPhlAn default (on average 12 sites per gene, or 4.5k sites in total), there is a considerable loss of signals from the 192k-site full alignment. Meanwhile, the "trident" algorithm implemented in PhyloPhlAn enabled selection of the most conserved sites, compensating for the potential alignment inaccuracy in the full alignment which may be deleterious in the subsequent phylogenetic inference. To assess the influence of site sampling on the species tree, we used the trident

algorithm to sequentially select 100, 50, and 25 sites per gene, plus the PhyloPhlAn default (~12), and compared the CONCAT species trees generated in each case.

Simultaneously, we evaluated the two alternative methods for modeling rate heterogeneity among sites: Gamma (classical and expensive) and CAT (a faster and less memory-intensive approximation to Gamma, which produces likelihood values than cannot be compared between analyses) [7]. (Note that the rate heterogeneity discussed here should not be confused with the more complex, profile mixture models discussed below.) Due to computational constraints, RAxML analyses were not feasible with the Gamma model on more than 25 sites per gene or with the CAT model on more than 100 sites per gene. Whereas the CONCAT trees discussed in the main text were based on 100 sites per gene with the CAT method (see Methods), here we also consider trees based on either 25 sites per gene or the default setting with the Gamma model.

We observed a pattern of sequential shift in both topology and among-taxa distances along with site sampling (Supplementary Fig. 11). From the default setting to 100 sites per gene, there was an RF = 0.308 and a TT = 0.099 (which translates into a Pearson correlation coefficient of 0.802). This sequence moves toward the two trees built on randomly selected sites or all sites (the later was built using FastTree, which is further discussed below, see also Supplementary Fig. 12). The patterns suggest that site sampling does have an impact on the phylogenetic trees. Therefore we chose to discuss both CONCAT trees using most conserved or randomly selected sites in interpreting the biology behind the trees. Furthermore, we noted that the choice of CAT vs. Gamma model had low impact on tree topology and phylogenetic distances (RF = 0.040 and 0.127, TT = 0.00121 and 0.0046, 25 sites per gene and default).

Impact of non-vertical evolution on species phylogeny. Conventional molecular phylogenetics analyses usually attempt to avoid loci that are prone to horizontal gene transfer (HGT), which is prevalent in the microbial world and affects a large range of genes [8,9]. One major advantage of ASTRAL is its robustness to HGTs, allowing us to include as many as 381 gene trees to achieve optimal species tree accuracy. To validate this assumption in the context of this study, we performed a test, in which the marker genes were downsampled based on the quartet score of the corresponding gene tree—a measurement of the consistency between gene and species evolution. We selected four quartet scores thresholds: 0.5, 0.67, 0.75 and 0.8, and performed both ASTRAL and CONCAT (using conserved or random sites) species tree reconstruction on subsets of marker genes above each threshold. The results show that with fewer but presumably more "vertically evolving" genes, the ASTRAL trees retained notably more consistent topologies (smaller RF distance) than CONCAT trees did (Supplementary Fig.

22a). When all species trees were included in one matrix, we observed close clustering of the ASTRAL trees, in contrast to the diverse distribution of CONCAT trees (Supplementary Fig. 22b, c) (ASTRAL vs. CONCAT conserved / random, PERMANOVA pseudo-F = 5.612 / 5.571, p-value = 0.009 / 0.007). These observations suggest that ASTRAL is significantly more robust against gene tree discordance compared to CONCAT.

We next checked the branch supports of the ASTRAL trees. A moderate decrease along with fewer gene trees was observed (Supplementary Fig. 22d), despite the increased overall concordance of the remaining gene trees. Together with the discussion above (see also Supplementary Fig. 7), this again suggests the benefit of using a large number of gene trees in an ASTRAL analysis.

Evaluation of species trees built using site heterogenerous models on 1,000 taxa. The classical site homogenerous substitution model (usually referred to as Gamma or +G) [7] has been widely used in phylogenetics studies, including most modern efforts for building the microbial tree of life (e.g., [10,11]). It assumes that all sites are subject to the same evolutionary process, with rate heterogeneity following a Gamma distribution. However studies have shown that this simplified assumption is prone to the long branch attraction (LBA) artefacts, especially with deep phylogenetic trees where large variations of evolutionary process are likely present [12,13]. To confirm the robustness of our findings based on the use of the Gamma model, we also built CONCAT trees using the profile mixture model C60, which was shown more robust against LBA [14], together with the posterior mean site frequency (PMSF) method implemented in IQ-TREE which enables relatively large-scale analysis with this complex model [13]. Yet this method is still notably more expensive than our reference approach, and limited our analysis to only 1,000 downsampled taxa (described above, see also Supplementary Fig. 13). For comparison, we built additional CONCAT trees on these 1,000 taxa, using either the classical Gamma model, or the FreeRate model, which relaxes from the assumption of Gamma distribution of rates [15]. We also included the 10,575-taxon trees pruned to the 1,000 taxa for comparison.

This analysis provided an alternative and highly controlled 1,000-taxon test set to compare models (Supplementary Fig. 23) and to re-assess a series of questions discussed above. There was a relatively stable disparity between pairs of trees by conserved and random site sampling, in both topology (RF = 0.201 ± 0.011, mean and std. dev., same below) and phylogenetic distances (TT = 0.0439 ± 0.0022), with PMSF not being exceptional (Supplementary Fig. 23a). Similarly, there was a relatively stable (but more variable than between site sampling) disparity between trees by 10,575 or 1,000 taxa, both built using the Gamma model (RF = 0.268 ± 0.041, TT = 0.0173 ± 0.0100), with random site sampling being most consistent (Supplementary Fig. 23b). These observations largely support the findings discussion

above (see also Supplementary Figs. 11 and 13). Interestingly, the topological inconsistency introduced by differential taxon sampling is significantly higher than by site sampling (two-tailed *t*-test $p = 0.0204$), but the inconsistency in phylogenetic distances is the opposite (two-tailed *t*-test $p = 0.00198$). The variance between the 381 global markers vs. the 30 ribosomal proteins was also stable, and the most significant, especially in phylogenetic distances (RF = 0.372 ± 0.018, TT = 0.162 ± 0.028) (Supplementary Fig. 23d). In both PCoAs, the differential choice of loci dominated the variances on axis 1 (which explains 46.70% and 92.88% variance, respectively) (Supplementary Fig. 23e, f).

Now consider differential site heterogeneity models: For each dataset, trees generated by the Gamma model and by the FreeRate model had little inconsistency (RF = 0.061 to 0.132, TT = 0.0005 to 0.0034); the PMSF tree was more discrepant from the other two (RF = 0.096 to 0.204, TT = 0.0063 to 0.0121), yet this discrepancy was lower than revealed in other comparisons (Supplementary Fig. 23c). This pattern was also indicated by hierarchical clustering (Supplementary Fig. 23g, h). In the PCoA of RF distances, trees by the three models on the same dataset form compact clusters (Supplementary Fig. 23e), whereas in the PCoA of tip distances, the PMSF trees had noticeable deviations from the Gamma and FreeRate trees (Supplementary Fig. 23f). These observations suggest that the more complex and expensive PMSF method generated highly consistent topologies, but estimated slightly less consistent phylogenetic distances, comparing to the simpler models.

Collectively, this test also reveals that with our dataset, the impact of taxon sampling on tree topology is notably larger than the impact of site sampling or model complexity, as evident in Supplementary Fig. 23e across axis 2 (which explains 19.04% variance). For example, starting from the tree using 38k randomly selected sites with 1,000 taxa (small blue square), increasing site sampling to all 192k sites (small blue circle, a.k.a. "concat.al1k" in Fig. 3) resulted in RF = 0.162, but increasing taxon sampling to all 10,575 taxa (big blue square, a.k.a. "concat.rand" in Fig. 3) resulted in RF = 0.275 (also see Supplementary Fig. 13).

Evaluation of species trees built using FastTree. While robust ML implementations like RAxML and IQ-TREE are computationally expensive and forced us to perform site downsampling, the faster alternative FastTree [16] allowed reconstruction of a CONCAT tree using all sites (192k in total). Since FastTree was used to reconstruct large-scale reference microbial phylogenies in several previous studies (e.g., [10,17]), we compared the two methods in the context of our study. In particular, we compared species trees built using either FastTree or the robust method based on the conserved sites by a series of downsampled taxa.

Our results show that FastTree and the robust method produced similar topologies given the same input data, as long as the number of taxa is large (RF = 0.111 to 0.408) (Supplementary Fig. 12a). With different input datasets, both methods yielded relatively discrepant topologies (RF = 0.438 ± 0.140 and 0.408 ± 0.139, respectively, mean and std. dev.) (Supplementary Fig. 12c, d, upper left triangles), with FastTree trees being more discrepant (paired two-tailed *t*-test $p = 0.000247$). In PCoA, input data dominantly determine the clustering pattern (PERMANOVA pseudo-$F = 7.117$, $p = 0.001$) of tree topologies, whereas method (RAxML vs. FastTree) has little effect (pseudo-$F = 0.679$, $p = 0.752$) (Supplementary Fig. 12e). When considering the estimated phylogenetic distances among taxa, we observed a mixed effect. While input dataset continued to impact the distribution of trees (pseudo-$F = 7.616$, $p = 0.001$) (Supplementary Fig. 12f), forming a clearly ascending gradient by the number of input genomes along axis 1 (which explains 62.19% variance), method also has a significant impact (pseudo-$F = 4.294$, $p = 0.025$), clearly separating paired trees of each input dataset on axis 2 (which explains 24.89% variance). The influences of input data and method on the tree distribution are comparable (RDA effect size: adjusted $R^2 = 0.512$ vs. 0.387, $p = 0.006$ and 0.004).

Therefore, despite the overall congruence in topology, there is a systematic bias between the two methods in estimating phylogenetic distances. Because our study has a strong focus on the evolutionary distances among microbial lineages, and considering that several previous studies associated FastTree with suboptimal likelihood scores [18,19] and less accurate species tree [20], we decided to favor the robust method over FastTree when reporting our results. Conducting a comprehensive comparison between FastTree and RAxML / IQ-TREE is beyond the scope of this study. Nevertheless, we want to remind readers of this difference when intepreting the robust and FastTree trees, both of which were included in our data release.

Supplementary Note 3. Evaluation and curation of NCBI taxonomy

We evaluated the NCBI taxonomy [21] with reference to the ASTRAL tree. Of all 1,980 NCBI taxonomic terms with two or more representatives in our sampled genomes, only 1,219 (61.6%) terms are monophyletic. To further quantify the divergence between taxonomy and phylogeny, we computed the classification consistency [17] and the quartet score [22] of each term. The distribution of consistency scores reveals the imperfections of the taxonomy in reflecting the phylogenetically estimated relationships (Supplementary Fig. 15a). Some large phyla were rejected consistently by different phylogenetic trees, pointing to potential inaccuracies in the taxonomy (Supplementary Fig. 15c, see also Supplementary Note 5).

Using the automated taxonomy curation algorithm tax2tree [17], we reconstructed high-confidence taxonomic lineages for individual genomes and for internal nodes of the ASTRAL tree. This process does not create or modify taxonomic terms, but edits the assignments of genomes to existing taxonomic terms. When faced with strong signal of polyphyly for a taxonomic unit, tax2tree appends a numeric suffix to the taxonomic term for each clade (e.g., Fig. 1). This analysis established the taxonomy for 873 genomes that were unclassified at one or multiple taxonomic ranks by NCBI, and modified the existing taxonomy for 1,866 genomes (Supplementary Table 3). Interestingly, at class, order and family levels, 19.36% of genomes defined as metagenome-derived received correction, while this ratio for genomes from isolates was much lower: 7.79% (one-tailed Fisher's exact test p-value = 1.03e-23). This once more implicates the challenge in metagenome-assembled genome discovery and emphasizes the need for improved quality standards for this practice [23]. Source data are provided as a Source Data file. Annotations and curations are available from our data release.

Supplementary Note 4. Comparison with GTDB taxonomy and phylogeny

GTDB is a recent phylogenomics-curated taxonomy system for bacteria and archaea [10]. We compared our work with GTDB release 86.1. Among the 10,575 taxa in our phylogenetic analysis, 9,732 (92.0%) have matches in the GTDB taxonomy, and 8,042 (76.0%) of them are present in the GTDB phylogeny. We annotated our trees using the GTDB taxonomy (e.g., Supplementary Fig. 16), and observed high overall congruence (Supplementary Fig. 15b). Among all 3,466 GTDB taxonomic units with two or more representatives, 3,403 (98.2%) are monophyletic in the ASTRAL tree. The congruence is also evident by directly comparing topologies of the GTDB phylogeny (composed of one archaea tree and one bacteria tree) and the ASTRAL tree (RF distance = 0.185) (Fig. 3a, b). However, some differences in phylum-level organization and contents were observed (Figs. 3c, d, Supplementary Figs. 3 and 15d), and the ASTRAL tree appeared to have the fewest inconsistencies compared to the CONCAT trees using the global marker gene set (Supplementary Fig. 15d). The differential inclusion of phylum-level classification units by the two works may contribute to this discrepancy. Further discussion of taxonomic units with reference to the GTDB trees and other published works is provided in Supplementary Note 5. Source data are provided as a Source Data file. We included cross translations of genome identifiers and phylogenies of the two systems, and GTDB-based taxonomic curation of our genome pool in the data release.

Supplementary Note 5. Phylogenetic relationships of major taxonomic groups

We examined the placement of multiple important high-level (phylum and above) taxonomic groups in the species trees generated in this study. The ASTRAL tree (branch support: local posterior probability, or lpp) was used as the top-priority reference for the discussion, due to its stability and high resolution in deep phylogeny as discussed above and in the main text. The two CONCAT trees built using the robust ML implementation, based on either using conserved or random sites, were used for comparison in most discussions (branch support: rapid bootstrap, or xboot).

Archaea. The 669 representatives of the domain Archaea form a distinct clade in all three species trees (lpp = 0.998 in the ASTRAL tree, xboot = 100 in both CONCAT trees). The Archaea clade is split into the four currently accepted groups, namely Asgard, TACK, Euryarchaeota and DPANN [24,25]. However, not all the groups are monophyletic, and this is particularly evident among the phylum Euryarchaeota (detailed below). Our trees do not support Asgard and TACK as sister groups (together as kingdom Proteoarchaeota, as proposed in [26]), despite the closeness of the two groups in the ASTRAL tree (detailed below).

Asgard. The recently discovered group of uncultivated archaea Asgard was considered to be close to eukaryotes and represent the archaea-to-eukaryote transition [27]. Our dataset includes eight representatives out of ten Asgard taxa from the original genome pool. Seven of them, representing the candidate phyla Lokiarchaeota (one taxon), Thorarchaeota (three taxa), and Heimdallarchaeota (three taxa), form a clade with moderate support (lpp = 0.751, xboot = 83 / 98) (a separation by "/" stands for conserved / random, same below) and reside in a relatively basal location in the Archaea lineage. In the CONCAT trees, this clade is sister to Marine Group II and III euryarchaeotes (13 and two taxa, respectively) (xboot = 49 / 85), whereas in the ASTRAL tree, it is relatively independent (see below). In contrast to [27], our only representative of the candidate phylum Odinarchaeota is placed in a distant location, sister to a clade of four members of the candidate phylum Verstraetearchaeota (lpp = 0.976, xboot = 71 / 99), which is part of the TACK group. Therefore, tax2tree curation re-assigned Odinarchaeota to the TACK group. Meanwhile, two Asgard taxa were retained in the 1,000-taxon PMSF trees: one Thorarchaeota taxon is deeply nested within the TACK clade, with candidate phylum Bathyarchaeota (one taxon) being its sister (ufboot = 99 / 100), whereas the other one, a Heimdallarchaeota taxon stands alone in a relatively basal position in the Archaea clade. We want to note the potential limitation in resolving Asgard placements due to its low availability of genome data.

TACK. The archaea TACK group (a.k.a., Proteoarchaeota) [28] was shown related to eukaryotes [26,28] and placed as a sister group to Asgard in previous analyses [25,29]. Members of the TACK group, including organisms under the phyla Crenarchaeota (169 taxa) and Thaumarchaeota (49 taxa), as well as the candidate phyla Bathyarchaeota (14 taxa), Korarchaeota (one taxon) and Verstraetearchaeota (four taxa), together with Odinarchaeota (see above), form a monophyletic clade with moderate support (lpp = 0.88) in the ASTRAL tree. This topological pattern was also found in the CONCAT trees, but with weaker support (xboot = 21 / 44). Further, in disagreement with ribosomal proteins-based results (e.g. [25]), all three trees in our study suggest that the TACK clade is sister to (lpp = 0.979, xboot = 55 / 92) the "Euryarchaeota_2 clade" (further discussed below). They together are sister to the Asgard group in the ASTRAL tree (lpp = 0.917), although this proximity is not indicated by the CONCAT trees.

Euryarchaeota. The phylum Euryarchaeota includes most of the "conventional" archaea. This group (407 taxa) appears to be polyphyletic in all three trees, which is inconsistent with [25]. In the ASTRAL tree, this phylum splits into two clades: The major clade (Euryarchaeota_1) includes genomes of the class Thermoplasmata (25 taxa), Marine Group II (12 taxa), Methanomicrobia (132 taxa), Archaeoglobi (21 taxa), Halobacteria (99 taxa), Methanococci (19 taxa) and Methanobacteria (34 taxa). The minor group (Euryarchaeota_2) (lpp = 0.752), comprising classes Thermococci (38 taxa) and Hadesarchaea (two taxa), plus the Arc I group archaea (eight taxa), forms a distinct sister cluster to the TACK group (see above). The CONCAT trees also show that Hadesarchaea and Thermococci are sister groups (xboot = 21 / 45), and they together are sister to the TACK group (see above), but the Arc I group was placed in a different location, close to classes Methanococci and Methanobacteria. For comparison, the sister relationship between Thermococci and Arc I group was also supported in [10] and [25]. Arc I group is currently classified under the euryarchaeal class Methanomicrobia, but none of our trees support this hierarchical relationship. The position of the secondary Euryarchaeota clade is also supported by the PMSF trees on 1,000 taxa, which include three Thermococci and one Hadesarchaea taxa, forming a clade sister to the 19-taxon TACK clade (ufboot = 100 / 99).

DPANN. The recently defined DPANN group of archaea [30] has five representatives in our analysis. In concordance with a recent study [25], our trees do not support the monophyly of this group. Two members of the candidate phylum Micrarchaeota form a distinct clade in all three trees. This group is basal to the entire Archaea clade in the ASTRAL tree (lpp = 0.998). The candidate phyla Diapherotrites and Woesearchaeota each have one representative, and they form a clade with two unclassified archaea: GW2011_AR10 and GW2011_AR15. This clade is sister to the Micrarchaeota clade in the CONCAT trees with moderate support (xboot = 67 / 63), but the two clades are not adjacent in the ASTRAL tree.

In addition, the five representatives of the candidate order Altiarchaeales, which was recovered to be within the DPANN clade in previous studies [25,29], form a clade nested within a big clade mainly composed of the orders Methanococcales and Methanobacteriales, and this clade is distant from the DPANN clades.

It should be noted that the taxon sampling of the DPANN group is sparse in this study compared to previous studies that focused on newly discovered organisms (e.g., [25]). This is mainly because the DPANN genomes have low numbers of detectable marker genes (67.81 ± 31.19, mean and std. dev.). As a consequence, only five out of 57 available genomes were selected using our genome subsampling protocol. (But see Supplementary Note 7 for discussion of expanded DPANN sampling.) The proposed importance of DPANN in understanding the basal diversification of Archaea [31] calls for future improvements of our marker gene set.

CPR. The candidate phyla radiation (CPR) [32] comprises a large proportion of the bacterial diversity. Our trees include 1,454 CPR genomes, which form a single lineage with full support in all trees. Consistent with [25], the candidate phylum Wirthbacteria (one genome) is basal to the entire CPR clade, with full support in all trees. A clade comprised of the candidate phyla Peregrinibacteria [33] (60 taxa) and Abawacabacteria [11,34] (one taxon) as sister groups (full support in all trees) was recovered as the second basal group in the CONCAT trees (full support) and as an early branching group, though not second basal, in the ASTRAL tree (full support). This pattern was not revealed in [25]. Most CPR taxa are grouped under two highly supported clades representing the superphyla Microgenomates [30] (a.k.a. OD1, 423 taxa) (lpp = 0.913, xboot = 97 / 96) and Parcubacteria [30] (a.k.a. OP11, 846 taxa) (lpp = 1.0, xboot = 99 / 100), respectively. The two clades are relatively derived and are not immediate sister groups. Thus the previous proposal of the superphylum Patescibacteria, comprised of Microgenomates, Parcubacteria, and the candidate phylum Gracilibacteria [30], is not supported [25]. Our sampling did not include any of the five genomes of Gracilibacteria, though, since they did not pass the quality filters. The candidate phylum Doudnabacteria [34] (19 taxa), was placed within the Parcubacteria clade in the ASTRAL tree and the random CONCAT tree, with weak support (lpp = 0.621, xboot = 60), a pattern consistent with previous work based on ribosomal proteins [34], but was basal to the entire Parcubacteria clade (xboot = 100) in the conserved CONCAT tree. Overall, the relationships among major CPR candidate phyla were much more consistently resolved compared to phyla under non-CPR Bacteria (see below) (Supplementary Fig. 3).

Non-CPR Bacteria *(abbreviated as "**ncBacteria**" in this section)*. They form a monophyletic group in all trees based on global marker genes. This clade is highly supported in the ASTRAL tree (lpp = 0.958) and in the random CONCAT tree (xboot = 95) but less so in the conserved CONCAT tree (xboot as low

as 29) (Fig. 3c). The CONCAT method struggled to resolve the relationships of the early branching ncbacterial clades, leaving poorly supported branches that were collapsed into polytomies in Supplementary Figs. 5 and 6. However, the ASTRAL tree provides remarkably higher resolution with moderate-to-high support of those basal relationships (Supplementary Figs. 4 and 9). In this tree, a clade is basal to the whole ncBacteria clade (full support), comprised of the phyla Thermotogae (35 taxa), Dictyoglomi (two taxa), and Caldiserica (two taxa), plus Firmicutes genera *Coprothermobacter* (three taxa) and *Thermodesulfobium* (one taxon). All of those taxonomic groups are featured by their thermophilic and anaerobic behavior. The basal placement of Thermotogae and other rooted groups within ncBacteria obviously support the hypothesis of an origin and early diversification of ncbacteria as (hyper)thermophilic anaerobes [35,36].

Terrabacteria vs. "Hydrobacteria". Post the branching off of the (hyper)thermophilic bacteria clade in the ASTRAL tree, the ncbacteria clade split into two major clades (lpp = 0.988). One (3,708 taxa) is mainly composed of taxa under the widely accepted term Terrabacteria, the largest group of ncbacteria that have shared adaptations to the terrestrial lifestyle [37]. Specifically, it contains the five originally suggested terribacterial phyla: Actinobacteria, Firmicutes (including Tenericutes and Synergistetes), Cyanobacteria, Chloroflexi, and Deinococcus-Thermus [37], plus the more recently defined phylum Armatimonadetes (previously known as OP10) [38]. This clustering pattern was not revealed in [25] and [10]. The CONCAT trees inferred in this study also indicated mixed support/rejection for this clade (Fig. 3c, d). Multiple candidate phyla reside within the Terrabacteria clade, which help to further define their classification status. The other major clade (4,701 taxa), overlapping with the less commonly used term "Hydrobacteria" suggested by the same authors [37], contains the remaining ncbacterial diversity. The deep phylogeny of the Hydrobacteria clade reveals an interesting pattern of rapid diversification.

Aquificae vs. Thermotogae. The hyperthermophiles Aquificae and Thermotogae were conventionally determined as closely related groups (e.g., [25]) and together occupy the basal position of the ncbacteria clade [37,39]. Our work, however, is consistent with that of [30] and found a clade containing the phylum Aquificae (17 taxa) and the candidate phylum Calescamantes [30] (a.k.a. EM19, seven taxa) (lpp = 1.0, xboot = 60 / 86), sister to a clade mainly comprised of class Epsilonproteobacteria (lpp = 0.687, xboot = 90 / 85) and distant from Thermotogae. Similar findings were obtained in some earlier comparative genome analyses of these groups [40,41], while another study found no distinctive evolutionary relationship between the two groups [42], despite many members of them sharing similar ecology and physiology.

Synergistetes. The phylum Synergistetes (29 taxa, excluding one mis-classified taxon Synergistes sp. Zagget9) form a monophyletic clade in all three trees with full support and is proximate to several

candidate phyla in the ASTRAL tree (lpp = 0.787). However, in the CONCAT trees, the Synergistetes clade is paraphyletic to the thermophilic bacteria clade (see above) with low support (xboot = 32 / 27). Previous studies suggested a close relationship between Synergistetes and Firmicutes, but had uncertainty in the placement of the Synergistetes clade relative to the latter [43]. Our trees suggest that Synergistetes is not an ingroup of Firmicutes, consistent with [44] but in contrast to [25].

Firmicutes/Tenericutes/Fusobacteria. The phylum Firmicutes has been widely reported to be a polyphyletic group, primarily because of the unstable positions of Tenericutes and/or Fusobacteria [11,44,45]. In our analysis, the 66 taxa of the phylum Tenericutes are nested within the Firmicutes clade in all three trees. However, this pattern is only credible in the ASTRAL tree (lpp = 1.0), whereas in the CONCAT trees, the relevant branches have low support (xboot < 50). The Tenericutes taxa are para- or polyphyletic, mainly forming two clades, in close proximity to the Firmicutes class Erysipelotrichia (50 taxa). The taxa of the two groups cannot be clearly separated. It is remarkable that the Tenericutes clade has very long branch lengths compared to the remaining Firmicutes and the entire tree. These results show the non-determinacy of the hierarchical relationships between the two phyla. Unlike Tenericutes, the 36 taxa of the phylum Fusobacteria form a distinct cluster within the "Hydrobacteria" group in the ASTRAL tree (lpp = 0.75), which is consistent with [44]. However, the CONCAT trees show that the Fusobacteria clade is nested within Firmicutes, sistering the Tenericutes-Erysipelotrichia clade, with low support (xboot = 10 / 50). The instability of the class Clostridia, another Firmicutes group, has previously been noted [46–48], mainly as a result of misclassification of several species within the genus *Clostridium* [49]. In the ASTRAL tree, almost all the clades for class Clostridia (Supplementary Fig. 4) have high support (lpp > 0.98), indicating that this tree can be an effective reference for resolving the problem of the classification of Clostridia.

Actinobacteria. Several orders in the phylum Actinobacteria, particularly Micrococcales and Pseudonocardiales, are widely known to be polyphyletic, and few efforts to rectify this problem using a combination of phylogenetic markers have been reported [50]. In our study, the phylum Actinobacteria was found as a monophyletic clade in the ASTRAL tree (lpp = 0.986) and the random CONCAT tree (xboot = 88). This finding is consistent with several previous studies [2510]. Recently, Parks, et al., proposed to downgrade Nitriliruptoria to an order within the class Actinobacteria [10]. In our trees, however, the class Nitriliruptoria (one taxon) forms a distinct branch, well separated from the classes Actinobacteria and Acidimicrobiia.

Cyanobacteria/Melainabacteria. The candidate phylum Melainabacteria (17 taxa) is a recently discovered group of bacteria that are closely related to the phylum Cyanobacteria (a.k.a.

Oxyphotobacteria, 295 taxa) but that lack the capability of photosynthesis [10]. Our trees support the members of Melainabacteria, plus 11 underclassified, metagenome-assembled genomes, as a fully supported monophyletic group, sister to the Cyanobacteria clade (lpp = 1.0, xboot = 100 / 98), which is also monophyletic (with full support). In contrast to [11,25], our analysis did not recover it as a basal group to non-CPR Bacteria.

Chloroflexi. Members in the phylum Chloroflexi are model organisms for investigating a number of hypotheses related to the early evolution of photosynthetic life [51]. In all three trees, the 100 taxa of the phylum Chloroflexi form a single lineage (lpp = 0.83, xboot = 94 / 100). Our finding also suggests that the Chloroflexi group diverged during a similar period of time as the Cyanobacteria/Melainabacteria group (Supplementary Fig. 25, see Supplementary Note 6 for details), which is consistent with a recent study [51]. Furthermore, in this phylum, the order Chloroflexales is considered as the main phototrophic lineage that performed anoxygenic photosynthesis with a divergence time later than that of Cyanobacteria/Melainabacteria group. This observation does not support the hypothesis that anoxygenic photosynthesis preceded the development of oxygenic photosynthesis [52], in congruence with [51]. While the origin of photosynthetic life on the basis of the analysis of extant lineages is still unclear, the problem of undiscovered or extinct lineages further limits our understanding of evolution of phototrophy.

Spirochaetes. The basal position of the "Hydrobacteria" clade is occupied by four monophyletic lineages, represented by two cultured phyla – Fusobacteria (36 taxa) and Spirochaetes (135 taxa) – and two candidate phyla – Lindowbacteria (one taxon) and Aeriogibetes (three taxa). The evolutionary lineage of the phylum Spirochaetes in the ASTRAL tree and the random CONCAT tree is more consistent with [10], but contradictory to [25], which placed the phylum closer to the Proteobacteria. Further, in contrast to the view of Yarza, *et al.* [53], our trees do not support the classification of the phylum Spirochaetes into five lineages at the class level, but rather should be determined to have triphyletic subgroups (lpp = 0.99): one containing the main order Spirochaetales (98 taxa), the second containing the order Brachyspirales (9 taxa), and the third containing the family *Leptospiraceae* of the order Leptospirales (27 taxa). The 43 taxa of the Spirochaetales family *Borreliaceae* form a shallow clade with a long stem, implicating a recent radiation.

PVC and FCB. The PVC and FCB superphyla groups form two monophyletic clades in all trees of life reported so far [10,11,37]. The topology of our trees also supports the divergence patterns reported earlier but provides a more robust position for an associated cluster of cultured and candidate phyla. Within this cluster, the phylum Gemmatimonadetes and the candidate phyla Glassbacteria, Eisenbacteria,

Edwardsbacteria, Cloacimonates, Hyd24-12, and WOR-3 are closely related FCB (lpp = 0.585), the candidate phyla Hydrogenedentes, Omnitrophica, Desantisbacteria, and Firestonsebacteria are closely related to PVC (lpp = 0.99), and the rest, including the phylum Elusimicrobia and the candidate phyla Poribacteria and Coatesbacteria, form the root (lpp = 1.0). While the robustness of our tree might be related to the number of selected marker proteins and/or the number of genomes used, the diversification of the different associated groups clearly suggests an evolutionary pattern for such divergence. For example, members of the phylum Gemmatimonadates can undergo both aerobic and anaerobic respiration, which enable them to adapt to an arid environment [54], while members of the phyla Chlorobi and Fibrobacteres are usually found under more strict anaerobic conditions [55].

Proteobacteria. The phylum Proteobacteria is the largest bacterial lineage of the rank, with 2,975 taxa in this study. The main subgroups of this phylum, particularly the classes Alphaproteobacteria, Betaproteobacteria, and Gammaproteobacteria are monophyletic, with the latter two sharing the same root. The class Epsilonproteobacteria (110 taxa) forms a sister clade (with full support) to a small clade comprised of deltaproteobacterial genera Desulfurella (one taxon) and Hippea (four taxa), then to the Aquificae-Calescamantes clade (see above). This pattern is consistent in all three trees, and is consistent with [10] but in disagreement with [11]. Our finding is also significant in the evolutionary point of view, as multiple Epsilonproteobacteria, particularly those isolated from deep-sea hydrothermal vents, meet their energy requirements through chemolithoautotrophy [56], a physiological condition related to the phylum Aquificae. The Epsilonproteobacteria-Aquificae clade is closely related to the class Deltaproteobacteria, which itself appears to be paraphyletic, with several other phyla such as Nitrospinae, Nitrospirae, and Thermodesulfobacteria nested within it. Parks, et al., proposed to upgrade Epsilonproteobacteria and Deltaproteobacteria to a new phylum [10]. The distinctive placement of these two classes in our trees is roughly in concert with this proposal, though a more definitive study will be necessary.

Supplementary Note 6. Compatibility with geological timeline

We performed a series of divergence time estimation analyses to further demonstrate the efficacy of the 381 global marker genes in assessing the microbial evolutionary history. As revealed in Fig. 4, the evolutionary distance between Bacteria and Archaea was significantly shorter by using the global markers than by using the ribosomal proteins. Therefore, we focused on testing whether this observation is realistic, by projecting the species trees to the geologic timeline.

Maximum likelihood under a universal clock. Dating a phylogenetic tree of microbes has long been a challenge since few to no reliable fossil records are available to calibrate the tree [57,58]. We performed a literature search and selected one calibration point that is among the most confident ones within bacteria and archaea:

Calibration 1: the origin of photosynthetic cyanobacteria. Specifically, it is the node that splits phylum Cyanobacteria and candidate phylum Melainabacteria, a recently discovered group of non-photosynthetic bacteria that are closely related to Cyanobacteria [59]. In our tree, the two sister clades have 295 and 28 taxa, respectively, with strong branch supports (further discussed in Supplementary Note 5). It is widely accepted that the rise of oxygen in the Earth's atmosphere was a direct consequence of the evolution of photosynthetic bacteria, specifically, Cyanobacteria [60]. Recently, the Great Oxygenation Event (GOE) was precisely dated to 2.33 Ga (billion years ago) based on sulfur isotope signals [61]. In an independent study, the Cyanobacteria/Melainabacteria split was further estimated to be 2.5-2.6 Ga, using four calibrations based on well-accepted plant fossil records [62]. This range closely predates the GOE, indicating strong consistency with the aforementioned hypothesis of oxygenic photosynthesis evolution. Therefore, we adopted this range to constrain the Cyanobacteria/Melainabacteria split in the species trees.

We started with this single calibration, a simple assumption of one universal clock, and a maximum likelihood method which can be applied to the entire dataset. The age of LUCA was estimated to be 4.1-4.2 Ga (in Hadean) by conserved sites, or 3.6-3.7 Ga (in Eoarchean) by random sites (Supplementary Table 8). Either estimate is within the range consistent with the latest microfossil evidence [63] and in-silico estimations of life origination [64]. The split between CPR and non-CPR Bacteria took place 3.9 Ga (conserved) or 3.5-3.6 Ga (random). No later than 3.2 Ga (end Paleoarchean), all three major clades began to diverge (Fig. 6, Supplementary Fig. 25). In contrast, using the ribosomal proteins, we obtained a very early estimate of the age of LUCA: 7 Ga (Supplementary Table 8), which is inconsistent with the well-established age of the planet [65], whereas the divergence times of more derived lineages roughly agree with those by the global markers.

Impact of method, site sampling, site model and root placement. Comparative analyses suggest that the estimated ages were mainly influenced by gene and site sampling, whereas the impact of the tree-building method was minimal (Supplementary Table 8). Considering the potential impact of root placement on the analysis, we moved the root from the midpoint of the Archaea-Bacteria branch to the first and third quarters, and obtained consistent results (Supplementary Table 8). We then examined the impact of site model (PMSF vs. Gamma) on the 1,000-taxon trees (Supplementary Table 8). For global markers, the difference is minimal. The age of LUCA estimated by random sites agree with the full tree (3.7 Ga), while that by conserved sites is slightly earlier (4.5 Ga), likely an impact of taxon downsampling (discussed above). For ribosomal proteins, the age of LUCA was further pushed to 9.2 Ga by PMSF from 7.5 Ga by Gamma.

Alternative calibrations. We tested the compatibility of multiple other calibration points and ranges with the photosynthetic cyanobacteria-based estimation, although these hypotheses are usually controversial or less precise (with lower bound only).

<u>*Calibrations 2 and 3*</u>: The origin of photosynthetic eukaryotes. The widely adopted endosymbiotic theory [66] suggests that eukaryotic organelles originated from symbiotic prokaryotes. The earliest fossil of photosynthetic eukaryote with relatively evident morphological characteristics, *Bangiomorpha pubescens* (a red alga), was recently precisely dated to 1,047 +13/-17 Ma [67]. Therefore we used the age 1.03 Ga to define the lower bounds of postulated bacterial and archaeal lineages from which organelles evolved through endosymbiosis. Specifically, it is commonly agreed that plastids evolved from cyanobacteria [68], although the specific cyanobacterial lineage is under debate (e.g., [69,70]). Therefore we placed this calibration at crown Cyanobacteria.

On the other hand, it has been long suggested that mitochondria evolved from an alphaproteobacterial lineage, most likely Rickettsiales [71]. However, a recently study placed the mitochondrial origin at a proteobacterial lineage that branched off before the diversification of alphaproteobacteria [72]. We tested both theories, by placing the calibration at either crown Alphaproteobacteria (which has 893 taxa) or the split between Alphaproteobacteria and other proteobacteria (mostly beta- and gammaproteobacteria).

<u>*Calibration 4*</u>: The origin of akinetes-forming cyanobacteria. Several groups of extant cyanobacteria under families Nostocaceae and Stigonemataceae (both belong to order Nostocales) have the capability of forming environmental stress-resistant cells: akinetes [73]. Fossil akinetes (referred to as *Archaeoellipsoides*) have been recorded from a wide time period, most frequently between 1.4 Ga and 1.65 Ga [73]. The relationship between those records and modern Nostocales species remains controversial [74]. Despite being a frequently used calibration (e.g, [75]), some authors chose not to adopt it considering

the controversy (e.g., [76]), and some found it to strongly impact age estimation (e.g., [77]). In our tree, order Nostocales (54 taxa) is monophyletic and nested within the Oscillatoriales clade, which is roughly consistent with [73]. We sequentially constrained the origin of the Nostocales clade with four representative ages of fossil akinetes: 1.2 Ga [78], 1.5 Ga [79], 1.9 Ga [80] and 2.1 Ga [81].

Calibration 5: The origin of aphid-*Buchnera* symbiosis. *Buchnera aphidicola* is the primary obligate symbiont of aphids (Aphidoidea) [82]. This close relationship was estimated to originate from 84-164 Ma [83], as evident by the radiation of fossil aphids and the implication from a geological thermal shift. This estimate is roughly consistent with more recent studies on larger scopes (e.g., [84]). Some authors (e.g., [75]) applied this calibration to the split between *Buchnera* and *Wigglesworthia* (obligate symbionts of a different host: tsetse fly). In our robust taxon sampling, a *Candidatus* Tachikawaea gelatinosa [85] taxon is slightly more closely related than *Wigglesworthia* to the eight-taxon *Buchnera* clade, however considering that it has not been rigorously studied, we still placed the calibration at the *Buchnera*/*Wigglesworthia* split, and we used either 84 Ma or 164 Ma to define the lower bound of it.

Our results (Supplementary Table 9) show that the estimated ages of LUCA and non-CPR Bacteria remained largely consistent when either or both the photosynthetic eukaryotes calibrations and the aphid-*Buchnera* symbiosis calibration, with all their variants, were included in addition to the photosynthetic cyanobacteria calibration. However when the akinetes-forming cyanobacteria calibration (with any of the four variants) was introduced, it strongly pushed the estimations backward to an unlikely range. These results provide new information for paleobiological discussions.

Bayesian inference with alternative models. To validate and further strengthen the findings from maximum likelihood and the simple assumption of one clock, we analyzed the data using the more robust Bayesian inference method, with alternative clock models (strict or relaxed). The computational challenge forced us to downsample data to 5,000 sites by 100 taxa (the impact of downsampling was discussed in Supplementary Note 2), the latter of which was selected to maximize the representation of deep phylogeny, but also to include sufficient sampling around the calibration point. Specifically, seven Cyanobacteria and three Melainabacteria taxa were included.

We tested two alternative prior distributions of time constraints. First ("narrow"), we adopted the estimated 2.5-2.6 Ga range (see above), and specified a normal distribution with mean = 2.55 and std. dev. = 0.025, so that 95% probability falls with this range. Next, we explored paleogeological evidence and alternative theories of cyanobacteria evolution, and specified a more relaxed constraint ("wide"):

Calibration 1 rev. Robust isotopic records have been found indicative of free oxygen in ocean or atmosphere around 3.0 Ga [86–88], while the earliest putative evidence was dated to 3.23 Ga [89]. The

connection between early signs of oxygen with photosynthetic cyanobacteria has long been suggested [90], although the relationships among early oxygen, phototrophy, filamentous microfossils and ancestral cyanobacteria remain much debated, and usually questioned by recent studies [91–94]. Here we adopt a treatment analogous to Shih et al. [62], by placing a soft upper bound at 3.0 Ga.

Accordingly, we specified a lognormal distribution, with offset = 2.33, which is the date of GOE (see above), mean = 0.22, so that mean + offset = 2.55, which is in the midpoint of the estimated range (see above), and std. dev. = 0.268, so that 95% probability falls before 3.0 Ga, when free oxygen was evident (see above) (Plus, 97.5% probability falls before 3.23 Ga, see above).

Our results (Supplementary Table 10, Supplementary Fig. 26) show that the estimated ages of LUCA were close between alternative clock models (strict vs. relaxed) and time constraints (narrow vs. wide), and supported the results based on on full-scale trees. We also calculated the coefficient of variation (C.V.) of clock rate under the relaxed clock model, a measurement of how "clock-like" the data are [95]. The C.V. by using the global markers (despite randomly downsampled to 5,000 sites) was ~0.175, showing a modest deviation from a universal clock. Meanwhile, the C.V. by using the 30 ribosomal proteins was ~0.254, suggesting a larger violation.

Taken together, we demonstrated that the microbial evolution dated using the 381 global marker genes and our species tree correspond well with the current paleobiological and geological evidence and theories. In contrast, the ribosomal proteins, which tend to overestimate the evolutionary distance between Bacteria and Archaea (see main text), consistently resulted in LUCA age estimates far older than Earth formation. This implicates a strongly accelerated evolution in the ribosomal proteins during the Bacteria-Archaea split. Therefore, we suggest that future researchers take caution when attempting domain-level divergence time estimations using a handful of "core" genes such as the ribosomal proteins. Although more comprehensive studies will be required, our analysis has indicated value of using the global marker genes for more accurate divergence time analysis. Nevertheless, we do not recommend treating our result (Supplementary Figs. 25 and 26) as a precise time table for microbial evolution, considering the simplicity of method and the sparsity of reliable and accurate calibrations.

Supplementary Note 7. Phylogenetic analysis with latest genome availability

We collected bacterial and archaeal genomes from NCBI RefSeq and GenBank on May 23, 2019. From this updated genome pool, we examined phylum-level classification units as defined by the latest NCBI taxonomy (released on June 1, 2019, which is after RefSeq 94) and GTDB taxonomy (version 4, released on June 19, 2019, indexed to RefSeq 89). For phyla that are absent, or represented by less than three genomes in the current set of 10,575 genomes, we selected new genomes with highest number of marker genes (must be no less than 100) to make the sampling up to three within each phylum. Genomes with CheckM contamination score larger than or equal to 5% were excluded. This process added 187 new genomes, representing an added or updated set of 52 NCBI phyla and 66 GTDB phyla.

We performed phylogenetic reconstruction with the 187 genomes added to the dataset, totaling 10,762 genomes. The procedures are largely consistent with the ASTRAL and CONCAT methods as described above, with several modifications to reduce computational expense (see Methods). Importantly, the same set of 381 marker genes and the same set of up to 100 most conserved or randomly selected sites per gene were used, granting comparability with the main analysis.

The resulting phylogenetic trees are highly consistent with the main results. In the ASTRAL tree we observed the highest consistency with the main ASTRAL tree (RF = 0.035) (Supplementary Fig. 27), while the two CONCAT trees using either most conserved or randomly selected sites also show high consistency with the corresponding CONCAT trees in the main analysis (RF = 0.122 and 0.099, respectively). All three trees support the separation of Archaea, CPR and non-CPR Bacteria. The domain-level evolutionary distances are also highly close to the main results (Supplementary Table 11). Therefore, our main findings hold with the up-to-date genome data.

The newly added genomes provide several insights. First, in the ASTRAL tree a new clade is placed at the base of the non-CPR Bacteria clade, consisted of three genomes classified as phylum UBP7 in GTDB. This placement is consistent with Parks *et al.* [10] in that it is the most CPR-proximal clade. However the CONCAT trees lack resolution at the base of the non-CPR Bacteria clade to reveal this relationship (see also Supplementary Figs. 3 and 8). Second, the previously underrepresented DPANN group (five taxa) was expanded, and revealed the same phylogenetic pattern (see Supplementary Note 5). Specifically, the main clade residing at the base of the Archaea clade now contains six DPANN genomes and two unclassified genomes, and the secondary Micrarchaeota clade now has four taxa and is still separated from the main clade.

Supplementary Figures

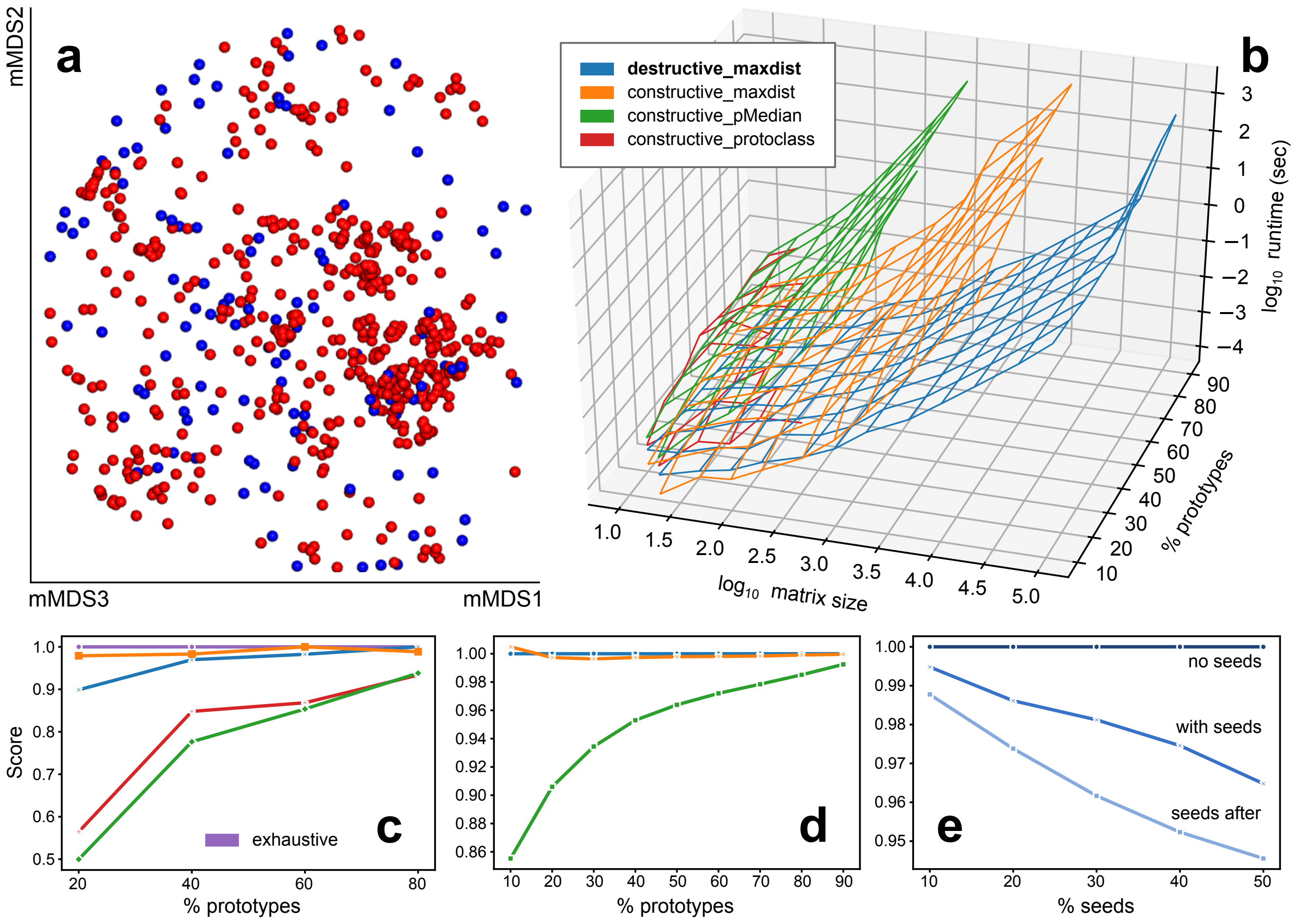

Supplementary Fig. 1. Prototype selection for maximizing biodiversity included by fixed number of genomes. a. Visual effect of the final result of the genome subsampling workflow: metric MDS plot of the genome distance matrix, showing selected genomes (blue) vs. remaining ones (red). Despite that the distribution of genomes is highly uneven, this statistical approach delivered an evenly-distributed subset of genomes. Considering computational challenge and visualization purpose, this plot shows 1,000 genomes randomly sampled from all 86,200 genomes, of which, 112 belong to the 10,575 genomes selected for phylogenetic reconstruction. **b**. Runtime comparison of four alternative heuristics to solve the prototype selection problem (detailed in Supplementary Note 1), of which destructive_maxdist was eventually used to subsample genomes in this work. The *x*-axis is the size of the randomly generated distance matrix: $n = |D|$, the *y*-axis is the amount of prototypes to select: k, given in ratios of n, and the *z*-axis is runtime in seconds. Execution time was limited to one hour at most. The runtimes for constructive_protoclass were trimmed off early because it could not find solutions for the given k with large datasets. **c**. Score (sum of pairwise distances among selected data points) normalized

by that of the exact best solution (as computed using exhaustive search) vs. ratio of prototypes, on a small distance matrix with $n = 25$. **d**. Score normalized by that of destructive_maxdist vs. ratio of prototypes, on a moderate-size distance matrix with $n = 1000$. **e**. Scores of destructive_maxdist at $n = 1000$ and $k = 20\%$, when randomly selected seeds (*r*, given in ratios of *k*) were provided ("with seeds"), as normalized to that when no seeds were specified ("no seeds"). The third curve, "seeds after" was computed when the same set of seeds were removed from the distance matrix prior to prototype selection, and then added back to the selection after the operation. In another word, it bypassed the "seeds" function implemented in the destructive_maxdist algorithm. Source data are provided as a Source Data file.

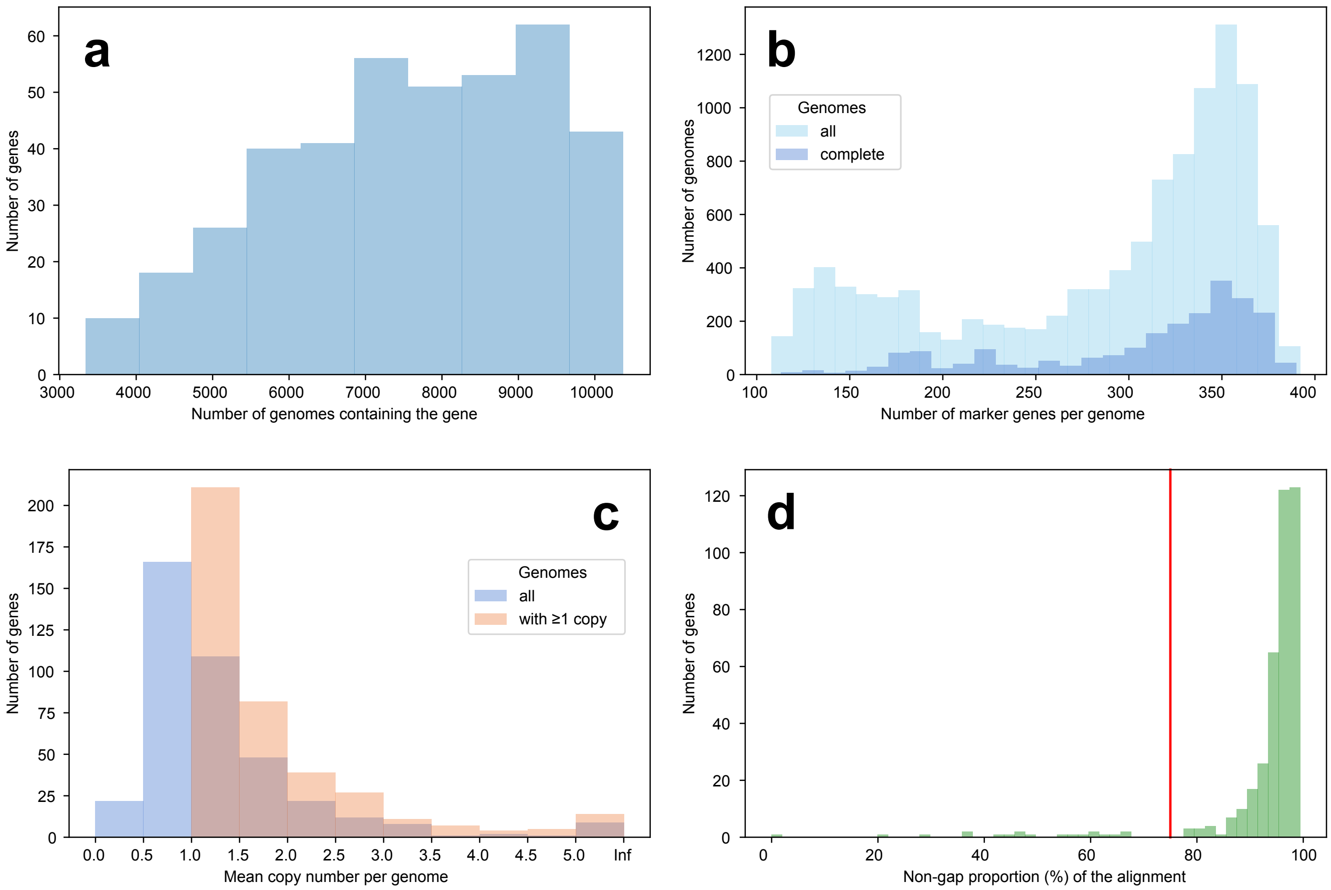

Supplementary Fig. 2. Statistics the 400 marker genes in the 10,575 sampled genomes. a. Distribution of the number of genomes where individual genes were identified. **b**. Distribution of the number of identified marker genes per genome. "Complete" is a subset of all genomes, which were marked as "Complete Genome" or "Chromosome" by NCBI. **c**. Distribution of mean copy number per genome of each marker gene. The "copy number" is the count of USEARCH hits at an E-value threshold of 1e-40 during the PhyloPhlAn marker gene discovery. **d**. Distribution of the proportion of non-gap sites in the multiple sequence alignments of individual marker genes. The red vertical line indicates the threshold we chose based on observing this distribution pattern. Nineteen marker genes below this threshold were dropped, leaving 381 for the subsequent phylogenetic analysis. Source data are provided as a Source Data file.

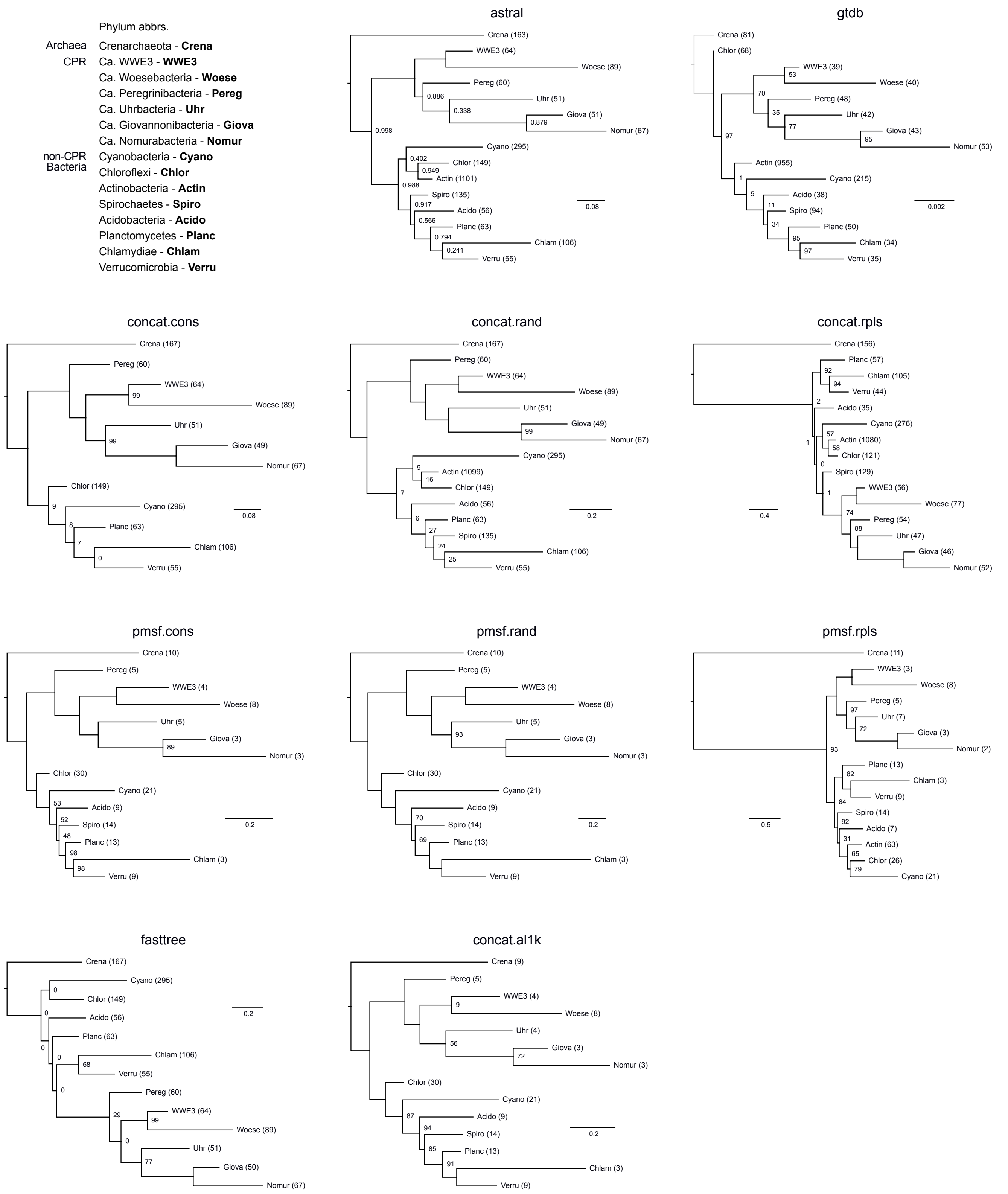

Supplementary Fig. 3. Phylum-level relationships revealed by multiple species trees. Nine species trees reconstructed in this work plus the previously published GTDB release 86.1 tree are displayed (see Fig. 3). The phyla were selected from the tax2tree-curated NCBI phyla based on the ASTRAL tree.

Fifteen most specious phyla which had no significant violation of monophyly according to tax2tree were selected. For each of the other nine trees, the same 15 phyla were selected, but any of them was omitted if it violated monophyly based on the tree-specific tax2tree curation. Only the LCA of each phylum is shown, while all descending branches were pruned. Numbers in parentheses represent the number of descendants under each clade. Node labels represent branch support values (see Fig. 3). Nodes without labels were fully supported. The branch length scales are in the unit of number of substitutions per site. For display purpose, the branch lengths of the ASTRAL tree were estimated using conserved sites (same as in Fig. 1). Also for display purpose, the GTDB Archaea tree and Bacteria tree were artificially connected by a grey line which bears no information of topology or branch length. Source data are provided as a Source Data file.

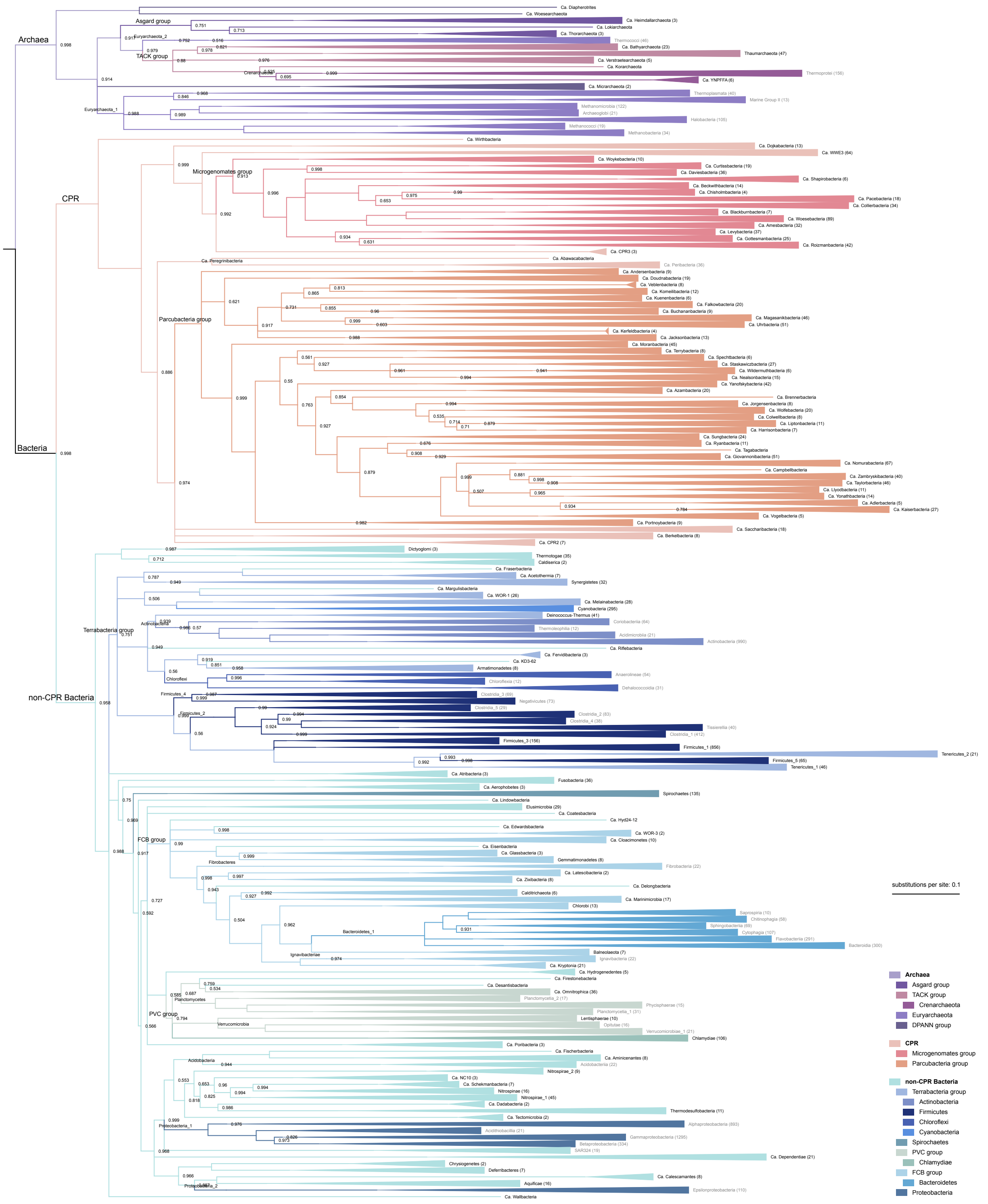

Supplementary Fig. 4. The ASTRAL summary tree rendered in rectangular layout, collapsed to class level. The displayed features are consistent with Fig. 1. The triangles represent collapsed clades, with length equal to the longest branch in the clade. Node labels represent local posterior probability (lpp) of the corresponding branch. Labels are omitted at fully-supported (lpp = 1.0) branches. Source data are provided as a Source Data file.

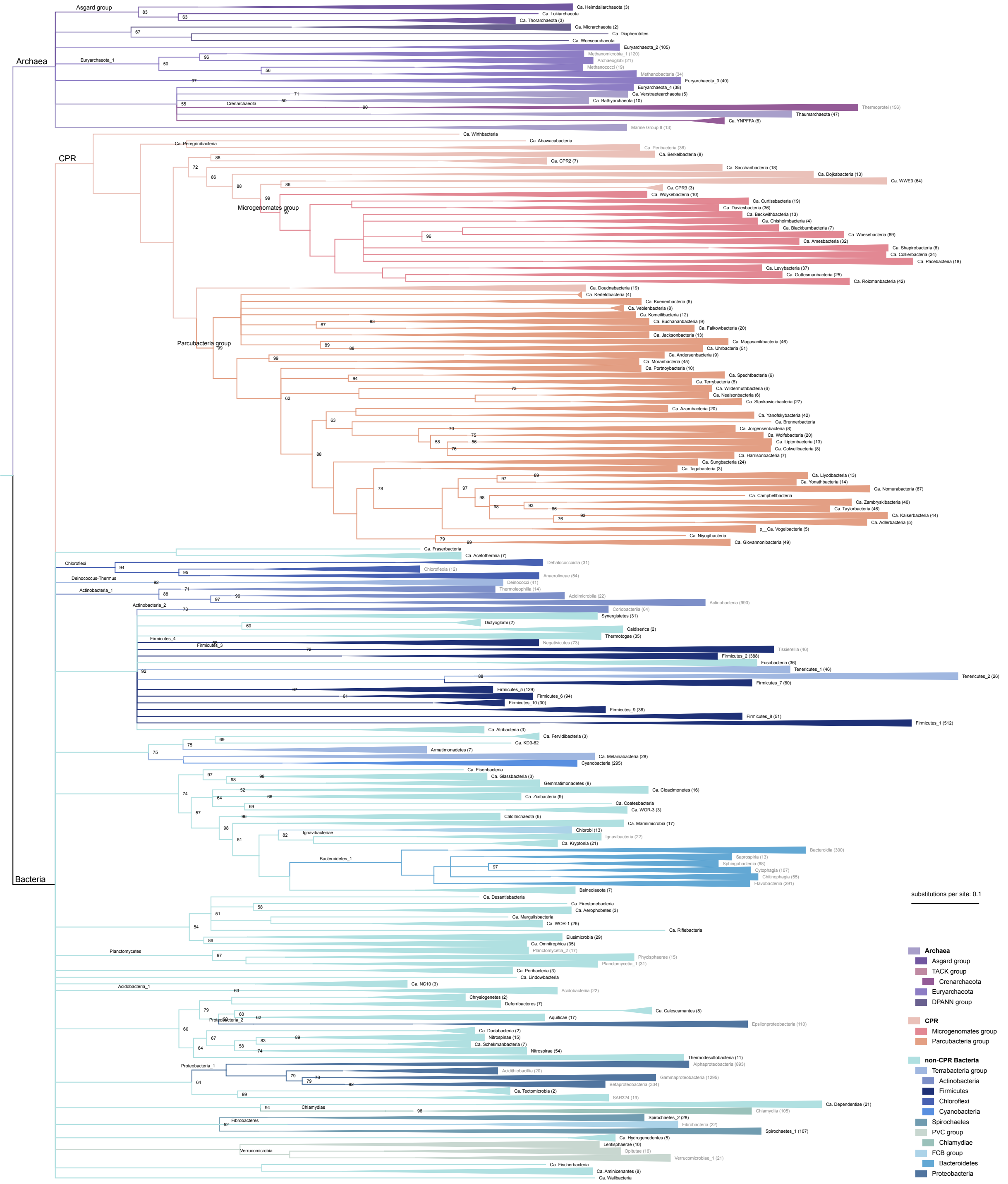

Supplementary Fig. 5. The RAxML concatenation tree based on the 100 most conserved sites per gene, rendered in rectangular layout, collapsed to class level. Node labels represent rapid bootstrap support values (out of 100). Labels are omitted at fully-supported branches. See Supplementary Fig. 4's caption. Source data are provided as a Source Data file.

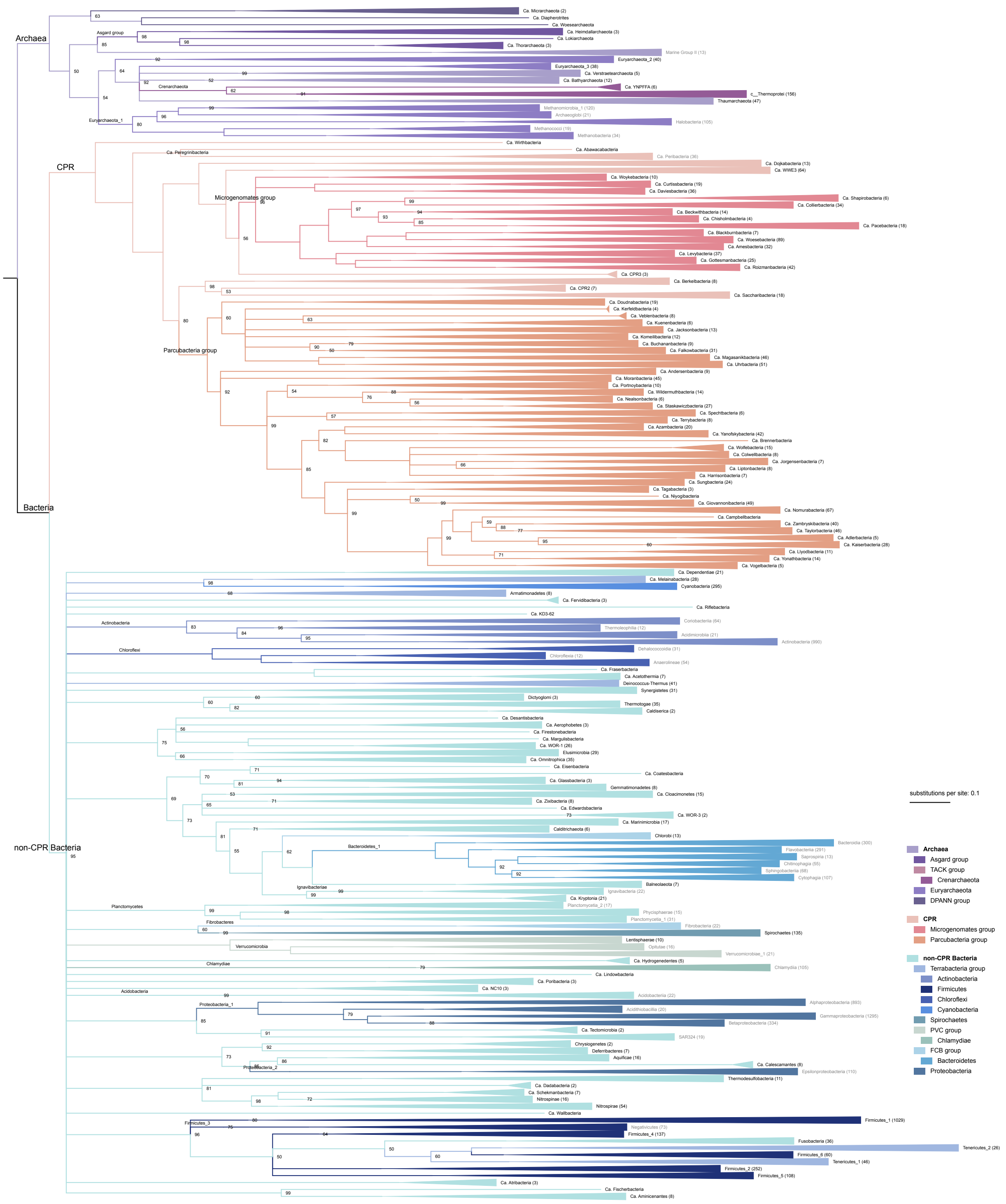

Supplementary Fig. 6. The RAxML concatenation tree based on the 100 randomly selected sites per gene, rendered in rectangular layout, collapsed to class level. Node labels represent rapid bootstrap support values (out of 100). Labels are omitted at fully-supported branches. See Supplementary Fig. 4's caption. Source data are provided as a Source Data file.

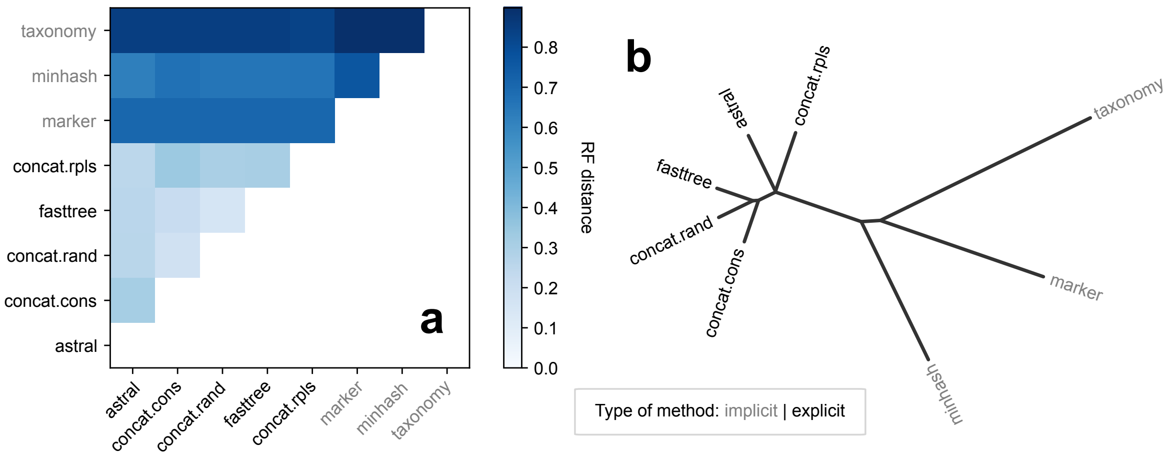

Supplementary Fig. 7. Comparison of topologies of species trees built using explicit and implicit methods. a. Heatmap of RF distance matrix. **b**. Hierarchical clustering of RF distance matrix. “taxonomy”: NCBI taxonomy hierarchy; “minhash”: neighbor-joining (NJ) tree based on the Jaccard distance matrix calculated using the MinHash signature of genomes; “marker”: NJ tree based on the Jaccard distance matrix calculated using the presence / absence of the 400 marker genes in genomes; “concat”: phylogenetic trees built using the conventional gene alignment concatenation strategy; “astral”: phylogenetic tree built using the gene tree summary method ASTRAL; “cons”: 100 most conserved amino acid sites per each of the 381 marker genes; “rand”: 100 randomly selected sites per gene; “fasttree”: all sites, but tree was inferred using FastTree (the other concat trees were inferred using RAxML); “rpls”: 30 ribosomal proteins instead of the 381 marker genes. Source data are provided as a Source Data file.

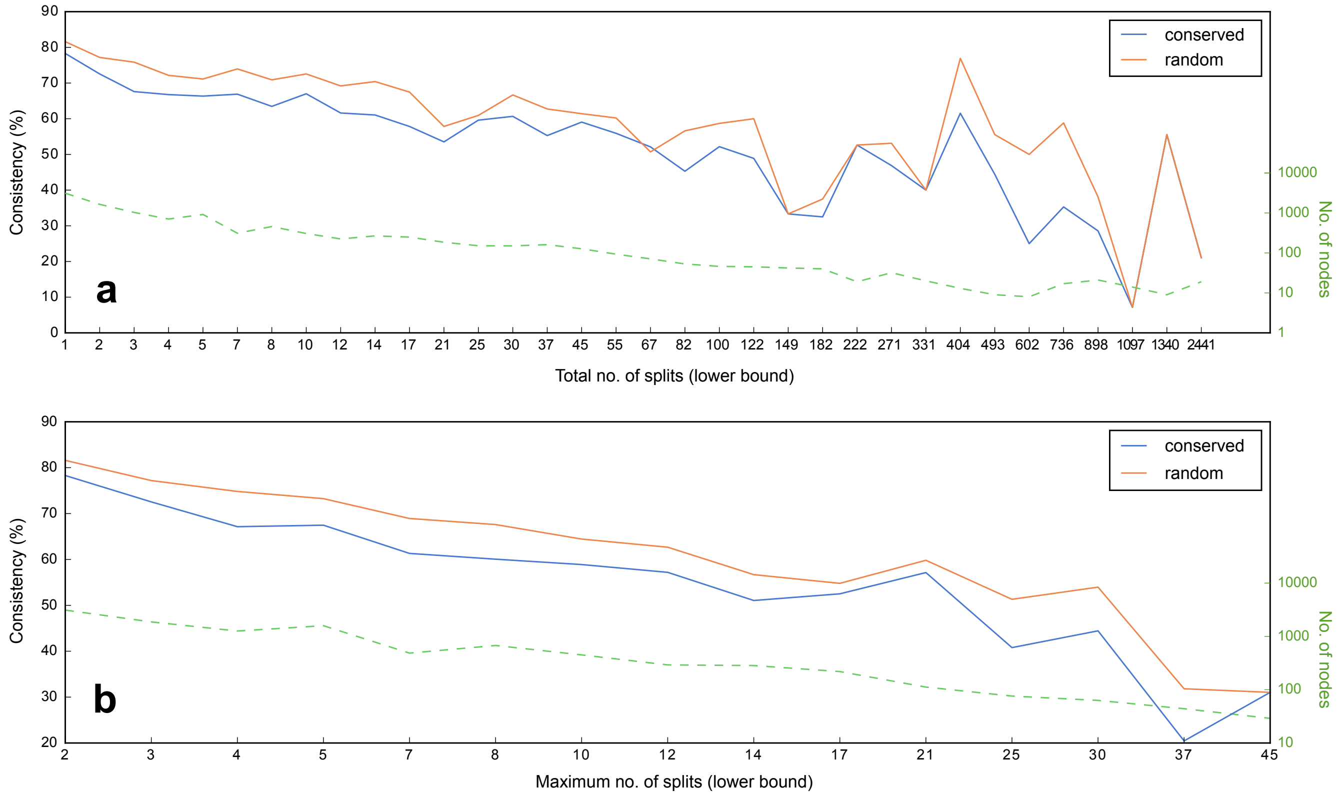

Supplementary Fig. 8. The consistency between the ASTRAL tree and the CONCAT trees by phylogenetic depth. The consistency score (*y*-axis) is the proportion of internal nodes in tree 1 that can be matched to a node in tree 2 which has exactly the same set of descendants. We measured the phylogenetic depth (*x*-axis) using two metrics: **a**. the total number of splits in the clade. This metric was introduced in [96] as the "split depth". The *x*-axis was binned on a roughly logarithmic scale, as determined by Python code: sorted(set(int(math.exp(x/5)) for x in list(range(40)))). Bins with population size (number of nodes) less than five were merged into the next bin. **b**. the maximum number of splits from any tip to the node. The *x*-axis was binned by Python code: sorted(set(int(math.exp(x/5)) for x in list(range(20)))). The per-bin population sizes are indicated by the red dashed lines. Source data are provided as a Source Data file.

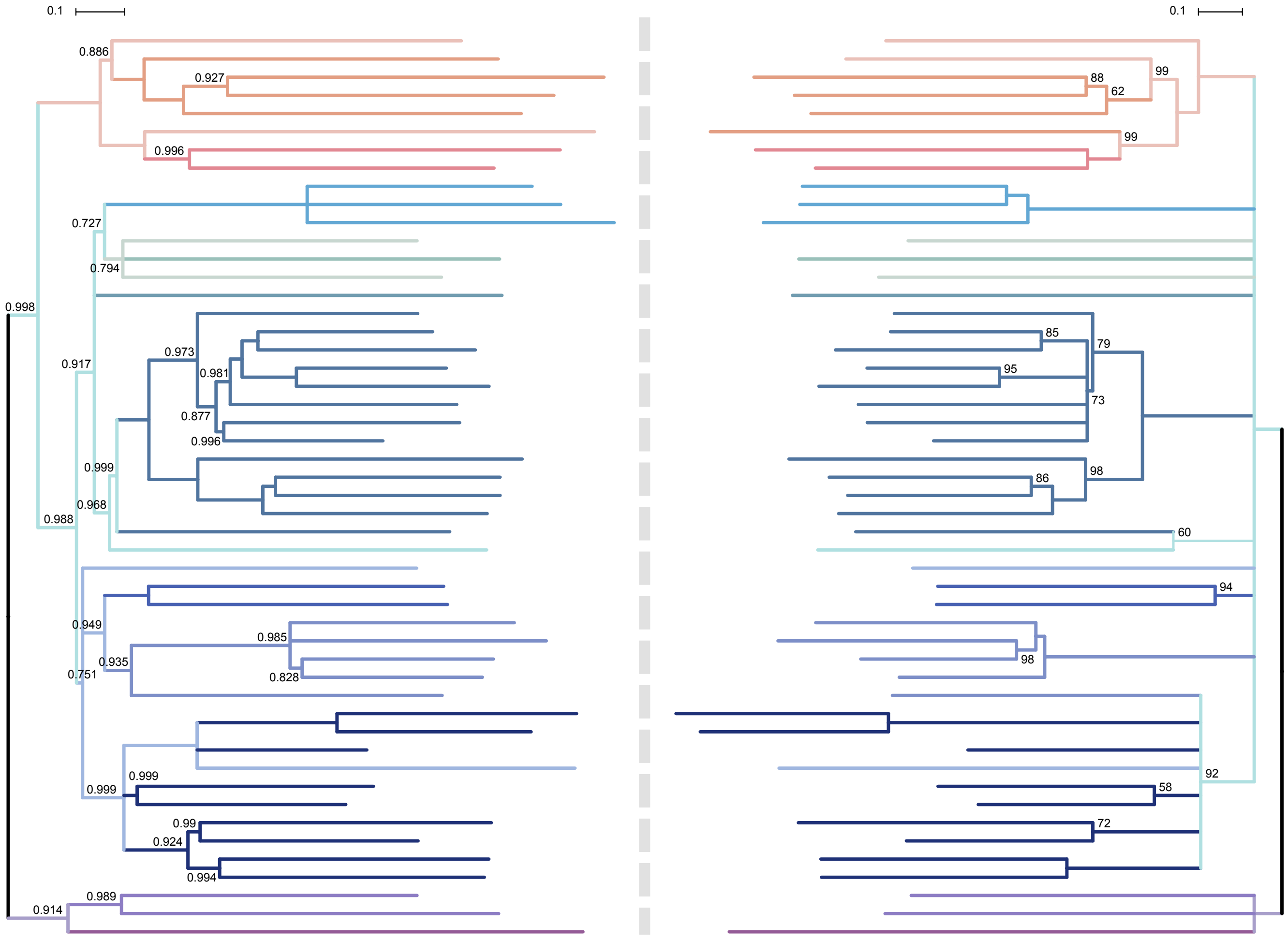

Supplementary Fig. 9. Back-to-back comparison between the ASTRAL tree (left) and the CONCAT tree (right). Both used the conserved site sampling. Low-support branches were collapsed from the two trees to retain the same number of internal nodes per tree. The two trees were then collapsed to 50 shared clades with 50 or more descendants each. A tanglegram was generated to align the clades. Non-full branch support values (local posterior probability for ASTRALand rapid bootstrap for CONCAT) were annotated as node labels. The branches were colored using the same color scheme as in Fig. 1. Source data are provided as a Source Data file.

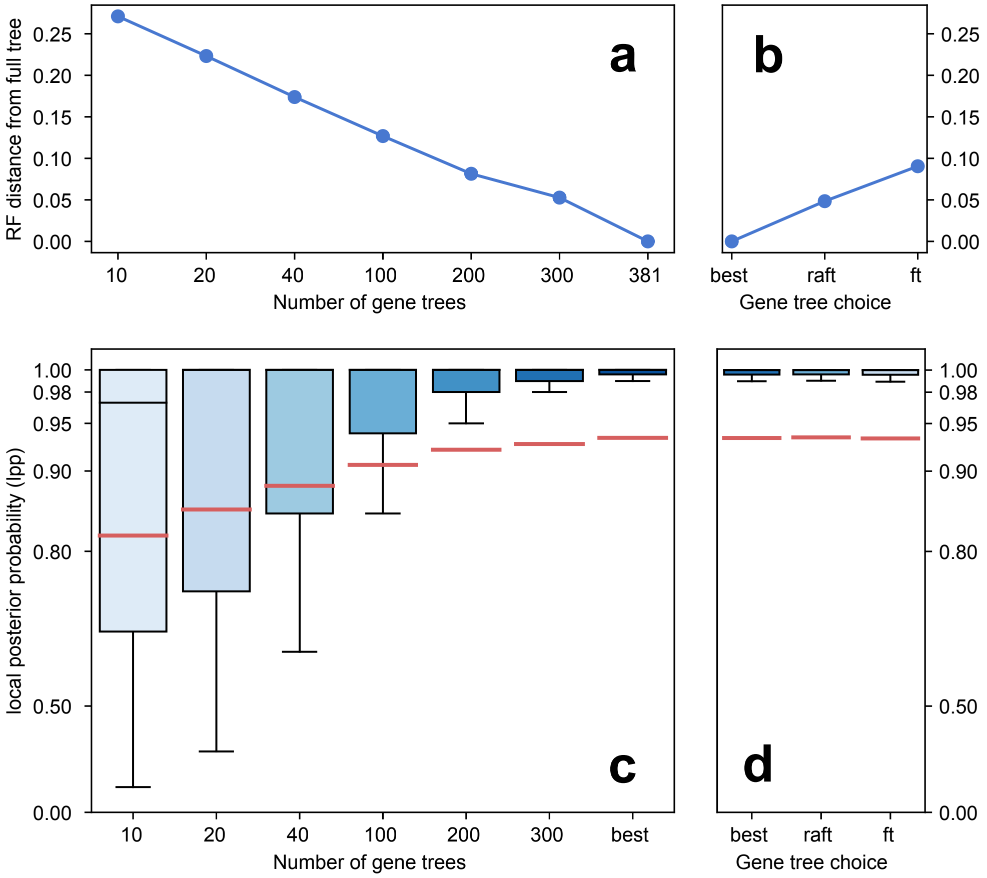

Supplementary Fig. 10. Comparison of ASTRAL species trees built from differential quantity and quality of gene trees. a, c. Series of numbers of gene trees randomly sampled from all 381 gene trees. **a, d**. All gene trees, built and selected using different methods: “ft”: gene trees inferred using FastTree; “raft”: gene trees inferred using RAxML, with the FastTree trees as the starting trees; “best”: for each marker gene, select one tree which has the highest likelihood score from three RAxML runs: one by the FastTree starting tree and other two by random seeds. **a, b**. RF distance from the full-scale reference tree (i.e., “381” in **a** or “best” in **b**). **c, d**. Distribution of branch support values (local posterior probabilities, or lpps). The red lines represent means. The *y*-axis is in exponential scale. Source data are provided as a Source Data file.

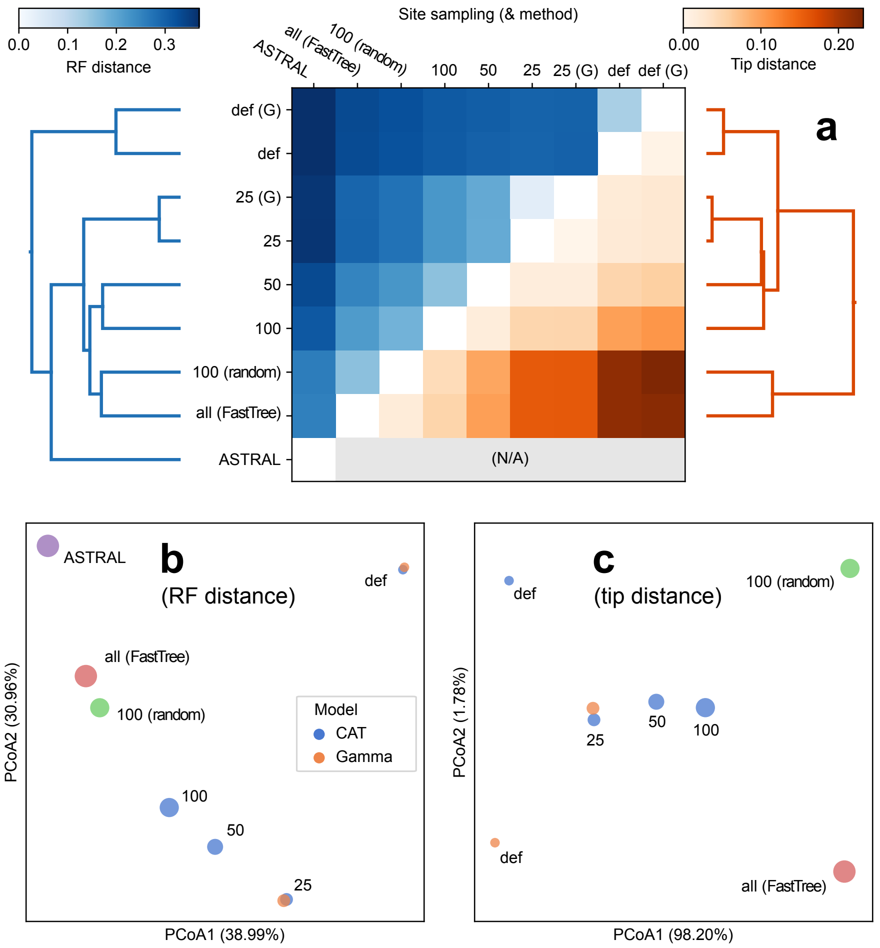

Supplementary Fig. 11. Comparison of CONCAT trees built using different site sampling strategies. a. Heatmap and hierarchical clustering of RF (blue) and tip (orange) distance matrices. The tip distance measures the discorrelation between the two phylogenetic distance matrices among taxa in two trees (see Methods). The full-length marker gene alignments were subsampled based on maximum conservation, at a series of: PhyloPhlAn default ("def"), which approximately yielded 12 sites per gene, and 4.5k sites in total; then 25 sites per gene (9.5k in total), 50 sites per gene (19k in total), and 100 sites per gene (38k sites in total). For def and 25, we were able to perform RAxML tree search under the Gamma model, so the resulting trees were included in this comparison, but for 50 and 100, the use of Gamma model was prohibited by computational challenge. For comparison, we included a tree built on alignments randomly subsampled to 100 sites per gene ("random"), and a tree built on all sites without subsampling, but using FastTree ("all"). Finally, we included the ASTRAL tree, based on gene trees built using all sites, as a reference for comparing topology, but it was not included in the comparison of distances, as the branch lengths inferred by ASTRAL are not comparable to those by CONCAT. **b** and **c**. PCoAs of RF and tip distance matrices, respectively. Source data are provided as a Source Data file.

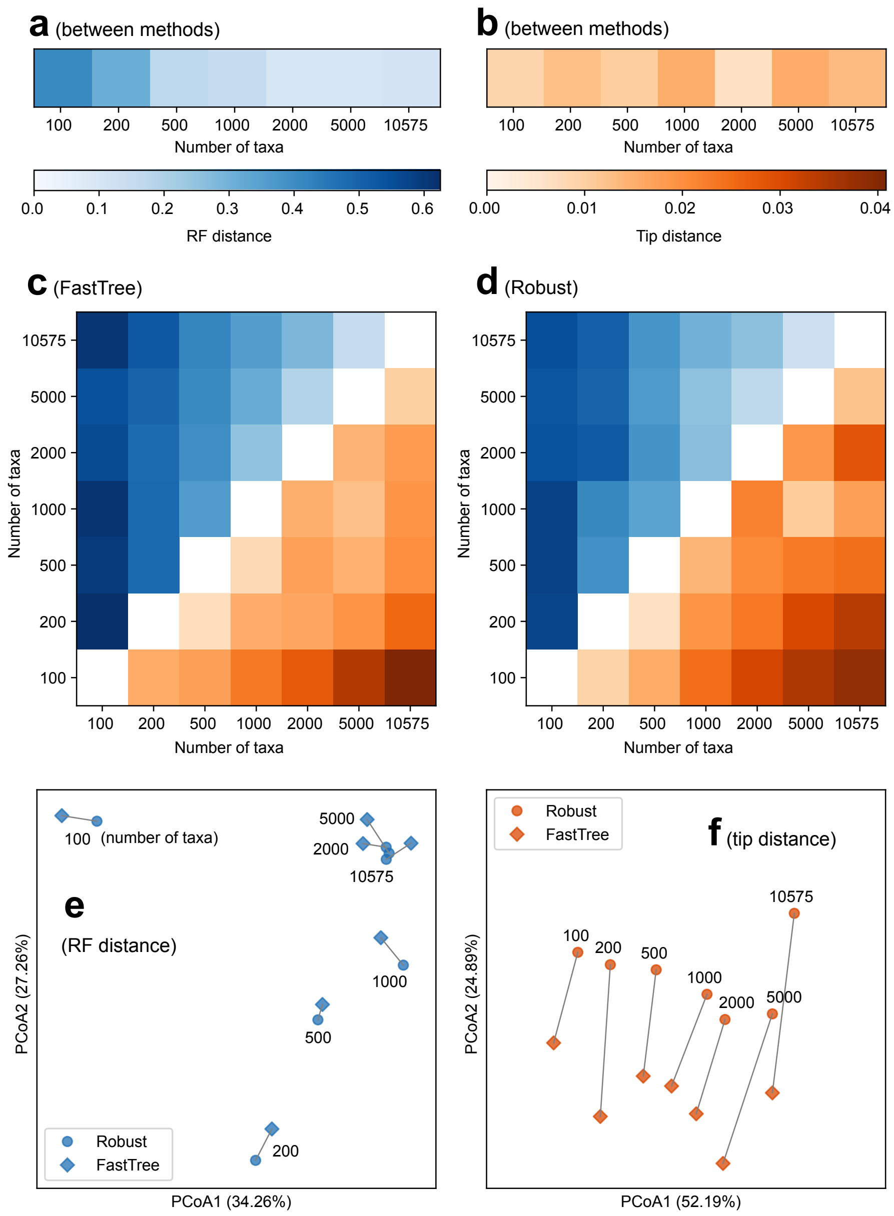

Supplementary Fig. 12. Comparison of species trees built using FastTree and the robust strategy. The "robust strategy" refers to RAxML + CAT for tree topology, and IQ-TREE + Gamma for branch lengths. A series of taxon sets downsampled from the original 10,575 genomes (same as shown in Supplementary Fig. 13) were tested. **a** and **b**. Distances between pairs of FastTree vs. robust trees on the same dataset. **c**. Distances among FastTree trees on different datasets. **d**. Distances among robust trees on different datasets. **e**. PCoA on RF distance matrix among all trees. **f**. PCoA on tip distance matrix among all tree. Pairs of FastTree (diamond) and robust (circle) trees on the same dataset are connected by a line. Source data are provided as a Source Data file.

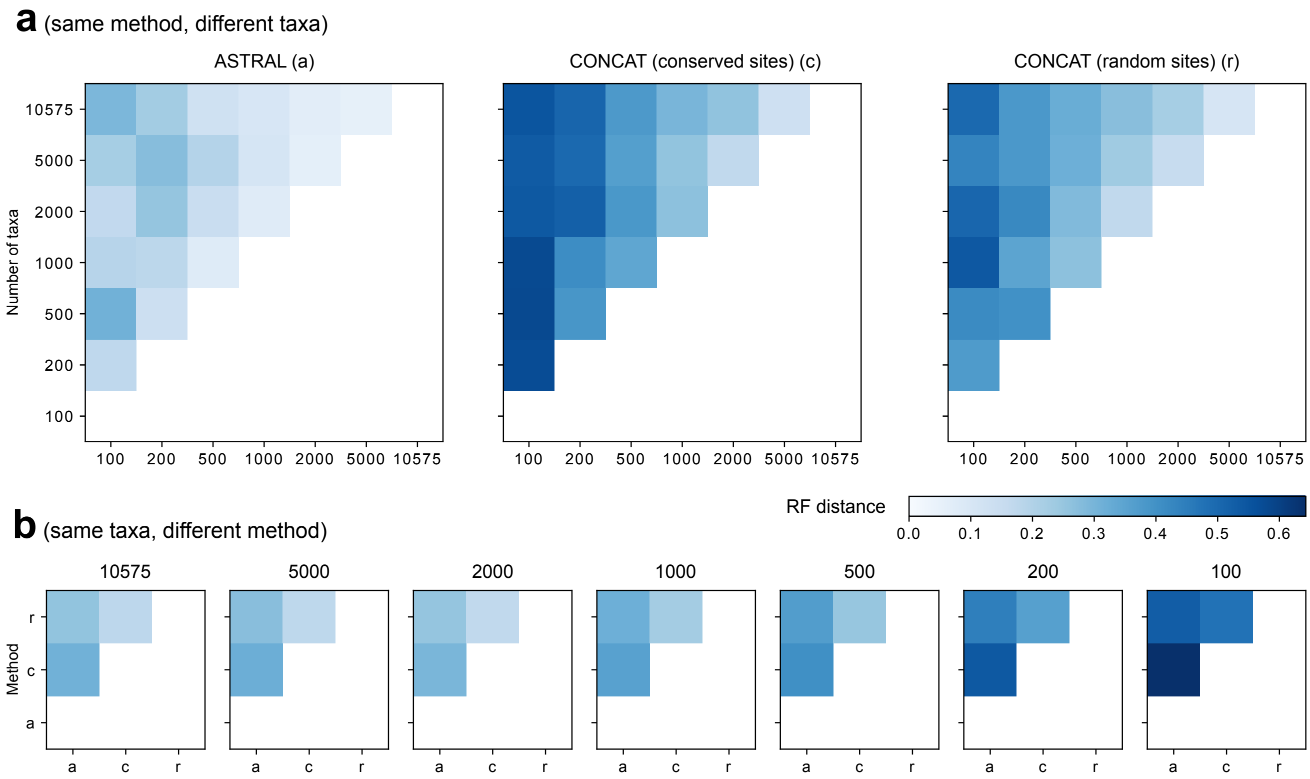

Supplementary Fig. 13. Comparison of species trees built on a series of downsampled taxa. The original 10,575 taxa were subsampled to retain given number (5,000, 2,000, 1,000, 500, 200, and 100) of taxa representative of deep, large clades, as determined using the RED metric (see Methods). Three methods: ASTRAL, CONCAT (using most conserved or randomly selected sites) were evaluated. **a**. RF distance matrices of trees among taxon sets and within each method. **b**. RF distance matrices of trees across methods and within each taxon set. Source data are provided as a Source Data file.

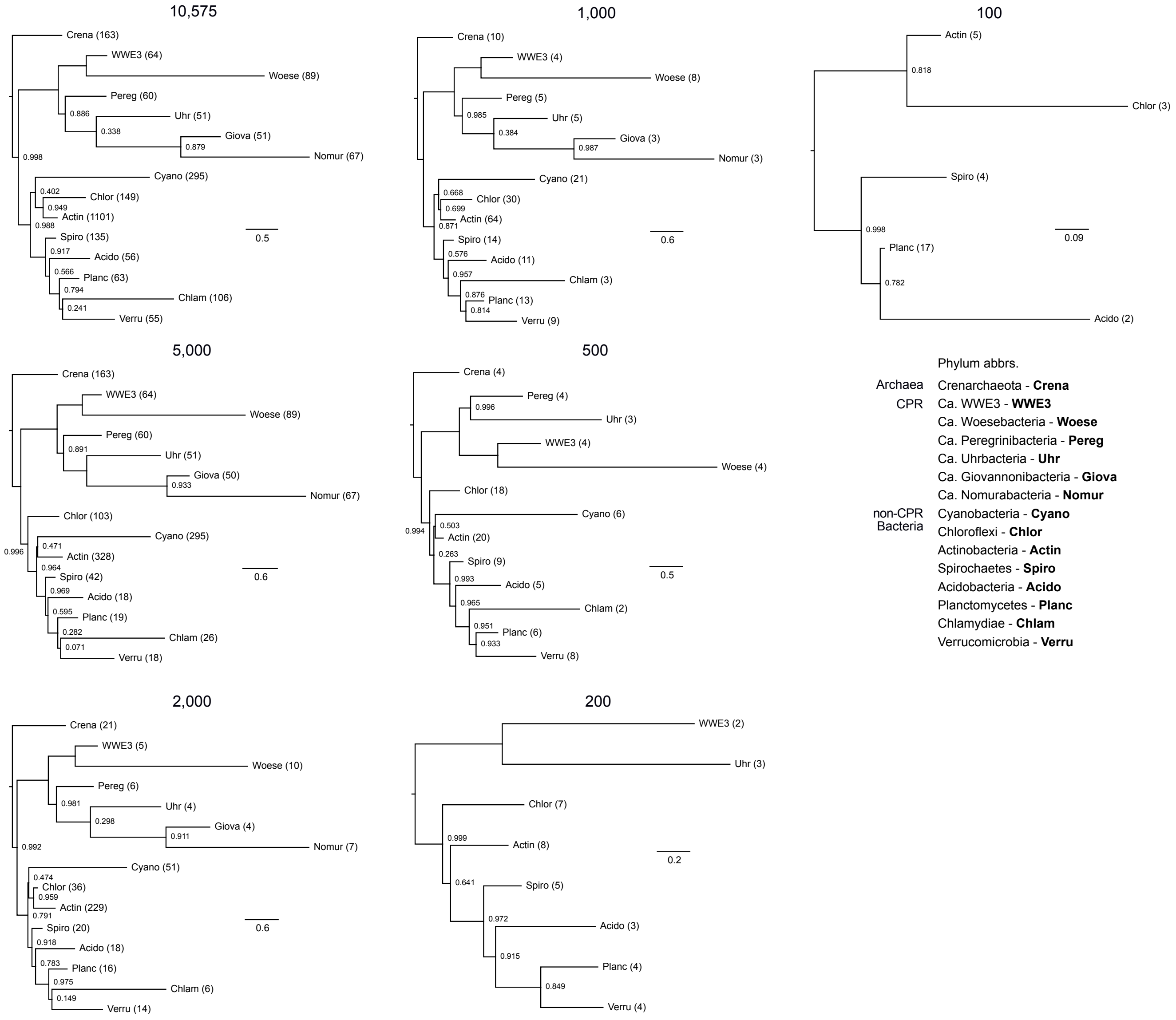

Supplementary Fig. 14. Phylum-level relationships revealed by ASTRAL trees built on series of downsampled taxon sets. Panel headers indicate number of taxa. See Supplementary Fig. 3's caption. Source data are provided as a Source Data file.

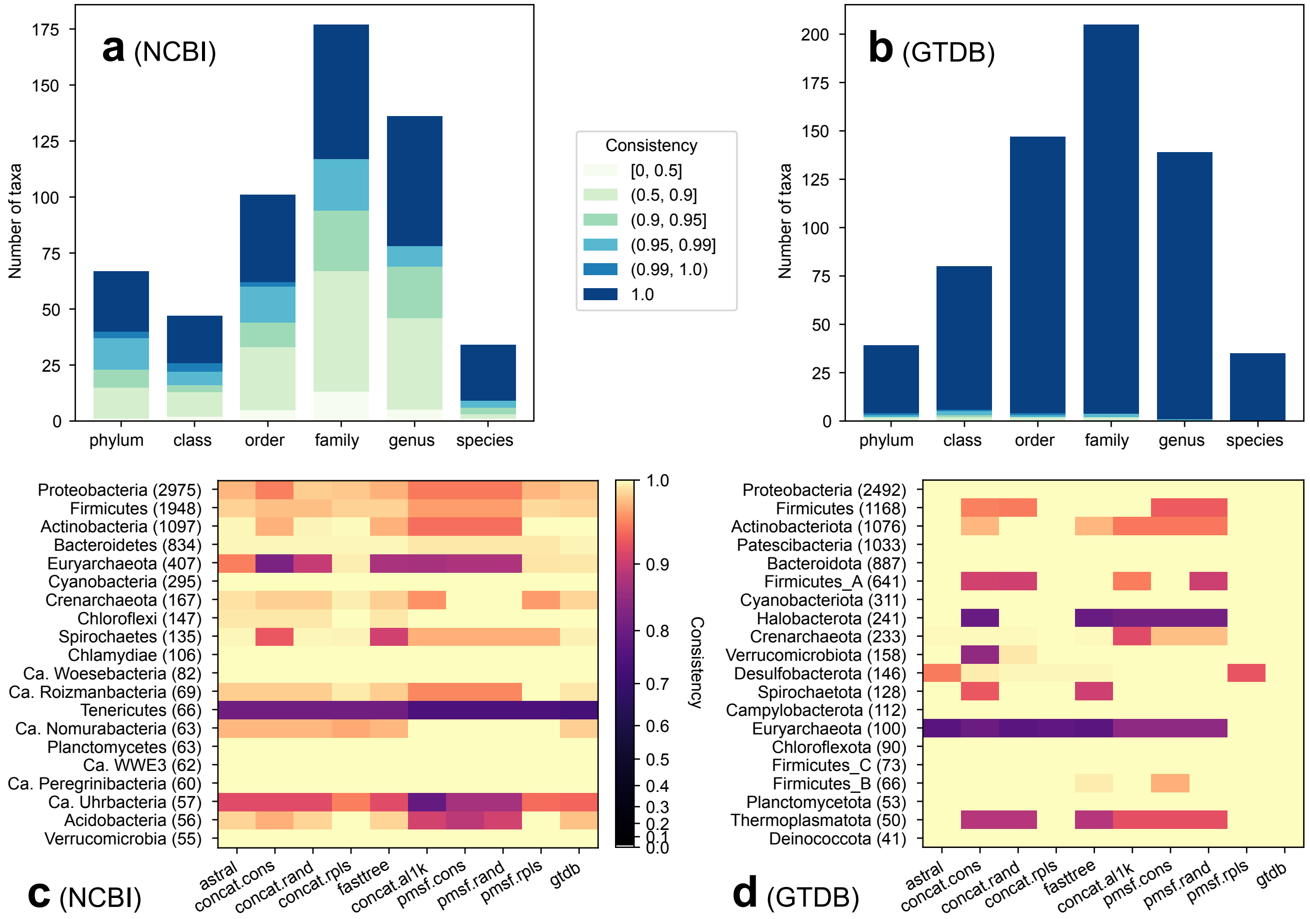

Supplementary Fig. 15. Consistency of taxonomic units with phylogeny. Two taxonomy systems were evaluated: NCBI (**a** and **c**) and GTDB (**b** and **d**). The consistency scores were calculated using tax2tree (see Methods). **a** and **b**. Distribution of consistency scores of taxonomic units with at least ten representatives in the sampled genomes, calculated against the ASTRAL tree. **c** and **d**. Consistency scores of 20 most specious phyla of each system against each of the ten species trees (see Fig. 3). Numbers in parentheses indicate the number of taxa assigned to each group by tax2tree against the ASTRAL tree. Source data are provided as a Source Data file.

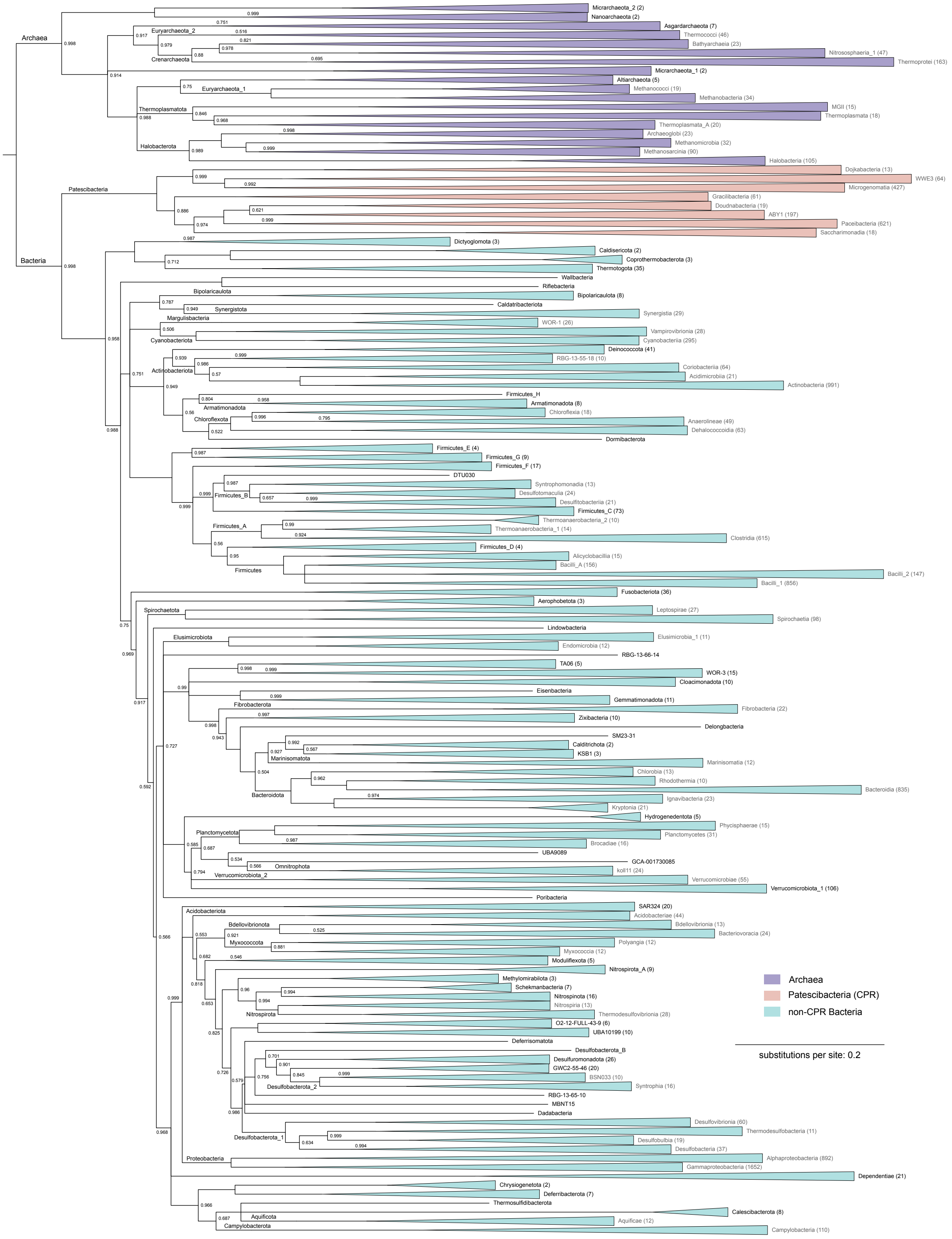

Supplementary Fig. 16. The ASTRAL summary tree annotated using the GTDB taxonomy, collapsed to class level. The tree is identical to that in Supplementary Fig. 4, except for the taxonomic annotations and the alternative collapsing pattern based on taxonomy. The three major groups discussed in this study: Archaea, CPR and non-CPR Bacteria, were colored following Fig. 4a, b. But note that in

GTDB, CPR is classified as phylum Patescibacteria. The triangles represent collapsed clades, with length equal to the longest branch in the clade. Node labels represent local posterior probability (lpp) of the corresponding branch. Labels are omitted at fully-supported (lpp = 1.0) branches. Source data are provided as a Source Data file.

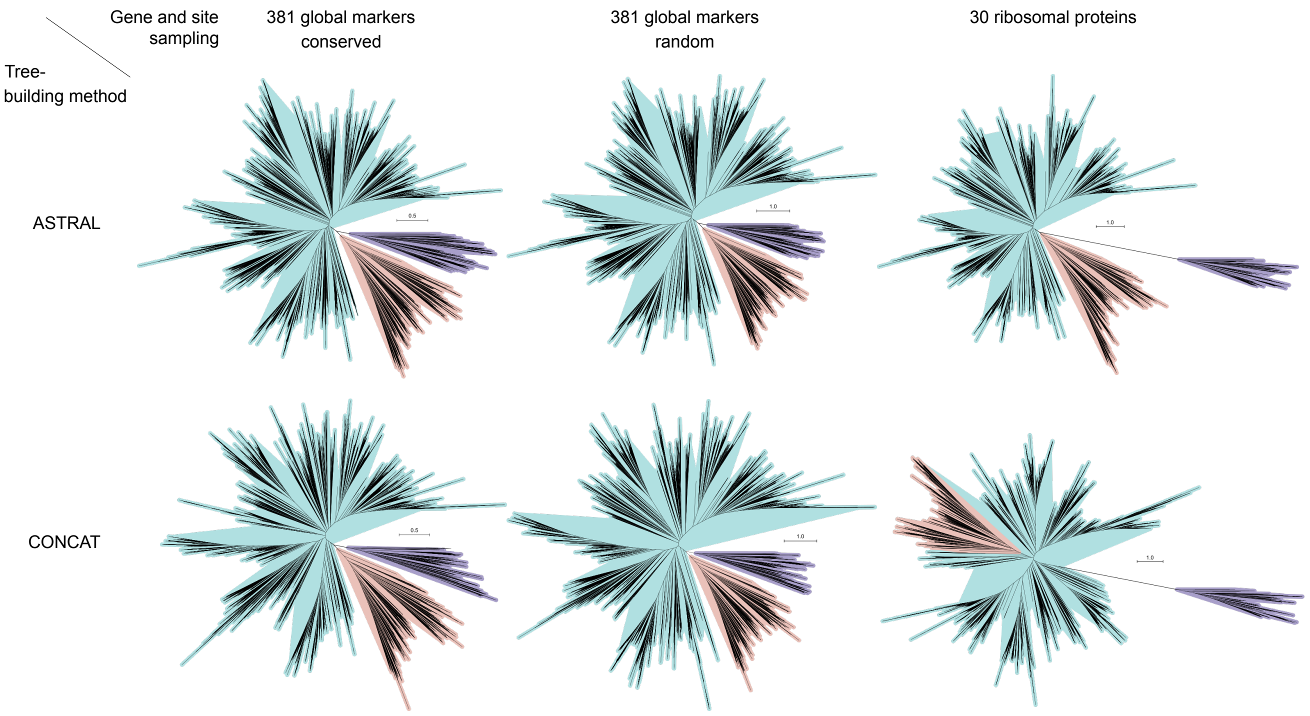

Supplementary Fig. 17. Dimensions and separation of domains Archaea and Bacteria. This extends Fig. 4a, b (with the same color code) to all six trees using different methods (ASTRAL or CONCAT), gene sampling (381 global markers or 30 ribosomal proteins) and site sampling (most conserved or randomly selected). The three top panels are the same topology (the ASTRAL tree), with branch lengths re-estimated using different concatenated alignments. The three bottom panels are different trees separately reconstructed using the corresponding concatenated alignments. Note that in the CONCAT tree by ribosomal proteins, the placement of CPR could not be resolved, thus not depicted as a sister group to non-CPR Bacteria. All trees were drawn to scale, without collapsing or downsampling. Source data are provided as a Source Data file.

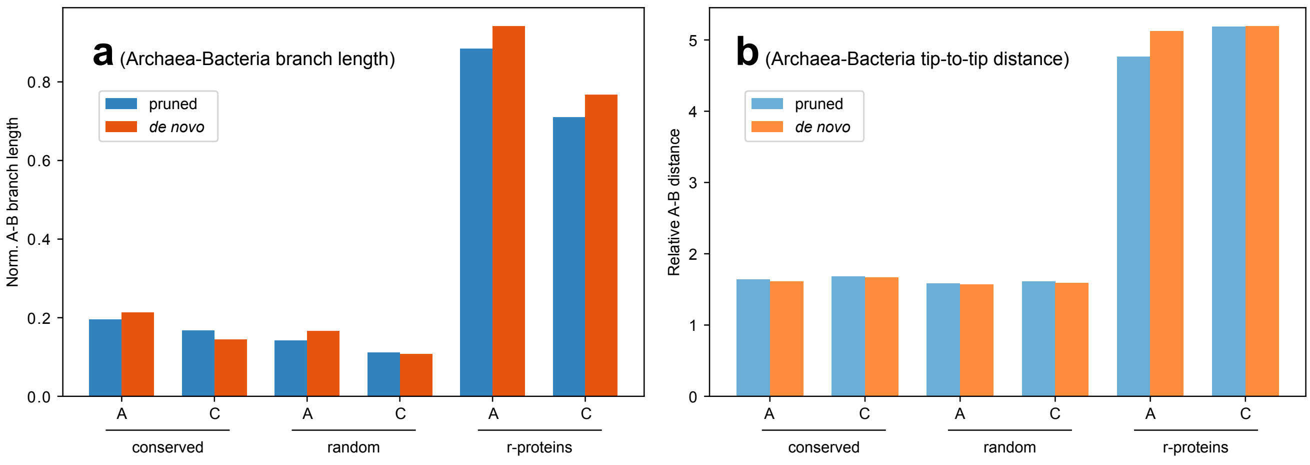

Supplementary Fig. 18. Domain-level phylogenetic distances indicated by trees without CPR taxa. The normalized Archaea-Bacteria branch length (**a**) and the relative Archaea-Bacteria distance (**b**) (see Fig. 4e, f) of each tree are shown. "Pruned" are the same trees from the main results (Fig. 4e, f), with the CPR clade pruned; "*de novo*" are trees reconstructed from CPR-free sequence alignments. Each group contains six trees, built using either (A)STRAL or (C)ONCAT, with either conserved or random site sampling from the 381 global markers, or with the 30 ribosomal proteins. Source data are provided as a Source Data file.

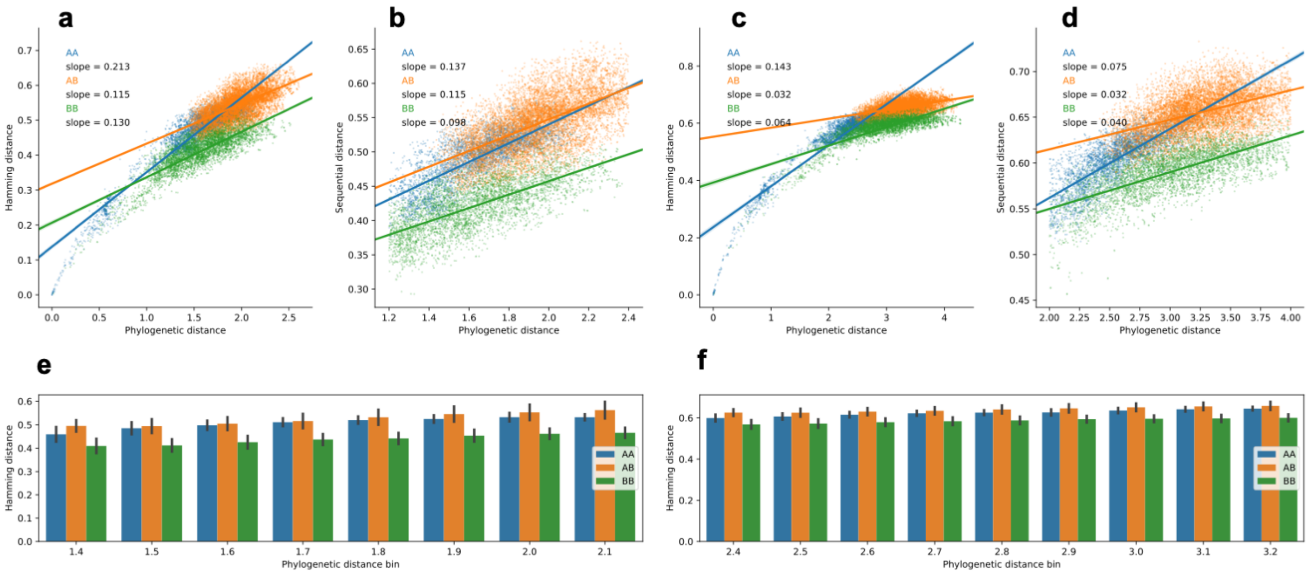

Supplementary Fig. 19. Test for amino acid substitution saturation using conserved or random sites. The pairwise phylogenetic distances (sum of branch lengths) among 100 randomly sampled genomes from each domain are plotted. AA and BB represent intra-domain (Archaea-Archaea and Bacteria-Bacteria, respectively) distances while AB represents inter-domain (Archaea-Bacteria) distances. **a**-**d**: Scatter plots of Hamming distances determined based on pairwise sequence alignments vs. phylogenetic distances. Linear regression lines for the three groups are depicted respectively, with their slopes annotated. **e**-**f**: Phylogenetic distances were binned at equal intervals where each group has a sample size of five or larger. Error bars represent 95% confidence intervals computed from 1,000 bootstraps. The sequence alignments used for computing the Hamming distances were the most conserved sites for **a**, **b** and **e**, and the randomly selected sites for **c**, **d** and **f**. Panels **b** and **d** are zoom-in views of **a** and **c** to show the phylogenetic distance ranges where all three groups are populated. Source data are provided as a Source Data file.

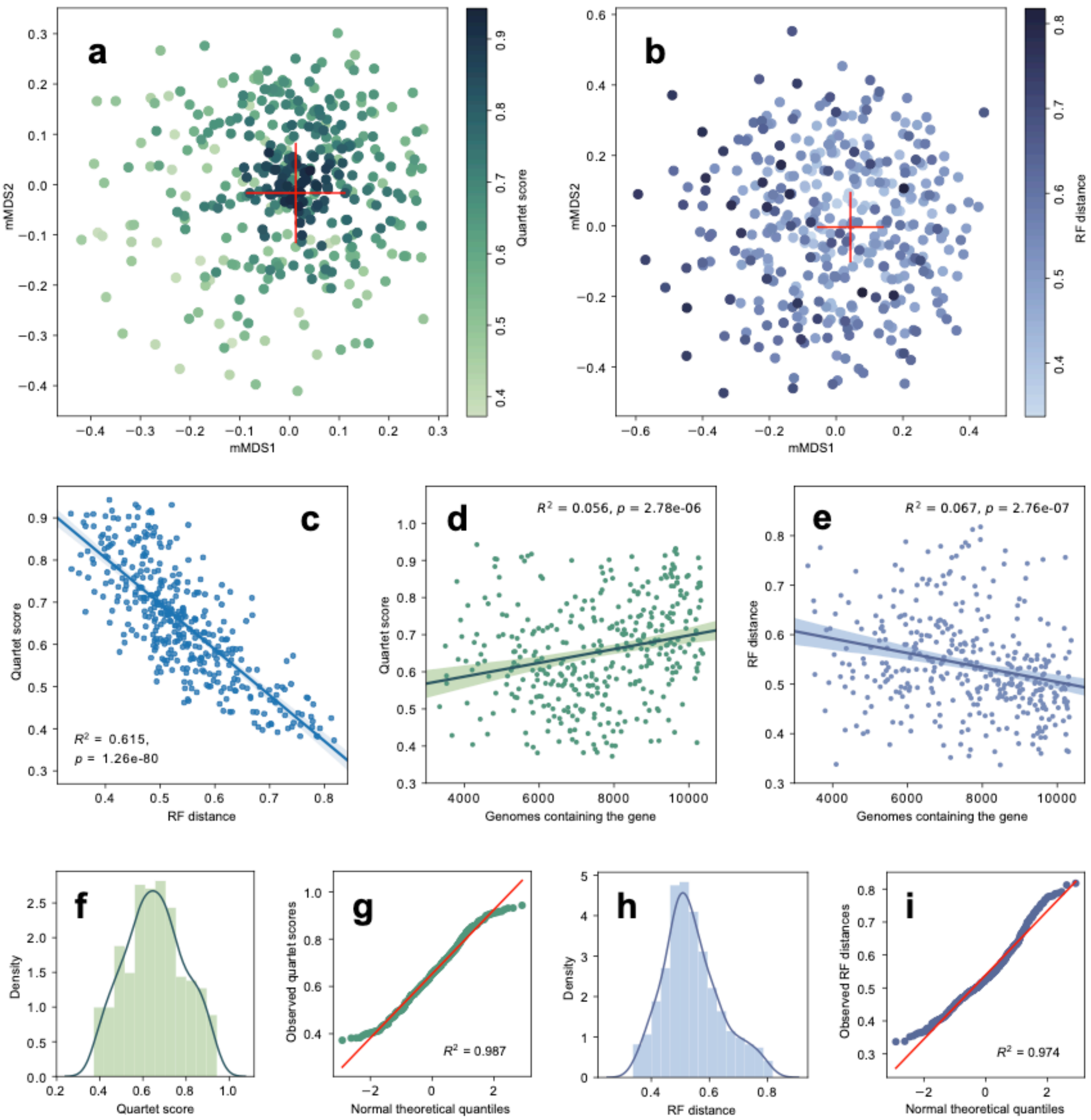

Supplementary Fig. 20. Concordance among individual gene trees and the ASTRAL species tree. **a**. metric multidimensional scaling (mMDS) plot based on the quartet distance (1 - quartet score) between each pair of the 381 gene trees plus the species tree. The center of the red cross indicates the position of the species tree. **b**. mMDS plot based on the Robinson–Foulds (RF) distances. **c**. Linear regression between the quartet score and the RF distance. **d**. Linear regression between the quartet score and the number of genomes in which the corresponding gene was detected. **e**. Linear regression between the RF distance and the number of genomes in which the corresponding gene was detected. The squared

Pearson correlation coefficient (R^2) and two-tailed p-value are displayed for each linear regression. **f**. Histogram and kernel density plot of the quartet scores of the 381 gene trees vs. the species tree. **g**. Quantile-quantile (Q-Q) plot showing how well the quartet scores (y-axis) fit a normal distribution (x-axis). **h**. Histogram and kernel density plot of the RF distances of the gene trees vs. the species tree. **i**. Q-Q plot showing the fitness of the RF distances to a normal distribution. The coefficient of determination (R^2) is displayed for each Q-Q plot. Source data are provided as a Source Data file.

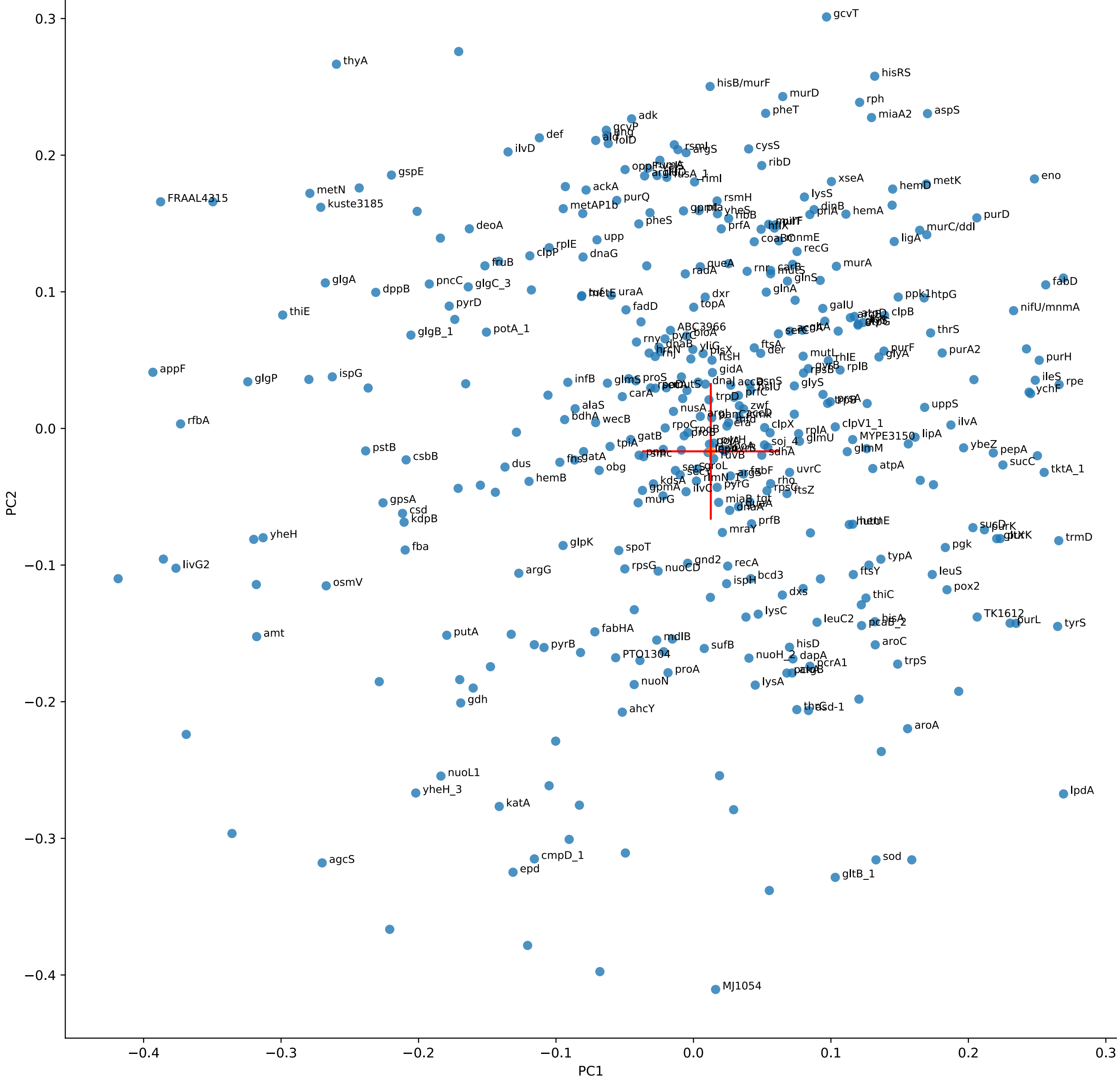

Supplementary Fig. 21. mMDS plot by pairwise quartet distances among the 381 gene trees and the ASTRAL species tree. This is an enlarged view of Fig. 5d and Supplementary Fig. 20a. If a marker gene was annotated with an official gene name from the UniProt database (see https://www.uniprot.org/help/gene_name for rules), the corresponding gene tree is labeled with that name. Source data are provided as a Source Data file.

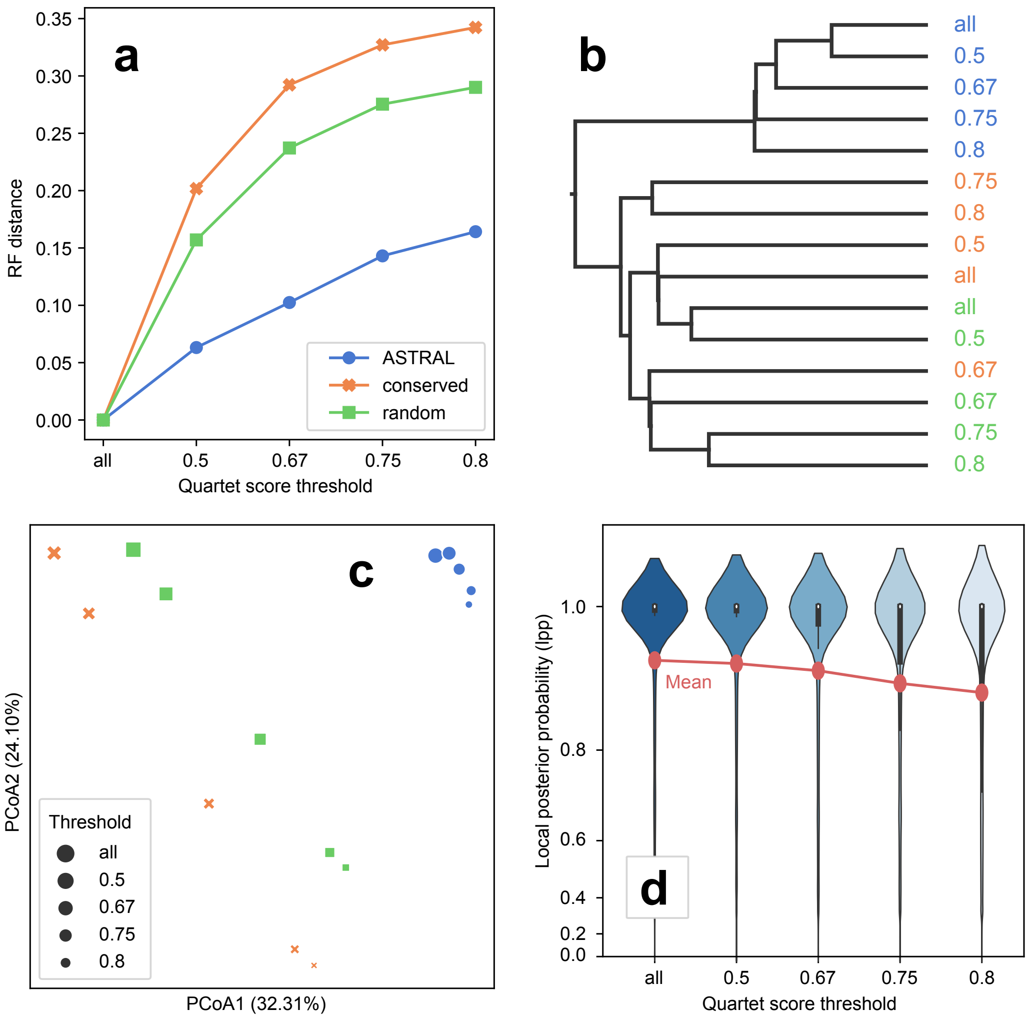

Supplementary Fig. 22. Comparison of species trees built using marker genes subsampled by quartet score. The 381 marker genes (all) were downsampled to subsets in which the quartet score of the corresponding gene tree is at least 0.5 (322 genes), 0.67 (171 genes), 0.75 (93 genes) and 0.8 (64 genes), respectively. Three methods: ASTRAL (blue), CONCAT by most conserved sites (orange) or randomly selected sites (green) were tested. **a-c**: Topological discrepancy between pairs of trees, as measured by the Robinson–Foulds (RF) distance. **a**. RF distance from tree on each subset to the full-scale tree ("all") by method. **b**. Hierarchical clustering of the RF distance matrix. **c**. PCoA of the RF distance matrix. **d**. Violin plots of distribution of ASTRAL tree branch supports (lpp) on each subset. The red lines represent means. The *y*-axis is in exponential scale. Source data are provided as a Source Data file.

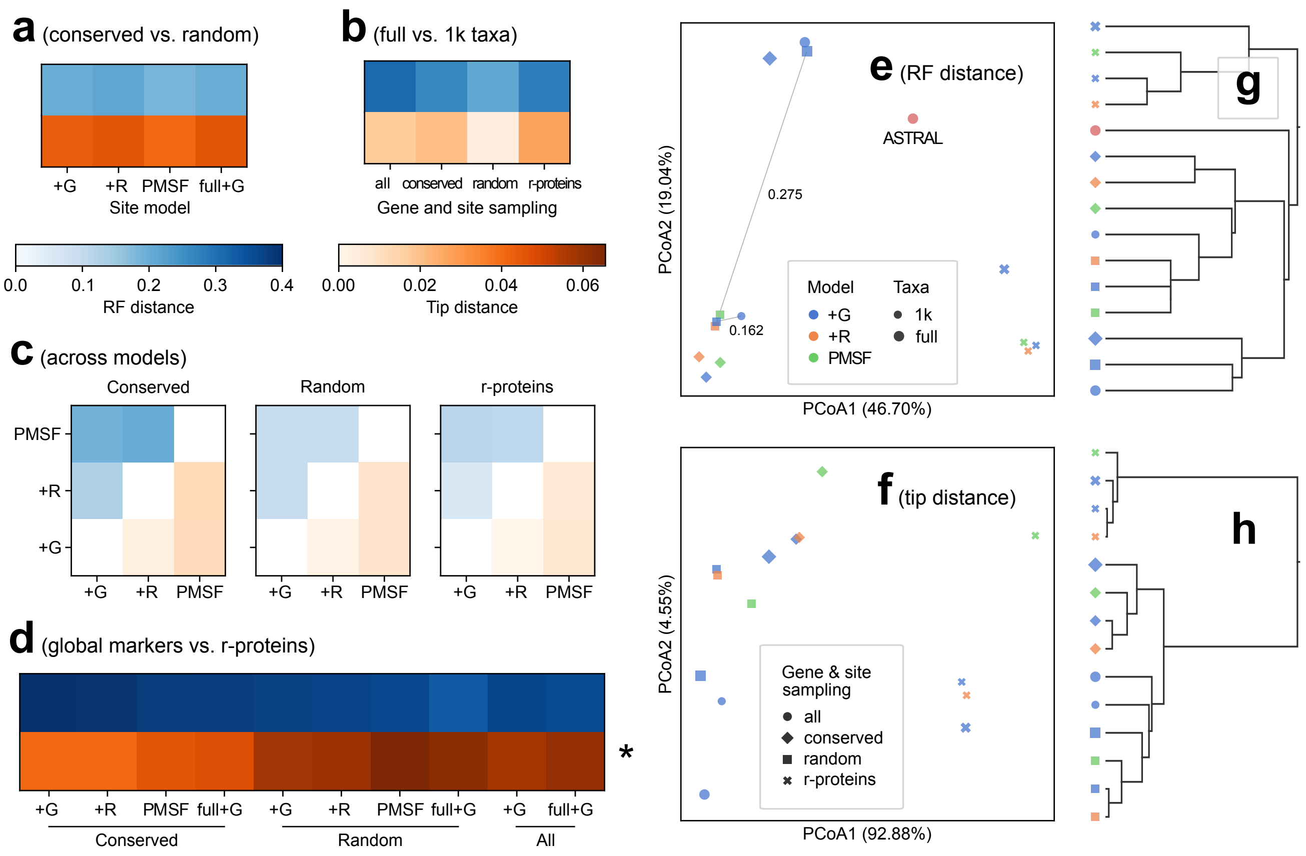

Supplementary Fig. 23. Comparison of CONCAT trees on downsampled 1,000 taxa and alternative site models. The 1,000-taxon set is the same as shown in Supplementary Fig. 13. Three models are compared: “+G”: the conventional Gamma model, i.e., the rate heterogeneity across sites is subject to a Gamma distribution; “+R”: the FreeRate model, which relaxes the assumption of Gamma distribution of rates; “PMSF”: the posterior mean site frequency model, which operates on site profiles determined by the profile mixture model C60 (selected in a model test). As controls, the 10,575-taxa full-scale CONCAT trees were truncated to the 1,000 taxa for comparison (“full+G”). Blue: RF distances. Orange: tip distances. **a**. Distances between trees by differential site sampling: most conserved or randomly selected sites. **b**. Distances between trees by differential taxon sampling: 10,575 (full) or 1,000 taxa, both using the Gamma model. **c**. Distances among trees by different site models. **d**. Distances among trees based on the 381 global marker genes or the 30 ribosonal proteins. Note (*) that the tip distances illustrated in this panel were divided by three, otherwise they would be too dark to allow other panels being distinguishable. **e**. PCoA of RF distance matrix. A special comparison between the impact of site sampling vs. that of taxon sampling was highlighted by grey lines, and the corresponding RF distances were annotated. **f**. PCoA of tip distance matrix. **g**. Hierarchical clustering of RF distance matrix. **h**. Hierarchical clustering of tip distance matrix. For **e** and **g**, the ASTRAL tree (red)

was included as a reference, but it was not included in **f** and **h** because ASTRAL does not directly compute branch lengths in unit of substitutions per site. Source data are provided as a Source Data file.

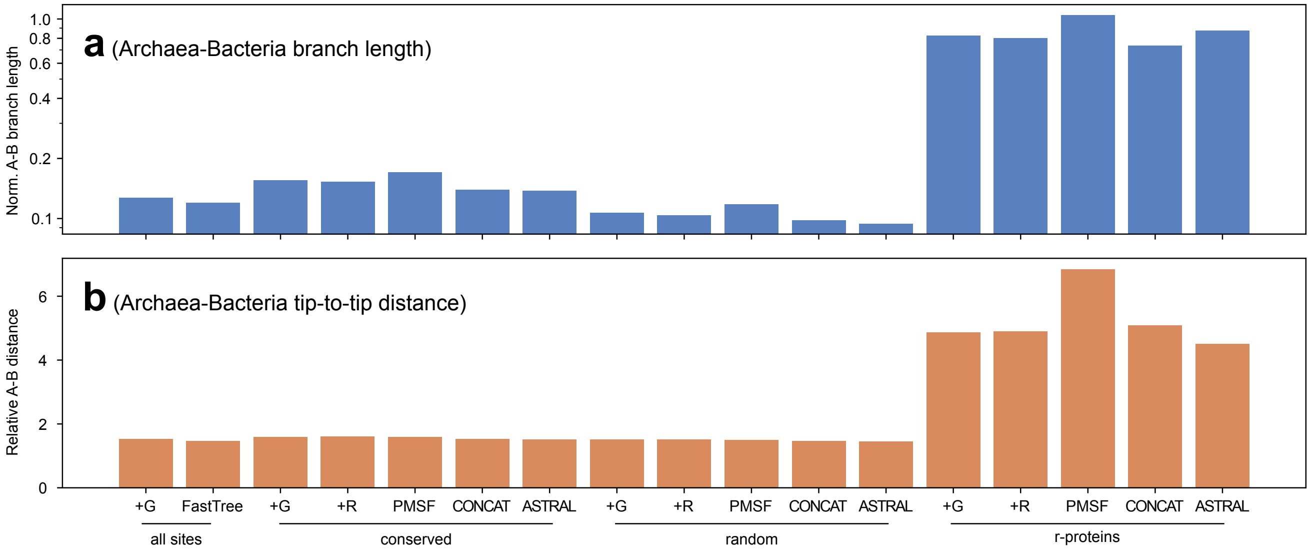

Supplementary Fig. 24. Domain-level phylogenetic distances indicated by the 1,000 downsampled taxa. The normalized Archaea-Bacteria branch length (**a**) and the relative Archaea-Bacteria distance (**b**) (see Fig. 4f) of each tree are shown. Being compared are trees reconstructed based on the 1,000 taxa (+G, +R and PMSF), and trees inferred based on all 10,575 taxa but pruned to retain the same 1,000 taxa (FastTree, CONCAT and ASTRAL, branch lengths all based on the Gamma model). Note that these metrics are not directly comparable to those of the full-scale trees shown in Fig. 4e, f, due to taxon downsampling. Source data are provided as a Source Data file.

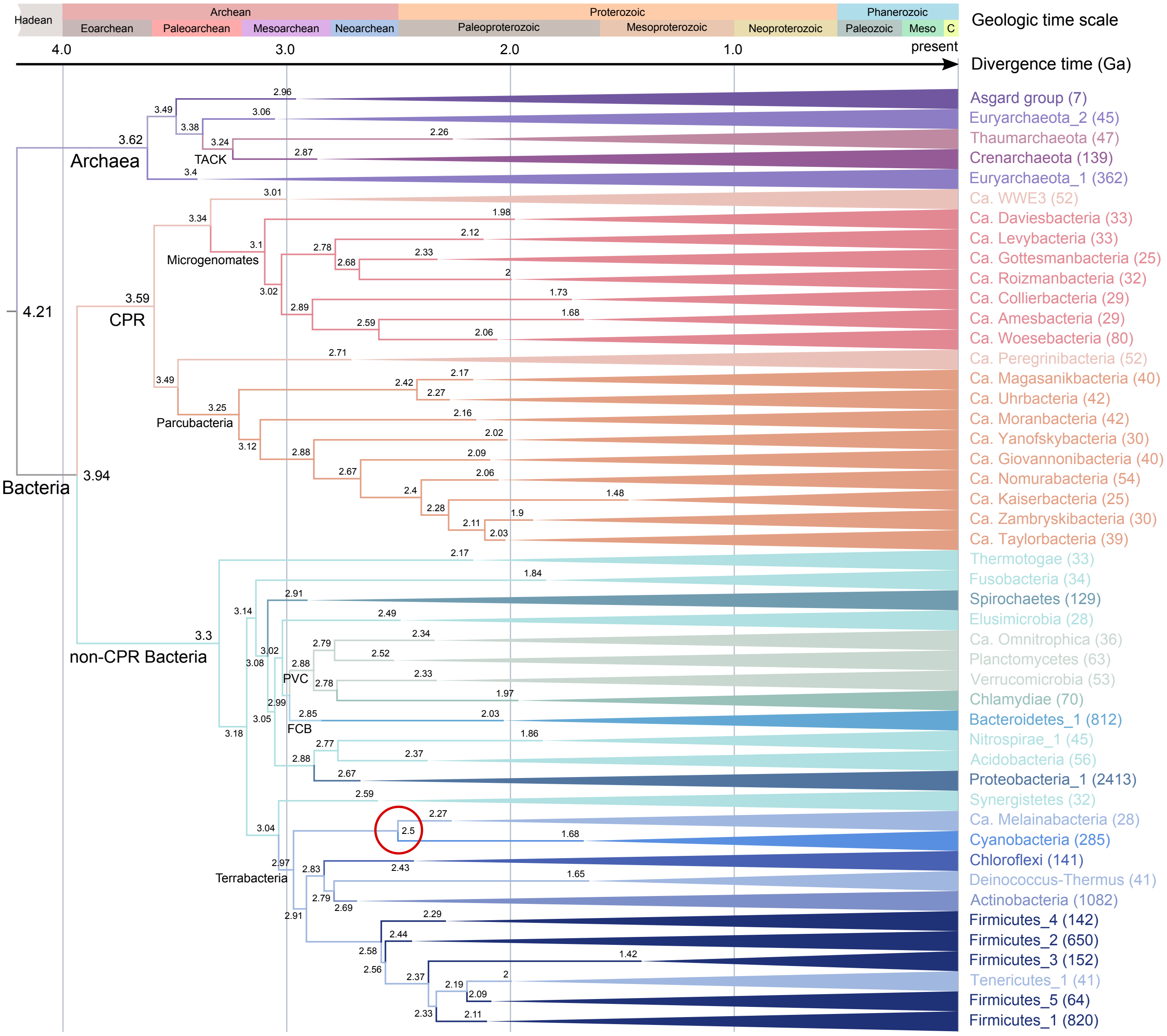

Supplementary Fig. 25. Chronogram of microbial evolution inferred using maximum likelihood with a strict clock model. The evolutionary times were inferred based on the ASTRAL tree with branch lengths re-estimated using the conserved sites, and calibrated by the predicted emergence of the photosynthetic cyanobacteria (indicated by a red circle). For display purpose, clades representing phyla with at least 25 descendants were preserved and collapsed as triangles. Node labels represent the time in Ga (billion years ago) estimated by the run with the best likelihood score out of 10 replicates. The color scheme is consistent with Fig. 1. Source data are provided as a Source Data file.

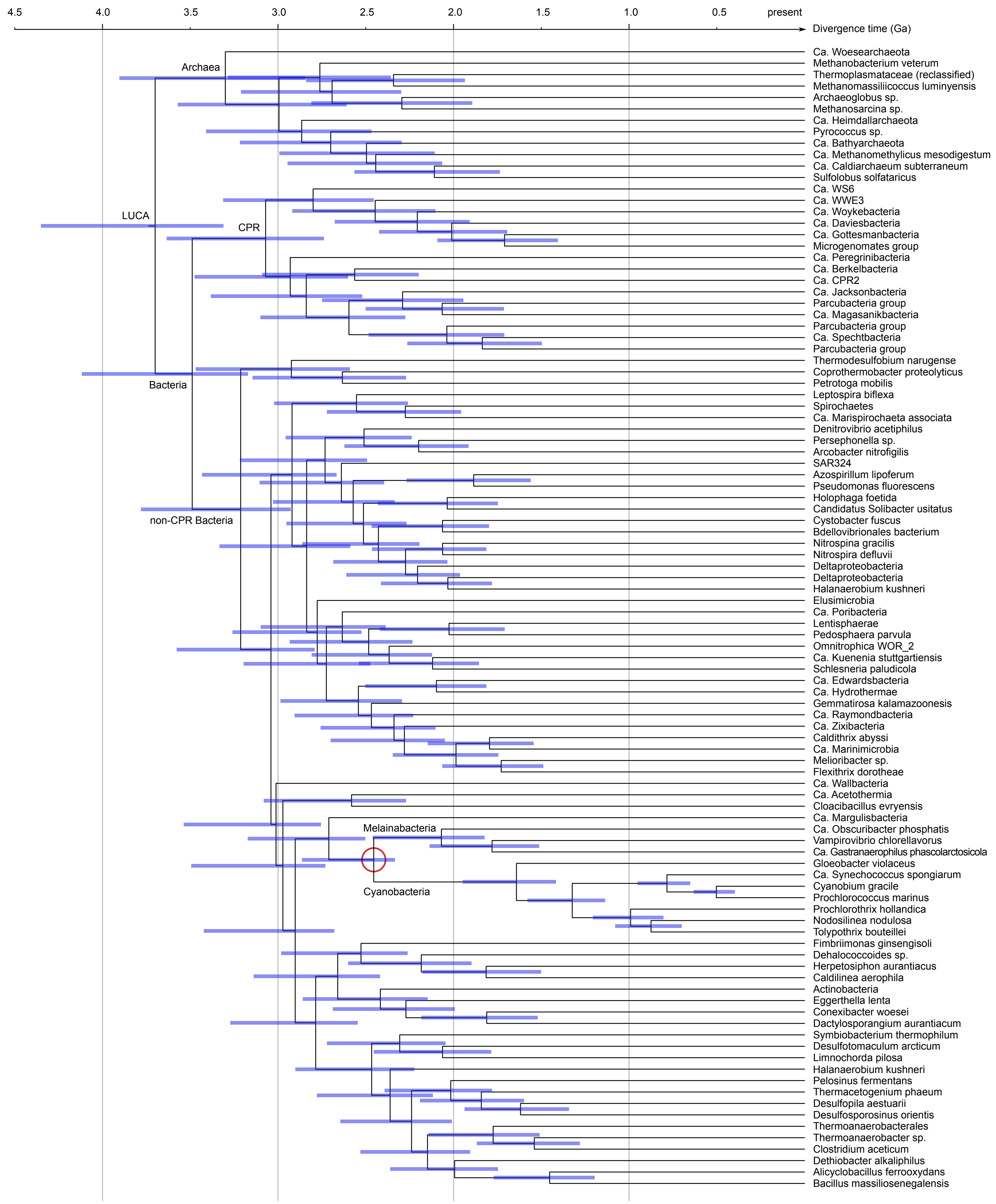

Supplementary Fig. 26. Chronogram of microbial evolution inferred using Bayesian with a relaxed clock model. One hundred taxa by 5,000 randomly sampled sites were included in this analysis. The tree topology is identical to the ASTRAL tree. The node where time constraint (using the “wide”

prior distribution) was placed on is indicated by a red circle. Node ages were estimated using BEAST, with an uncorrelated lognormal relaxed clock model (UCLD). Taxon labels are the Latin species names, wherever available, omitting strain names, or the higher rank (usually phylum or superphylum) name if underclassified. Node heights represent the median of sampled age estimates of the node. Node bars indicate 95% confidence intervals. Source data are provided as a Source Data file.

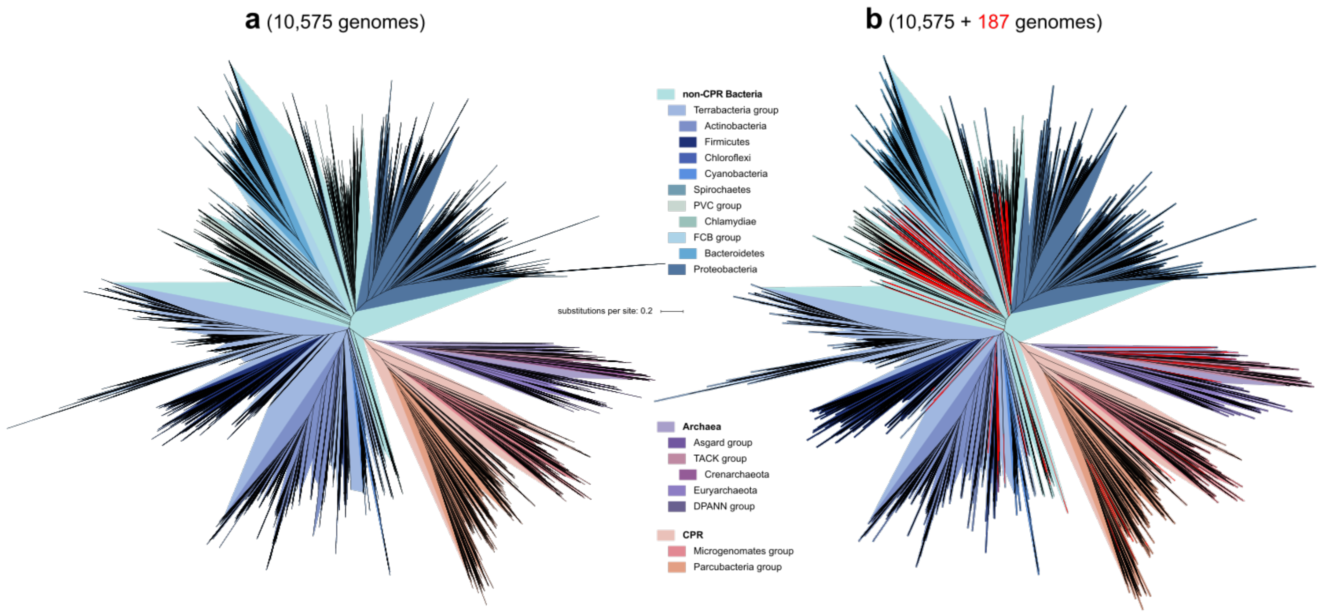

Supplementary Fig. 27. Consistency of reconstructed evolutionary relationships with newly discovered microbial diversity. Both trees were built using ASTRAL on the 381 marker genes, and the branch lengths were estimated using up to 100 most conserved sites per gene. Color codes of clade shadows are consistent with Fig. 1. The trees are drawn-to-scale, with all taxa displayed. **A**. Tree of 10,575 genomes, which is the same as shown in Figs. 1, 3a and S5. **B**. Tree of the same 10,575 genomes plus 187 new genomes as of May 2019, representing previously missing or underrepresented NCBI and GTDB phyla. Clades constituted of the new genomes are colored red. Source data are provided as a Source Data file.

Supplementary Tables

Supplementary Table 1. A summary of previous and current trees of microbial life.

Name	Date	Publication	Domain(s)	Phylogenetic tree			Character matrix			Related works
				Taxa	Gene(s)	Method	Taxa	Characters	Unit	
Woese and Fox	1977-11-01	270744	A, B, E	13	SSU	"comparative"	N/A	N/A	bp	2112744
Barns et al.	1996-08-20	8799176	A, B, E	64	SSU	fastDNAml	64	N/A	bp	9115194
Ciccarelli et al.	2006-03-03	16513982	A, B, E	191	31	PhyML	181	999,326	aa	
LTP rel. 93	2008-08-09	18692976	A, B	6,727	SSU	RAxML	9,975	14,576,220	bp	
AMPHORA	2008-10-13	18851752	B	578	31	PhyML	578	4,033,260	aa	20033048
Cox et al.	2008-12-23	19073919	A, B, E	40	45	P4	40	N/A	aa	24336283
Greengenes rel. 13_5	2013-05-20	22134646	A, B	203,452	SSU	FastTree	203,452	260,068,849	bp	
Lang et al.	2013-04-25	23638103	A, B	841	24	BUCKy	840	3,601,341	aa	
GEBA-MDM	2013-07-14	23851394	B	2,229	38	RAxML	2,228	16,304,266	aa	
PhyloPhlAn	2013-08-14	23942190	A, B	3,737	400	RAxML	3,139	10,399,954	aa	
Hug et al.	2016-04-11	27572647	A, B, E	3,083	16	RAxML	3,080	6,532,247	aa	29522741
1,003 GEBA genomes	2017-06-12	28604660	A, B	1,003	56	RAxML	1,039	17,750,144	aa	
Schulz et al.	2017-10-17	29041958	B	12,400	SSU	RAxML	926	1,343,426	bp	
GTDB rel. 80	2018-08-27	30148503	B	21,943	120	FastTree	21,547	650,103,222	aa	28894102
this work	N/A	N/A	A, B	10,575	381	RAxML	10,474	273,417,890	aa	
							10,485	265,218,697	aa	
						ASTRAL	10,575	1,162,421,084	aa	

The table summarizes representative phylogenetics studies that featured the global taxon sampling of one or multiple domains of microbial life forms. Only works involving *de novo* phylogenetic reconstructions based on the entire datasets were selected (thus excluding synthesis studies such as the Open Tree of Life [97]). The name of each work is either the project name plus the release version, if applicable, or in the “authors (year)” format. “Date” is the date of the release (if applicable) or the publication. “Publication” is the NCBI PMID of the article. For one work containing

multiple trees, only one tree that was based on the largest dataset, built using the most expensive method, or recommended by the authors was recorded. For one series of closely related works, only one work that was most relevant in the context of "tree of life" was recorded, while the others were mentioned in the "related works" field. "Domain(s)" codes are (A)rchaea, (B)acteria and (E)ukaryota. In some works (such as GEBA-MDM and GTDB), because taxa from different domains were subjected to separate phylogenetic reconstructions, only the largest domain (Bacteria) was recorded. Whenever possible, the actual dimensions of the phylogenetic tree and the supporting character matrix (i.e., a multiple sequence alignment, excluding duplicates) were recorded. "Characters" is the sum of non-missing, non-gap characters (unit: bp (basepair) or aa (amino acid)). Note that the numbers of taxa in the tree and in the matrix may be different due to filtering and clustering operations.

Supplementary Table 2. Computational expenses for building the phylogenies of 10,575 microbial genomes based on 381 marker genes.

**1. Pre-tree-building steps**	Marker extraction (x10575) (PhyloPhlAn)		Alignment (x381) (PASTA / UPP)		Model selection (x381) (RAxML)		Total		
	runtime (hr)	CPU hrs (x32)	runtime (hr)	CPU hrs (x3)	runtime (hr)	CPU hrs (x4)	CPU hrs		
	17.78	568.96	772.12	2316.36	932.90	3731.60	6616.92		
**2. Tree-building - summary**	Gene tree building (x381) (starting tree) (FastTree)		Gene tree building (x381) (RAxML / IQ-TREE)		Gene tree summarization (ASTRAL)			Total	
	runtime (hr)	CPU hrs (x4)	runtime (hr)	CPU hrs (x24)	runtime (hr)	CPU hrs (x28)	GPU hrs (x4)	CPU hrs	GPU hrs
	213.39	853.56	3980.87	95540.88	9.96	278.85	39.84	96673.29	39.84
**3. Tree-building - concatenation**	Starting tree building (FastTree)		Tree topology search (RAxML + CAT)		Tree optimization (IQ-TREE + Gamma)		Rapid bootstrap (x100) (RAxML + CAT)		Total
site sampling	runtime (hr)	CPU hrs (x3)	runtime (hr)	CPU hrs (x24)	runtime (hr)	CPU hrs (x24)	runtime (hr)	CPU hrs (x24)	CPU hrs
conserved	6.79	20.37	143.20	488.88	1.55	37.27	1362.58	32701.80	33248.32
random	7.03	21.09	156.45	506.16	1.37	32.93	1487.49	35699.69	36259.87

For each procedure, the runtime (wall-clock time) is listed, and the charged time (CPU hours or GPU hours) was obtained by multiplying the runtime by the number of CPU cores or GPU units allocated (shown in parentheses). Times for procedures that were inexpensive or not directly relevant to the tree-building have been omitted. Several steps consisted of multiple independent jobs that can be effectively parallelized. The number of jobs is indicated in parentheses after the procedure title. Several steps actually consisted of multiple trials (e.g., we did three runs per maximum likelihood tree building and selected the one with the highest Gamma likelihood), but in this table we only report the times of the selected trials. Thus, this table indicates the minimum time required for building the phylogenies we present.

Supplementary Table 3. Summary of NCBI taxonomy curated based on phylogeny.

Rank	Same	Add	Change	Delete	Empty
phylum	10000	158	50	177	190
class	7900	304	90	90	2191
order	7625	439	183	104	2224
family	7074	423	299	251	2528
genus	6655	159	350	624	2787
species	10229	0	247	99	0

"Same": validated the original assignment; "Add": assigned a taxon to an originally unassigned rank; "Change": modified an originally incorrectly assigned taxon; "Delete": deleted an originally incorrectly assigned taxon; "Empty": unassigned in both original and curated taxonomy.

Supplementary Table 4. Evolutionary proximity between Archaea and Bacteria by differential gene, site sampling and method.

Gene sampling	Site sampling	Method	Radius	A-B branch length	Norm. A-B branch length	A depth	A-B branch / A depth	B depth	A-B branch / B depth	Mean A-A distance	Mean B-B distance	Mean A-B distance	Relative A-B distance
global	conserved	ASTRAL	0.971	0.122	0.126	0.9	0.136	0.91	0.134	1.527	1.604	1.957	1.563
global	conserved	CONCAT	0.992	0.126	0.127	0.873	0.144	0.932	0.135	1.514	1.639	1.99	1.596
global	random	ASTRAL	1.773	0.152	0.086	1.405	0.108	1.717	0.089	2.343	3.015	3.274	1.517
global	random	CONCAT	1.801	0.159	0.088	1.436	0.111	1.739	0.091	2.356	3.079	3.35	1.547
r-proteins	all	ASTRAL	3.018	2.528	0.838	1.589	1.591	1.767	1.431	2.449	3.068	5.815	4.501
r-proteins	all	CONCAT	3.333	2.324	0.697	1.823	1.275	2.2	1.057	2.51	3.218	6.348	4.99
global	all	FastTree	1.941	0.21	0.108	1.393	0.151	1.858	0.113	2.509	3.233	3.522	1.529

Letters “A” and “B” refer to Archaea and Bacteria, respectively. Two metrics were assessed: the length of the branch connecting LCA of Archaea and LCA of Bacteria, either original or normalized by the tree radius (calculated as the median of root-to-tip distances of all taxa); and the relative Archaea-Bacteria distance, calculated as: mean(A-B)2 / (mean(A-A) × mean(B-B)), in which each distance is the sum of lengths of branches connecting one tip to another. In addition, the depths of the Archaea and Bacteria clades, calculated as the median of root-to-tip distances of all taxa in each clade, and the length of the branch connecting the two LCAs divided by the clade depth, are provided, to reflect the proximity between Archaea and Bacteria as compared to the dimension of each clade.

Supplementary Table 5. Evolutionary proximity between Archaea and Bacteria with the removal of CPR taxa.

Gene sampling	Site sampling	Method	Radius	A-B branch length	Norm. A-B branch length	A depth	A-B branch / A depth	B depth	A-B branch / B depth	Mean A-A distance	Mean B-B distance	Mean A-B distance	Relative A-B distance
**CPR clade pruned from tree**													
global	conserved	ASTRAL	0.923	0.181	0.196	0.9	0.201	0.827	0.219	1.527	1.497	1.937	1.642
global	conserved	CONCAT	0.958	0.16	0.167	0.873	0.184	0.878	0.183	1.514	1.53	1.972	1.679
global	random	ASTRAL	1.744	0.249	0.143	1.405	0.177	1.637	0.152	2.343	2.907	3.287	1.587
global	random	CONCAT	1.804	0.201	0.112	1.436	0.14	1.722	0.117	2.356	2.983	3.369	1.615
r-proteins	all	ASTRAL	2.966	2.623	0.884	1.589	1.65	1.656	1.584	2.449	2.867	5.783	4.764
r-proteins	all	CONCAT	3.272	2.324	0.71	1.823	1.275	2.134	1.089	2.51	3.023	6.271	5.183
de novo **tree from CPR-free alignment**													
global	conserved	ASTRAL	0.956	0.204	0.213	0.871	0.234	0.853	0.239	1.556	1.512	1.946	1.61
global	conserved	CONCAT	0.98	0.141	0.144	0.888	0.159	0.91	0.155	1.543	1.566	2.009	1.67
global	random	ASTRAL	1.795	0.298	0.166	1.361	0.219	1.671	0.178	2.381	2.931	3.307	1.567
global	random	CONCAT	1.827	0.197	0.108	1.436	0.137	1.747	0.113	2.394	3.031	3.401	1.593
r-proteins	all	ASTRAL	3.308	3.115	0.942	1.563	1.993	1.769	1.761	2.593	3.046	6.361	5.123
r-proteins	all	CONCAT	3.272	2.511	0.767	1.87	1.343	2.039	1.232	2.54	3.064	6.358	5.193

The results of two experimental groups are shown. Upper: The CPR clade was pruned from the trees discussed in Fig. 3 and Supplementary Table 4. Lower: The CPR sequences were removed from the dataset, and trees were re-built. The definitions of column names follow Supplementary Table 4.

Supplementary Table 6. Evolutionary proximity between Archaea and Bacteria by ASTRAL with differential gene tree sampling.

Gene sampling	No. of genes	Site sampling	Radius	A-B branch length	Norm. A-B branch length	A depth	A-B branch / A depth	B depth	A-B branch / B depth	Mean A-A distance	Mean B-B distance	Mean A-B distance	Relative A-B distance
qts > 0.5	322	conserved	0.863	0.132	0.153	0.859	0.154	0.795	0.166	1.426	1.387	1.801	1.641
qts > 0.5	322	random	1.788	0.184	0.103	1.474	0.125	1.713	0.108	2.4	2.969	3.349	1.574
qts > 0.67	171	conserved	0.887	0.241	0.272	0.928	0.26	0.756	0.319	1.538	1.328	1.929	1.822
qts > 0.67	171	random	1.905	0.349	0.183	1.621	0.215	1.742	0.2	2.606	3.029	3.667	1.704
qts > 0.75	93	conserved	0.831	0.278	0.334	0.917	0.303	0.679	0.409	1.5	1.2	1.888	1.98
qts > 0.75	93	random	2.029	0.48	0.237	1.708	0.281	1.793	0.268	2.713	3.127	3.952	1.841
qts > 0.8	64	conserved	0.812	0.276	0.34	0.957	0.288	0.663	0.416	1.494	1.186	1.886	2.007
qts > 0.8	64	random	2.02	0.47	0.233	1.803	0.261	1.784	0.264	2.804	3.112	4.006	1.839

The 381 gene trees were subsampled based on their quartet scores (qts) vs. the species tree. Larger qts indicates higher topological concordance. The definitions of column names follow Supplementary Table 4.

Supplementary Table 7. Evolutionary proximity between Archaea and Bacteria with 1,000 taxa.

Gene sampling	Site sampling	Site model	Radius	A-B branch length	Norm. A-B branch length	A depth	A-B branch / A depth	B depth	A-B branch / B depth	Mean A-A distance	Mean B-B distance	Mean A-B distance	Relative A-B distance
global	all	CONCAT	1.333	0.17	0.128	1.047	0.162	1.26	0.135	1.856	2.225	2.518	1.535
global	conserved	Gamma	0.706	0.11	0.155	0.669	0.164	0.65	0.169	1.171	1.162	1.475	1.598
global	conserved	FreeRate	0.615	0.094	0.153	0.59	0.159	0.566	0.166	1.02	1.012	1.29	1.614
global	conserved	PMSF	0.982	0.168	0.171	1.049	0.16	0.885	0.19	1.833	1.66	2.197	1.586
global	random	Gamma	1.323	0.141	0.107	1.043	0.135	1.264	0.112	1.852	2.212	2.483	1.505
global	random	FreeRate	1.441	0.149	0.104	1.147	0.13	1.379	0.108	2.012	2.419	2.714	1.514
global	random	PMSF	2.203	0.259	0.118	1.836	0.141	2.083	0.125	3.268	3.67	4.234	1.495
r-proteins	all	Gamma	2.079	1.719	0.827	1.201	1.432	1.22	1.409	1.716	2.092	4.184	4.874
r-proteins	all	FreeRate	1.939	1.554	0.802	1.119	1.389	1.163	1.337	1.583	1.923	3.86	4.894
r-proteins	all	PMSF	4.048	4.237	1.047	1.775	2.387	1.934	2.191	2.857	3.286	8.016	6.845

The original 10,575 genomes were downsampled to 1,000 (see Methods), which allowed for phylogenetic reconstruction using the more expensive site heterogeneous model PMSF, as compared to the simpler site homogeneous models Gamma and FreeRate. The definitions of column names follow Supplementary Table 4.

Supplementary Table 8. Divergence time estimation results by maximum likelihood using one calibration.

Genes & sites	Method	Reps. passed	LUCA	CPR split from Bacteria	Archaea diversification	Non-CPR Bacteria diversification	CPR diversification
**General results**							
conserved	ASTRAL	9	4.228 ± 0.046 (4.206)	3.958 ± 0.043 (3.937)	3.9 ± 0.043 (3.879)	3.32 ± 0.036 (3.302)	3.772 ± 0.041 (3.752)
	CONCAT	8	4.181 ± 0.063 (4.147)	3.894 ± 0.058 (3.862)	3.855 ± 0.058 (3.824)	3.398 ± 0.051 (3.371)	3.736 ± 0.056 (3.705)
random	ASTRAL	8	3.631 ± 0.054 (3.618)	3.494 ± 0.052 (3.482)	3.295 ± 0.049 (3.284)	3.229 ± 0.048 (3.218)	3.276 ± 0.049 (3.265)
	CONCAT	7	3.654 ± 0.021 (3.7)	3.515 ± 0.02 (3.56)	3.28 ± 0.019 (3.321)	3.419 ± 0.02 (3.463)	3.299 ± 0.019 (3.341)
r-proteins	ASTRAL	10	7.068 ± 0.113 (7.174)	4.053 ± 0.065 (4.113)	3.470 ± 0.056 (3.522)	3.542 ± 0.057 (3.595)	3.945 ± 0.063 (4.004)
	CONCAT	9	7.012 ± 0.120 (6.963)	4.219 ± 0.072 (4.185)	3.689 ± 0.063 (3.659)	-	3.441 ± 0.059 (3.413)
**Moving root on ASTRAL tree**							
conserved	25%	7	4.211 ± 0.01 (4.213)	3.881 ± 0.009 (3.882)	3.986 ± 0.01 (3.987)	3.3 ± 0.008 (3.301)	3.716 ± 0.009 (3.718)
	75%	10	4.218 ± 0.066 (4.185)	4.043 ± 0.064 (4.013)	3.829 ± 0.06 (3.8)	3.337 ± 0.052 (3.311)	3.831 ± 0.06 (3.802)
random	25%	8	3.635 ± 0.058 (3.598)	3.456 ± 0.055 (3.421)	3.379 ± 0.054 (3.345)	3.216 ± 0.051 (3.184)	3.252 ± 0.052 (3.219)
	75%	10	3.589 ± 0.035 (3.568)	3.21 ± 0.031 (3.191)	3.21 ± 0.031 (3.191)	3.22 ± 0.031 (3.201)	3.279 ± 0.032 (3.26)
r-proteins	25%	10	7.131 ± 0.109 (7.066)	3.936 ± 0.06 (3.9)	3.638 ± 0.056 (3.605)	3.507 ± 0.054 (3.475)	3.848 ± 0.059 (3.813)
	75%	9	6.87 ± 0.056 (6.853)	4.186 ± 0.034 (4.177)	3.328 ± 0.027 (3.32)	3.558 ± 0.029 (3.55)	4.044 ± 0.033 (4.035)
**PMSF vs. Gamma on 1k taxa**							
all	Gamma	10	3.744 ± 0.017 (3.74)	3.525 ± 0.016 (3.521)	3.398 ± 0.016 (3.393)	3.271 ± 0.015 (3.267)	3.168 ± 0.015 (3.164)
conserved	Gamma	10	4.503 ± 0.02 (4.517)	4.156 ± 0.018 (4.168)	4.165 ± 0.019 (4.178)	3.509 ± 0.016 (3.52)	3.841 ± 0.017 (3.853)
	PMSF	10	4.553 ± 0.02 (4.543)	4.158 ± 0.019 (4.148)	4.265 ± 0.019 (4.255)	3.271 ± 0.015 (3.264)	3.869 ± 0.017 (3.86)
random	Gamma	10	3.718 ± 0.015 (3.712)	3.541 ± 0.014 (3.536)	3.419 ± 0.013 (3.414)	3.253 ± 0.013 (3.248)	3.205 ± 0.013 (3.2)
	PMSF	10	3.682 ± 0.015 (3.673)	3.486 ± 0.015 (3.477)	3.408 ± 0.014 (3.399)	3.192 ± 0.013 (3.185)	3.166 ± 0.013 (3.158)
r-proteins	Gamma	10	7.487 ± 0.03 (7.463)	4.416 ± 0.018 (4.402)	4.038 ± 0.016 (4.025)	4.224 ± 0.017 (4.211)	4.218 ± 0.017 (4.204)
	PMSF	10	9.219 ± 0.034 (9.19)	4.565 ± 0.017 (4.55)	3.939 ± 0.014 (3.926)	-	4.349 ± 0.016 (4.336)

The Cyanobacteria/Melainabacteria split was constrained to 2.5-2.6 Ga. For each setting, ten replicates were executed and the number of replicates that passed the gradient check was reported, and the means and standard deviations were calculated based on those replicates. The run with the best

likelihood in all replicates was reported separately in parentheses. Estimated ages of five early evolutionary events were reported. The “non-CPR Bacteria diversification” field was left blank if the corresponding tree topology did not support the monophyly of non-CPR Bacteria.

Supplementary Table 9. Divergence time estimation results by maximum likelihood using alternative calibrations.

Site sampling			conserved			random		
Name	Node	Range	Pass	LUCA	Non-CPR Bacteria diversification	Pass	LUCA	Non-CPR Bacteria diversification
Photosynthetic eukaryotes	Cyanobacteria LCA	>1.03	7	4.252 ± 0.076 (4.355)	3.338 ± 0.059 (3.419)	8	3.639 ± 0.06 (3.73)	3.237 ± 0.053 (3.318)
	Alphaproteobacteria LCA		9	4.232 ± 0.055 (4.201)	3.323 ± 0.043 (3.298)	6	3.589 ± 0.004 (3.587)	3.193 ± 0.004 (3.191)
	Alphaproteobacteria origin		9	4.242 ± 0.065 (4.201)	3.33 ± 0.051 (3.298)	9	3.625 ± 0.059 (3.728)	3.225 ± 0.052 (3.316)
	Cyanobacteria LCA and Alphaproteobacteria LCA		9	4.25 ± 0.068 (4.203)	3.337 ± 0.053 (3.3)	7	3.664 ± 0.071 (3.713)	3.259 ± 0.063 (3.303)
	Cyanobacteria LCA and Alphaproteobacteria origin		10	4.228 ± 0.054 (4.215)	3.32 ± 0.042 (3.309)	10	3.618 ± 0.058 (3.73)	3.218 ± 0.052 (3.318)
Akinetes-forming cyanobacteria	Nostocales origin	>1.2	10	5.534 ± 0 (5.534)	4.339 ± 0 (4.339)	7	4.695 ± 0 (4.695)	4.169 ± 0 (4.169)
		>1.5	10	6.253 ± 0 (6.253)	4.903 ± 0 (4.903)	7	5.302 ± 0 (5.302)	4.707 ± 0 (4.707)
		>1.9	10	7.163 ± 0 (7.163)	5.615 ± 0 (5.615)	9	6.084 ± 0 (6.084)	5.401 ± 0 (5.401)
		>2.1	10	7.594 ± 0 (7.594)	5.953 ± 0 (5.953)	7	6.459 ± 0 (6.459)	5.735 ± 0 (5.735)
Aphid-*Buchnera* symbiosis	*Buchnera*/*Wigglesworthia* split	>0.084	6	4.202 ± 0.002 (4.201)	3.299 ± 0.002 (3.298)	8	3.622 ± 0.057 (3.73)	3.222 ± 0.051 (3.318)
		>0.164	7	4.301 ± 0.076 (4.203)	3.376 ± 0.059 (3.299)	8	3.606 ± 0.05 (3.59)	3.208 ± 0.044 (3.193)
Photosynthetic eukaryotes and Aphid-*Buchnera* symbiosis	Cyanobacteria LCA and Alphaproteobacteria origin	>1.03	9	4.241 ± 0.058 (4.297)	3.33 ± 0.045 (3.373)	8	3.593 ± 0.011 (3.588)	3.196 ± 0.01 (3.191)
	Buchnera/*Wigglesworthia* split	>0.164						

One or more calibrations were included in addition to the Cyanobacteria/Melainabacteria calibration, as described in each row. The definitions of column names follow Supplementary Table 8.

Supplementary Table 10. Divergence time estimation results by Bayesian inference.

Genes	Constraint	Prior dist.	Clock model	MCMC		Age of LUCA (Ga)					Clock rate		C.V.	
				States (M)	Burn-in (M)	ESS	mean	median	95% low	95% high	ESS	mean	ESS	mean
global	narrow	norm	strict	10	1	1100	3.759	3.759	3.627	3.889	1337	0.288	-	-
	narrow	norm	ucld	50	5	431	3.821	3.816	3.56	4.089	526	0.289	449	0.176
	wide	ln	strict	10	1	5666	3.71	3.625	3.379	4.264	6996	0.293	-	-
	wide	ln	ucld	20	2	1007	3.768	3.7	3.312	4.351	1320	0.295	173	0.175
r-proteins	narrow	norm	strict	10	1	1390	7.45	7.448	7.127	7.765	1230	0.22	-	-
	narrow	norm	ucld	100	10	283	7.389	7.35	6.08	8.782	206	0.226	171	0.254
	wide	ln	strict	10	1	7645	7.347	7.198	6.64	8.455	7249	0.224	-	-
	wide	ln	ucld	50	10	250	7.362	7.254	5.782	9.142	296	0.229	157	0.255

Input data were 100 taxa and 5,000 randomly sampled amino acid sites. Comparative analysis was performed using two clock models: strict clock or uncorrelated lognormal relaxed clock (ucld); two prior distributions of the time constraint of the Cyanobacteria/Melainabacteria split: “narrow”: a normal distribution which is narrower and based on previous estimates, and “wide”: a lognormal distribution which is wider and based on palaeobiological and geological evidence. We reported the estimated age of LUCA, the clock rate and its coefficient of variance (C.V., only for the relaxed clock model), which is a measurement of the “clock-likeness” of data (smaller is better).

Supplementary Table 11. Evolutionary proximity between Archaea and Bacteria with 187 extra genomes.

Gene sampling	Site sampling	Method	Radius	A-B branch length	Norm. A-B branch length	A depth	A-B branch / A depth	B depth	A-B branch / B depth	Mean A-A distance	Mean B-B distance	Mean A-B distance	Relative A-B distance
global	conserved	ASTRAL	0.975	0.124	0.127	0.902	0.137	0.914	0.135	1.536	1.609	1.973	1.575
global	conserved	CONCAT	1.007	0.126	0.125	0.882	0.143	0.95	0.133	1.533	1.654	1.999	1.576
global	random	ASTRAL	1.787	0.151	0.085	1.406	0.108	1.734	0.087	2.35	3.025	3.3	1.531
global	random	CONCAT	1.809	0.163	0.09	1.391	0.117	1.759	0.093	2.354	3.069	3.321	1.526

The original 10,575 genomes sampled in March 2017 plus the 187 new genomes sampled in May 2019 which represent previously missing or underrepresented NCBI and GTDB phyla were included in this analysis. The definitions of column names follow Supplementary Table 4.

Supplementary References

1. Gamez, J. E., Esteban Gamez, J., Modave, F. & Kosheleva, O. Selecting the most representative sample is NP-hard: Need for expert (fuzzy) knowledge. in *2008 IEEE International Conference on Fuzzy Systems (IEEE World Congress on Computational Intelligence)* (2008). doi:10.1109/fuzzy.2008.4630502
2. Cornuejols, G., Fisher, M. L. & Nemhauser, G. L. Exceptional Paper—Location of Bank Accounts to Optimize Float: An Analytic Study of Exact and Approximate Algorithms. *Manage. Sci.* **23**, 789–810 (1977).
3. Ondov, B. D. *et al.* Mash: fast genome and metagenome distance estimation using MinHash. *Genome Biol.* **17**, 132 (2016).
4. Bien, J. & Tibshirani, R. Prototype selection for interpretable classification. *Ann. Appl. Stat.* **5**, 2403–2424 (2011).
5. Massart, D., Plastria, F. & Kaufman, L. Non-hierarchical clustering with masloc. *Pattern Recognit.* **16**, 507–516 (1983).
6. Robinson, D. F. & Foulds, L. R. Comparison of phylogenetic trees. *Math. Biosci.* **53**, 131–147 (1981).
7. Stamatakis, A. Phylogenetic models of rate heterogeneity: a high performance computing perspective. in *Proceedings 20th IEEE International Parallel & Distributed Processing Symposium* (2006). doi:10.1109/ipdps.2006.1639535
8. Ciccarelli, F. D. *et al.* Toward automatic reconstruction of a highly resolved tree of life. *Science* **311**, 1283–1287 (2006).
9. Gogarten, J. P., Peter Gogarten, J., Ford Doolittle, W. & Lawrence, J. G. Prokaryotic Evolution in Light of Gene Transfer. *Molecular Biology and Evolution* **19**, 2226–2238 (2002).
10. Parks, D. H. *et al.* A standardized bacterial taxonomy based on genome phylogeny substantially revises the tree of life. *Nat. Biotechnol.* (2018).
11. Hug, L. A. *et al.* A new view of the tree of life. *Nat Microbiol* **1**, 16048 (2016).
12. Lartillot, N., Brinkmann, H. & Philippe, H. Suppression of long-branch attraction artefacts in the animal phylogeny using a site-heterogeneous model. *BMC Evol. Biol.* **7 Suppl 1**, S4 (2007).

13. Wang, H.-C., Minh, B. Q., Susko, E. & Roger, A. J. Modeling Site Heterogeneity with Posterior Mean Site Frequency Profiles Accelerates Accurate Phylogenomic Estimation. *Syst. Biol.* **67**, 216–235 (2018).

14. Quang, L. S., Gascuel, O. & Lartillot, N. Empirical profile mixture models for phylogenetic reconstruction. *Bioinformatics* **24**, 2317–2323 (2008).

15. Soubrier, J. *et al.* The influence of rate heterogeneity among sites on the time dependence of molecular rates. *Mol. Biol. Evol.* **29**, 3345–3358 (2012).

16. Price, M. N., Dehal, P. S. & Arkin, A. P. FastTree 2 – Approximately Maximum-Likelihood Trees for Large Alignments. *PLoS One* **5**, e9490 (2010).

17. McDonald, D. *et al.* An improved Greengenes taxonomy with explicit ranks for ecological and evolutionary analyses of bacteria and archaea. *ISME J.* **6**, 610–618 (2012).

18. Liu, K., Linder, C. R. & Warnow, T. RAxML and FastTree: comparing two methods for large-scale maximum likelihood phylogeny estimation. *PLoS One* **6**, e27731 (2011).

19. Zhou, X., Shen, X.-X., Hittinger, C. T. & Rokas, A. Evaluating Fast Maximum Likelihood-Based Phylogenetic Programs Using Empirical Phylogenomic Data Sets. *Mol. Biol. Evol.* **35**, 486–503 (2018).

20. Sayyari, E., Whitfield, J. B. & Mirarab, S. Fragmentary Gene Sequences Negatively Impact Gene Tree and Species Tree Reconstruction. *Mol. Biol. Evol.* **34**, 3279–3291 (2017).

21. Federhen, S. The NCBI Taxonomy database. *Nucleic Acids Research* **40**, D136–D143 (2012).

22. Sayyari, E. & Mirarab, S. Fast Coalescent-Based Computation of Local Branch Support from Quartet Frequencies. *Mol. Biol. Evol.* **33**, 1654–1668 (2016).

23. Bowers, R. M. *et al.* Minimum information about a single amplified genome (MISAG) and a metagenome-assembled genome (MIMAG) of bacteria and archaea. *Nature Biotechnology* **35**, 725–731 (2017).

24. Eme, L., Spang, A., Lombard, J., Stairs, C. W. & Ettema, T. J. G. Archaea and the origin of eukaryotes. *Nat. Rev. Microbiol.* **15**, 711–723 (2017).

25. Castelle, C. J. & Banfield, J. F. Major New Microbial Groups Expand Diversity and Alter our Understanding of the Tree of Life. *Cell* **172**, 1181–1197 (2018).

26. Petitjean, C., Deschamps, P., López-García, P. & Moreira, D. Rooting the Domain Archaea by Phylogenomic Analysis Supports the Foundation of the New Kingdom Proteoarchaeota. *Genome Biol. Evol.*

7, 191–204 (2015).

27. Zaremba-Niedzwiedzka, K. *et al.* Asgard archaea illuminate the origin of eukaryotic cellular complexity. *Nature* **541**, 353 (2017).
28. Guy, L. & Ettema, T. J. G. The archaeal 'TACK' superphylum and the origin of eukaryotes. *Trends Microbiol.* **19**, 580–587 (2011).
29. Spang, A., Caceres, E. F. & Ettema, T. J. G. Genomic exploration of the diversity, ecology, and evolution of the archaeal domain of life. *Science* **357**, (2017).
30. Rinke, C. *et al.* Insights into the phylogeny and coding potential of microbial dark matter. *Nature* **499**, 431 (2013).
31. Williams, T. A. *et al.* Integrative modeling of gene and genome evolution roots the archaeal tree of life. *Proc. Natl. Acad. Sci. U. S. A.* **114**, E4602–E4611 (2017).
32. Brown, C. T. *et al.* Unusual biology across a group comprising more than 15% of domain Bacteria. *Nature* **523**, 208 (2015).
33. Wrighton, K. C. *et al.* Fermentation, Hydrogen, and Sulfur Metabolism in Multiple Uncultivated Bacterial Phyla. *Science* **337**, 1661–1665 (2012).
34. Anantharaman, K. *et al.* Thousands of microbial genomes shed light on interconnected biogeochemical processes in an aquifer system. *Nat. Commun.* **7**, 13219 (2016).
35. Xu, Y. & Glansdorff, N. Was our ancestor a hyperthermophilic procaryote? *Comp. Biochem. Physiol. A Mol. Integr. Physiol.* **133**, 677–688 (2002).
36. Reysenbach, A.-L. & Shock, E. Merging genomes with geochemistry in hydrothermal ecosystems. *Science* **296**, 1077–1082 (2002).
37. Battistuzzi, F. U. & Hedges, S. B. A Major Clade of Prokaryotes with Ancient Adaptations to Life on Land. *Mol. Biol. Evol.* **26**, 335–343 (2008).
38. Tamaki, H. *et al.* Armatimonas rosea gen. nov., sp. nov., of a novel bacterial phylum, Armatimonadetes phyl. nov., formally called the candidate phylum OP10. *Int. J. Syst. Evol. Microbiol.* **61**, 1442–1447 (2011).
39. Barns, S. M., Delwiche, C. F., Palmer, J. D. & Pace, N. R. Perspectives on archaeal diversity, thermophily and monophyly from environmental rRNA sequences. *Proc. Natl. Acad. Sci. U. S. A.* **93**, 9188–9193 (1996).

40. Klenk, H. P. *et al.* RNA polymerase of Aquifex pyrophilus: implications for the evolution of the bacterial rpoBC operon and extremely thermophilic bacteria. *J. Mol. Evol.* **48**, 528–541 (1999).

41. Gruber, T. M. & Bryant, D. A. Characterization of the group 1 and group 2 sigma factors of the green sulfur bacterium Chlorobium tepidum and the green non-sulfur bacterium Chloroflexus aurantiacus. *Arch. Microbiol.* **170**, 285–296 (1998).

42. Coenye, T. & Vandamme, P. A genomic perspective on the relationship between the Aquificales and the epsilon-Proteobacteria. *Syst. Appl. Microbiol.* **27**, 313–322 (2004).

43. Jumas-Bilak, E., Roudière, L. & Marchandin, H. Description of 'Synergistetes' phyl. nov. and emended description of the phylum 'Deferribacteres' and of the family Syntrophomonadaceae, phylum 'Firmicutes'. *Int. J. Syst. Evol. Microbiol.* **59**, 1028–1035 (2009).

44. Mukherjee, S. *et al.* 1,003 reference genomes of bacterial and archaeal isolates expand coverage of the tree of life. *Nat. Biotechnol.* **35**, 676–683 (2017).

45. Wolf, M., Müller, T., Dandekar, T. & Pollack, J. D. Phylogeny of Firmicutes with special reference to Mycoplasma (Mollicutes) as inferred from phosphoglycerate kinase amino acid sequence data. *Int. J. Syst. Evol. Microbiol.* **54**, 871–875 (2004).

46. Strömpl, C. *et al.* Reclassification of Clostridium quercicolum as Dendrosporobacter quercicolus gen. nov., comb. nov. *Int. J. Syst. Evol. Microbiol.* **50 Pt 1**, 101–106 (2000).

47. Gupta, R. S. & Gao, B. Phylogenomic analyses of clostridia and identification of novel protein signatures that are specific to the genus Clostridium sensu stricto (cluster I). *Int. J. Syst. Evol. Microbiol.* **59**, 285–294 (2009).

48. Yutin, N. & Galperin, M. Y. A genomic update on clostridial phylogeny: Gram-negative spore formers and other misplaced clostridia. *Environ. Microbiol.* **15**, 2631–2641 (2013).

49. Zhang, X. *et al.* Petroclostridium xylanilyticum gen. nov., sp. nov., a xylan-degrading bacterium isolated from an oilfield, and reclassification of clostridial cluster III members into four novel genera in a new Hungateiclostridiaceae fam. nov. *Int. J. Syst. Evol. Microbiol.* (2018). doi:10.1099/ijsem.0.002966

50. Nouioui, I. *et al.* Genome-Based Taxonomic Classification of the Phylum. *Front. Microbiol.* **9**, 2007 (2018).

51. Shih, P. M., Ward, L. M. & Fischer, W. W. Evolution of the 3-hydroxypropionate bicycle and recent transfer

of anoxygenic photosynthesis into the Chloroflexi. *Proc. Natl. Acad. Sci. U. S. A.* **114**, 10749–10754 (2017).

52. Shih, P. M. Photosynthesis and early Earth. *Curr. Biol.* **25**, R855–9 (2015).
53. Yarza, P. *et al.* Uniting the classification of cultured and uncultured bacteria and archaea using 16S rRNA gene sequences. *Nat. Rev. Microbiol.* **12**, 635–645 (2014).
54. Takaichi, S., Maoka, T., Takasaki, K. & Hanada, S. Carotenoids of Gemmatimonas aurantiaca (Gemmatimonadetes): identification of a novel carotenoid, deoxyoscillol 2-rhamnoside, and proposed biosynthetic pathway of oscillol 2,2'-dirhamnoside. *Microbiology* **156**, 757–763 (2010).
55. Eisen, J. A. *et al.* The complete genome sequence of Chlorobium tepidum TLS, a photosynthetic, anaerobic, green-sulfur bacterium. *Proc. Natl. Acad. Sci. U. S. A.* **99**, 9509–9514 (2002).
56. Campbell, B. J., Engel, A. S., Porter, M. L. & Takai, K. The versatile epsilon-proteobacteria: key players in sulphidic habitats. *Nat. Rev. Microbiol.* **4**, 458–468 (2006).
57. Davín, A. A. *et al.* Gene transfers can date the tree of life. *Nat Ecol Evol* **2**, 904–909 (2018).
58. Blair Hedges, S. & Kumar, S. *The Timetree of Life*. (OUP Oxford, 2009).
59. Di Rienzi, S. C. *et al.* The human gut and groundwater harbor non-photosynthetic bacteria belonging to a new candidate phylum sibling to Cyanobacteria. *eLife Sciences* **2**, e01102 (2013).
60. Soo, R. M., Hemp, J., Parks, D. H., Fischer, W. W. & Hugenholtz, P. On the origins of oxygenic photosynthesis and aerobic respiration in Cyanobacteria. *Science* **355**, 1436–1440 (2017).
61. Luo, G. *et al.* Rapid oxygenation of Earth's atmosphere 2.33 billion years ago. *Science Advances* **2**, e1600134 (2016).
62. Shih, P. M., Hemp, J., Ward, L. M., Matzke, N. J. & Fischer, W. W. Crown group Oxyphotobacteria postdate the rise of oxygen. *Geobiology* **15**, 19–29 (2017).
63. Dodd, M. S. *et al.* Evidence for early life in Earth's oldest hydrothermal vent precipitates. *Nature* **543**, 60 (2017).
64. Marin, J., Battistuzzi, F. U., Brown, A. C. & Hedges, S. B. The Timetree of Prokaryotes: New Insights into Their Evolution and Speciation. *Mol. Biol. Evol.* **34**, 437–446 (2017).
65. Dalrymple, G. B. & Brent Dalrymple, G. The age of the Earth in the twentieth century: a problem (mostly) solved. *Geological Society, London, Special Publications* **190**, 205–221 (2001).

66. Zimorski, V., Ku, C., Martin, W. F. & Gould, S. B. Endosymbiotic theory for organelle origins. *Curr. Opin. Microbiol.* **22**, 38–48 (2014).

67. Gibson, T. M. *et al.* Precise age of Bangiomorpha pubescens dates the origin of eukaryotic photosynthesis. *Geology* **46**, 135–138 (2018).

68. Moreira, D., Le Guyader, H. & Philippe, H. The origin of red algae and the evolution of chloroplasts. *Nature* **405**, 69–72 (2000).

69. Ochoa de Alda, J. A. G., Esteban, R., Diago, M. L. & Houmard, J. The plastid ancestor originated among one of the major cyanobacterial lineages. *Nat. Commun.* **5**, 4937 (2014).

70. Ponce-Toledo, R. I. *et al.* An Early-Branching Freshwater Cyanobacterium at the Origin of Plastids. *Curr. Biol.* **27**, 386–391 (2017).

71. Roger, A. J., Muñoz-Gómez, S. A. & Kamikawa, R. The Origin and Diversification of Mitochondria. *Curr. Biol.* **27**, R1177–R1192 (2017).

72. Martijn, J., Vosseberg, J., Guy, L., Offre, P. & Ettema, T. J. G. Deep mitochondrial origin outside the sampled alphaproteobacteria. *Nature* **557**, 101–105 (2018).

73. Tomitani, A., Knoll, A. H., Cavanaugh, C. M. & Ohno, T. The evolutionary diversification of cyanobacteria: molecular-phylogenetic and paleontological perspectives. *Proc. Natl. Acad. Sci. U. S. A.* **103**, 5442–5447 (2006).

74. Butterfield, N. J. Proterozoic photosynthesis - a critical review. *Palaeontology* **58**, 953–972 (2015).

75. David, L. A. & Alm, E. J. Rapid evolutionary innovation during an Archaean genetic expansion. *Nature* **469**, 93–96 (2011).

76. Betts, H. C. *et al.* Integrated genomic and fossil evidence illuminates life's early evolution and eukaryote origin. *Nature Ecology & Evolution* (2018). doi:10.1038/s41559-018-0644-x

77. Magnabosco, C., Moore, K. R., Wolfe, J. M. & Fournier, G. P. Dating phototrophic microbial lineages with reticulate gene histories. *Geobiology* **16**, 179–189 (2018).

78. Horodyski, R. J. & Allan Donaldson, J. Microfossils from the Middle Proterozoic Dismal Lakes Groups, Arctic Canada. *Precambrian Research* **11**, 125–159 (1980).

79. Golubic, S., Sergeev, V. N. & Knoll, A. H. Mesoproterozoic Archaeoellipsoides: akinetes of heterocystous

cyanobacteria. *Lethaia* **28**, 285–298 (1995).

80. Golubic, S. & Hofmann, H. J. Comparison of Holocene and mid-Precambrian Entophysalidaceae (Cyanophyta) in stromatolitic algal mats; cell division and degradation. *J. Paleontol.* **50**, 1074–1082 (1976).
81. Amard, B. & Bertrand-Sarfati, J. Microfossils in 2000 Ma old cherty stromatolites of the Franceville Group, Gabon. *Precambrian Research* **81**, 197–221 (1997).
82. Perkovsky, E. & Wegierek, P. Aphid–Buchnera–Ant symbiosis; or why are aphids rare in the tropics and very rare further south? *Earth and Environmental Science Transactions of the Royal Society of Edinburgh* **107**, 297–310 (2018).
83. Dohlen, C. V. O. N. & Von Dohlen, C. Molecular data support a rapid radiation of aphids in the Cretaceous and multiple origins of host alternation. *Biological Journal of the Linnean Society* **71**, 689–717 (2000).
84. Johnson, K. P. *et al.* Phylogenomics and the evolution of hemipteroid insects. *Proc. Natl. Acad. Sci. U. S. A.* **115**, 12775–12780 (2018).
85. Kaiwa, N. *et al.* Symbiont-Supplemented Maternal Investment Underpinning Host's Ecological Adaptation. *Current Biology* **24**, 2465–2470 (2014).
86. Crowe, S. A. *et al.* Atmospheric oxygenation three billion years ago. *Nature* **501**, 535–538 (2013).
87. Planavsky, N. J. *et al.* Evidence for oxygenic photosynthesis half a billion years before the Great Oxidation Event. *Nature Geoscience* **7**, 283–286 (2014).
88. Nisbet, E. G. *et al.* The age of Rubisco: the evolution of oxygenic photosynthesis. *Geobiology* **5**, 311–335 (2007).
89. Satkoski, A. M., Beukes, N. J., Li, W., Beard, B. L. & Johnson, C. M. A redox-stratified ocean 3.2 billion years ago. *Earth and Planetary Science Letters* **430**, 43–53 (2015).
90. Lyons, T. W., Reinhard, C. T. & Planavsky, N. J. The rise of oxygen in Earth's early ocean and atmosphere. *Nature* **506**, 307 (2014).
91. Schirrmeister, B. E., Sanchez-Baracaldo, P. & Wacey, D. Cyanobacterial evolution during the Precambrian. *International Journal of Astrobiology* **15**, 187–204 (2016).
92. Cardona, T. Thinking twice about the evolution of photosynthesis. *Open Biol.* **9**, 180246 (2019).
93. Ossa Ossa, F. *et al.* Limited oxygen production in the Mesoarchean ocean. *Proc. Natl. Acad. Sci. U. S. A.*

(2019). doi:10.1073/pnas.1818762116

94. Mloszewska, A. M. *et al.* UV radiation limited the expansion of cyanobacteria in early marine photic environments. *Nat. Commun.* **9**, 3088 (2018).

95. Drummond, A. J. & Bouckaert, R. R. *Bayesian Evolutionary Analysis with BEAST*. (Cambridge University Press, 2015).

96. Puigbò, P., Wolf, Y. I. & Koonin, E. V. Search for a 'Tree of Life' in the thicket of the phylogenetic forest. *J. Biol.* **8**, 59 (2009).

97. Hinchliff, C. E. *et al.* Synthesis of phylogeny and taxonomy into a comprehensive tree of life. *Proc. Natl. Acad. Sci. U. S. A.* **112**, 12764–12769 (2015).
