# Supplementary material for: Phylogenomics of 10,575 genomes reveals evolutionary proximity between domains Bacteria and Archaea
Source: Nat Commun. 2019 Dec 2;10:5477. doi: 10.1038/s41467-019-13443-4 (PMC6889312; doi:10.1038/s41467-019-13443-4)
Supplement: Supplementary file 3 — Description of Additional Supplementary Files [file 41467_2019_13443_MOESM3_ESM.pdf]

Description of Additional Supplementary Files

File Name: Supplementary Software 1
Description: Python implementation of prototype selection algorithms
