# Supplementary figures and images for: Phylogenomics of 10,575 genomes reveals evolutionary proximity between domains Bacteria and Archaea
Source: Nat Commun. 2019 Dec 2;10:5477. doi: 10.1038/s41467-019-13443-4 (PMC6889312; doi:10.1038/s41467-019-13443-4)

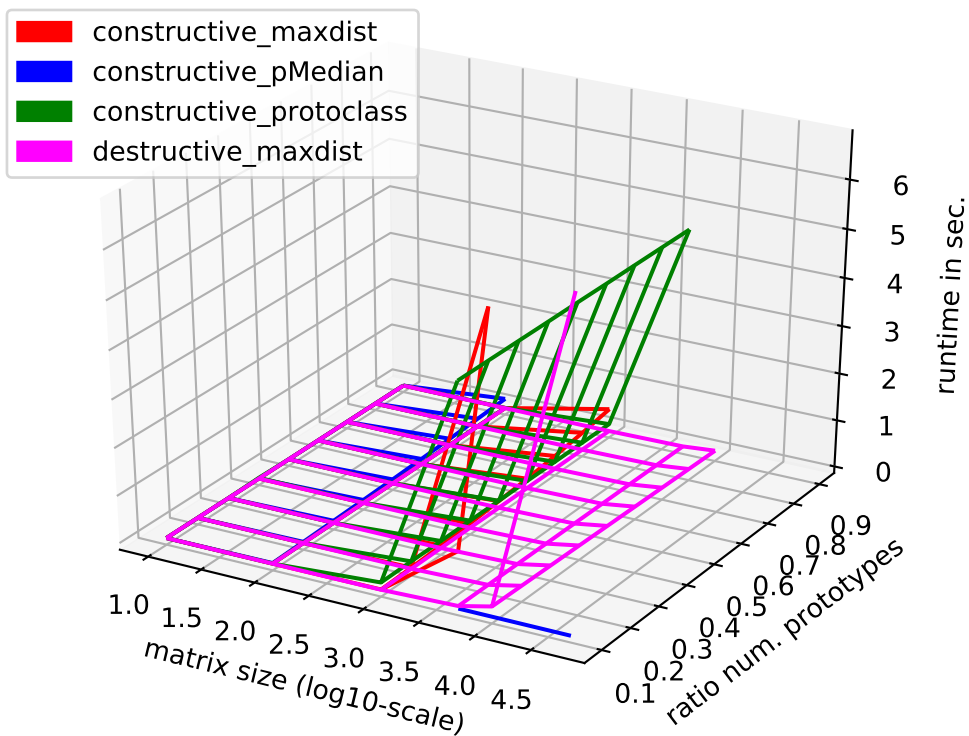

Supplement: Supplementary file 4 — Supplementary Software 1 [file 41467_2019_13443_MOESM4_ESM.zip › Supplementary Software/benchmarks/runtime_prototypeSelection.pdf]
