# Supplementary material for: Phylogenomics of 10,575 genomes reveals evolutionary proximity between domains Bacteria and Archaea
Source: Nat Commun. 2019 Dec 2;10:5477. doi: 10.1038/s41467-019-13443-4 (PMC6889312; doi:10.1038/s41467-019-13443-4)
Supplement: Supplementary file 5 — Reporting Summary [file 41467_2019_13443_MOESM5_ESM.pdf]

nature research

Corresponding author(s): Rob Knight

Last updated by author(s): Nov 2, 2019

Reporting Summary

Nature Research wishes to improve the reproducibility of the work that we publish. This form provides structure for consistency and transparency in reporting. For further information on Nature Research policies, see Authors & Referees and the Editorial Policy Checklist .

Statistics

For all statistical analyses, confirm that the following items are present in the figure legend, table legend, main text, or Methods section.

n/a	Confirmed	
☐	☒	The exact sample size (*n*) for each experimental group/condition, given as a discrete number and unit of measurement
☒	☐	A statement on whether measurements were taken from distinct samples or whether the same sample was measured repeatedly
☐	☒	The statistical test(s) used AND whether they are one- or two-sided<br>*Only common tests should be described solely by name; describe more complex techniques in the Methods section.*
☒	☐	A description of all covariates tested
☒	☐	A description of any assumptions or corrections, such as tests of normality and adjustment for multiple comparisons
☐	☒	A full description of the statistical parameters including central tendency (e.g. means) or other basic estimates (e.g. regression coefficient) AND variation (e.g. standard deviation) or associated estimates of uncertainty (e.g. confidence intervals)
☐	☒	For null hypothesis testing, the test statistic (e.g. *F*, *t*, *r*) with confidence intervals, effect sizes, degrees of freedom and *P* value noted<br>*Give P values as exact values whenever suitable.*
☐	☒	For Bayesian analysis, information on the choice of priors and Markov chain Monte Carlo settings
☒	☐	For hierarchical and complex designs, identification of the appropriate level for tests and full reporting of outcomes
☐	☒	Estimates of effect sizes (e.g. Cohen's *d*, Pearson's *r*), indicating how they were calculated

Our web collection on statistics for biologists contains articles on many of the points above.

Software and code

Policy information about availability of computer code

Data collection	RepoPhlAn commit 03f614c
Data analysis	Software: PhyloPhlAn commit 2c0e61a, NCBI BLAST+ 2.7.1, Mash 1.1.1, Prodigal 2.6.3, USEARCH v9.1.13, CheckM 1.0.7, QIIME 2 2017.12, UPP 2.0, FastTree 2.1.9, TreeShrink 1.1.0, RAxML 8.2.10, IQ-TREE 1.6.1, ASTRAL-MP 5.12.6a, FigTree 1.4.3, iTOL v4, r8s 1.81, BEAST 1.10.4, tax2tree commit 99f19be, scipy 1.1.0, seaborn 0.9.0, scikit-learn 0.19.2, vegan 2.4.4, scikit-bio 0.5.2<br>Custom codes deposited at: https://github.com/biocore/wol

For manuscripts utilizing custom algorithms or software that are central to the research but not yet described in published literature, software must be made available to editors/reviewers. We strongly encourage code deposition in a community repository (e.g. GitHub). See the Nature Research guidelines for submitting code & software for further information.

Data

Policy information about availability of data

All manuscripts must include a data availability statement. This statement should provide the following information, where applicable:

- Accession codes, unique identifiers, or web links for publicly available datasets
- A list of figures that have associated raw data
- A description of any restrictions on data availability

The datasets generated during and analyzed during the current study are publicly available at GitHub: https://github.com/biocore/wol, under the BSD 3-Clause license. All relevant data are available from the authors. The source data underlying Figs. 1-3, and Supplementary Figs. 1, 3-7, 9, 10, 14, 15, 17, 18, 19C-I, 21A, 28, 31 are provided as source data files.

Field-specific reporting

Please select the one below that is the best fit for your research. If you are not sure, read the appropriate sections before making your selection.

☐ Life sciences ☐ Behavioural & social sciences ☒ Ecological, evolutionary & environmental sciences

For a reference copy of the document with all sections, see nature.com/documents/nr-reporting-summary-flat.pdf

Ecological, evolutionary & environmental sciences study design

All studies must disclose on these points even when the disclosure is negative.

Study description	Phylogenomic analyses of publicly available bacterial and archaeal genomes.
Research sample	All non-duplicated bacterial and archaeal genomes retrieved from NCBI GenBank and RefSeq as of 3/7/2017, totalling 86,200.
Sampling strategy	A workflow for selecting 10,575 genomes from the pool is detailed in Methods. In brief: 1) exclude genomes with marker gene count < 100 or contamination > 10%; 2) include the NCBI-defined reference and representative genomes; 3) include genomes that are the only representative of each taxonomic group from phylum to genus; 4) include genomes that are the only representative of each species without defined lineage; 5) use the prototype selection algorithm developed in this work to select genomes by maximizing sum of MinHash distances; 6) for each phylum to genus, and species without classification from phylum to genus, selected one with the highest marker gene count.
Data collection	N/A
Timing and spatial scale	N/A
Data exclusions	To ensure the quality of the alignment, we filtered out extremely gappy sites and sequences: sites with more than 90% gaps were deleted from the alignments, followed by the dropping of sequences with more than 66% gaps.
Reproducibility	N/A
Randomization	N/A
Blinding	N/A

Did the study involve field work? ☐ Yes ☒ No

Reporting for specific materials, systems and methods

We require information from authors about some types of materials, experimental systems and methods used in many studies. Here, indicate whether each material, system or method listed is relevant to your study. If you are not sure if a list item applies to your research, read the appropriate section before selecting a response.

Materials & experimental systems

n/a	Involved in the study	
☒	☐	Antibodies
☒	☐	Eukaryotic cell lines
☒	☐	Palaeontology
☒	☐	Animals and other organisms
☒	☐	Human research participants
☒	☐	Clinical data

Methods

n/a	Involved in the study	
☒	☐	ChIP-seq
☒	☐	Flow cytometry
☒	☐	MRI-based neuroimaging
